# Detoxification of Mycotoxins through Biotransformation

**DOI:** 10.3390/toxins12020121

**Published:** 2020-02-14

**Authors:** Peng Li, Ruixue Su, Ruya Yin, Daowan Lai, Mingan Wang, Yang Liu, Ligang Zhou

**Affiliations:** 1 Department of Plant Pathology, College of Plant Protection, China Agricultural University, Beijing 100193, China; peng_li0429@163.com (P.L.); ruixuesu07@163.com (R.S.); ruyayin1206@163.com (R.Y.); dwlai@cau.edu.cn (D.L.); 2 Department of Applied Chemistry, College of Sciences, China Agricultural University, Beijing 100193, China; wangma@cau.ed.cn; 3 Institute of Food Science and Technology, Chinese Academy of Agricultural Sciences, Beijing 100193, China; liuyang01@caas.cn

**Keywords:** fungi, mycotoxins, phytotoxins, detoxification, biotransformation, living organisms, enzymes, food safety and feed safety

## Abstract

Mycotoxins are toxic fungal secondary metabolites that pose a major threat to the safety of food and feed. Mycotoxins are usually converted into less toxic or non-toxic metabolites through biotransformation that are often made by living organisms as well as the isolated enzymes. The conversions mainly include hydroxylation, oxidation, hydrogenation, de-epoxidation, methylation, glycosylation and glucuronidation, esterification, hydrolysis, sulfation, demethylation and deamination. Biotransformations of some notorious mycotoxins such as alfatoxins, alternariol, citrinin, fomannoxin, ochratoxins, patulin, trichothecenes and zearalenone analogues are reviewed in detail. The recent development and applications of mycotoxins detoxification through biotransformation are also discussed.

## 1. Introduction

Mycotoxins are toxic secondary metabolites produced by fungi mainly belonging to the genera of *Alternaria*, *Aspergillus*, *Fusarium* and *Penicillium*. Mycotoxins may infer serious risks such as carcinogenic, teratogenic, mutagenic and immunosuppressive effects on animal and human health, and lead to economic loses. Several detoxification strategies, including physical [1,2], chemical [3] and biotransformation [4,5,6,7,8] approaches, have been reported. Chemical strageties use acids, bases, oxidizing agents and aldehydes to modify the structure of mycotoxins, which has led to increased public concerns over the chemical residues in food and feed [9,10]. Some physical strategies such as the application of adsorption agents presented no sufficient effect against mycotoxins [11]. Although physical and chemical methods for mycotoxin detoxification met with varying degrees of success, limited efficacy and losses of important nutrients still hamper their applications in food industries [11]. In contrast, biotransformation methods, which have high specificity, produce harmless products, and even lead to complete detoxification under mild and environmentally friendly conditions, have been recognized as the promising solution for decontamination of mycotoxins [6,11]. Living organisms including bacteria, fungi, plants and animals, as well as the isolated enzymes, have been used for biotransformation. They are able to metabolize, destroy or deactivate mycotoxins into stable, less toxic or even nontoxic products [12].

To our knowledge, no detailed review has focused on detoxification of mycotoxins through biotransformation though a series of reviews on the detoxification of certain mycotoxins have been published over the last 20 years [4,5,7,8,13,14,15,16,17,18,19]. Furthermore, significant advances in the knowledge on detoxification of mycotoxins by biotransformation have been achieved recently. This mini-review mainly presents detoxification of mycotoxins through biotransformation of bacteria, fungi, plants and animals, as well as the isolated enzymes, with specific attentions to the reaction types, and detoxifications of some important mycotoxins.

## 2. Reaction Types of Mycotoxin Biotransformation

The reaction types involved in the mycotoxin biotransformations are summarized according to the classes of chemical reactions as follows: (i) hydroxylation; (ii) oxido-reduction between alcohols and ketones; (iii) hydrogenation of the carbon-carbon double bond; (iv) de-epoxidation; (v) methylation; (vi) glycosylation and glucuronidation; (vii) esterification; (viii) hydrolysis; (ix) sulfation; (x) demethylation; (xi) deamination; and (xii) miscellaneous reactions. The substrates, products, bioconversion systems including organisms or enzymes participating in the biotransformations of mycotoxins are summarized in Table 1, Table 2, Table 3, Table 4, Table 5, Table 6, Table 7, Table 8, Table 9, Table 10, Table 11, Table 12 and Table 13. The corresponding conversion reactions are shown in Figures S1–S76 (see Supplementary Materials).

### 2.1. Hydroxylation

Hydroxylation of mycotoxins is a biotransformation process that introduces a hydroxyl group (-OH) into the molecule (Table 1). Regio- and stereoselective introduction of hydroxyl groups at the various positions of the molecule are often facilitated by the enzymes called hydroxylases. Hydroxylation often increases the polarity of mycotoxins, and reduce their toxicity.

Aflatoxin B_1_ (AFB_1_, **1**) was converted to either aflatoxin M_1_ (AFM_1_, **2**) (Figure S1) by channel catfish liver [20] or aflatoxin Q_1_ (AFQ_1_, **3**) by rat liver microsomal cytochrome P450p [21]. AFB_1_ (**1**) was also simultaneously hydroxylated to AFM_1_ (**2**) and AFQ_1_ (**3**) by hepatic microsomal mixed-function oxidase from the rhesus monkey [22]. Similarly, aflatoxin B_2_ (AFB_2_, **4**) was simultaneously converted to aflatoxin M_2_ (AFM_2_, **5**) and aflatoxin Q_2_ (AFQ_2_, **6**) by animal liver microsomes (Figure S2) [14].

Both alternariol (AOH, **7**) and alternariol 9-*O*-methyl ether (AME, **8**) are the main mycotoxins produced by the fungi from the genus *Alternaria*. The monohydroxylated products of AOH (**7**) and AME (**8**) were identified as 2-hydroxy AOH (**9**), 4-hydroxy AOH (**10**), 8-hydroxy AOH (**11**) and 10-hydroxy AOH (**12**), 2-hydroxy AME (**13**), 4-hydroxy AME (**14**), 8-hydroxy AME (**15**) and 10-hydroxy AME (**16**), when AOH (**7**) and AME (**8**) were, respectively, incubated with the microsomes from rat, human and porcine livers (Figures S3 and S4) [23].

Destruxin B (**17**) is a phytotoxin produced by the phytopathogenic fungus *Alternaria brassicae*. Destruxin B (**17**) can be detoxified into hydroxydestruxin B (**18**) by the hydroxylase from cruciferous plants such as *Brassica napus* (Figure S5). It was considered as an important detoxification step made by the host plant [24,25].

Fusaric acid (FA, **19**), also called 5-butylpicolinic acid, is a host non-specific phytotoxin produced by the fungi from the genus *Fusarium* [26]. FA (**19**) was converted to 8-hydroxyfusaric acid (**20**) with hydroxylation by the fungus *Mucor rouxii* (Figure S6) [27].

Ochratoxin A (OTA, **21**) consists of a chlorinated dihydroisocoumarin linked through a 7-carboxyl group to L-phenylalanine by an amide bond. OTA (**21**) was hydroxylated into 7-carboxy-(2′-hydroxy-1-phenylalanine-amide)-5-chloro-8-hydroxy-3,4-dihydro-3*R*- methylisocoumarin (**22**) and a dihydrodiol derivative (**23**) by a Gram-negative bacterium *Phenylobacterium immobile* (Figure S7) [28].

(4*R*)-4*-*Hydroxyochratoxin A (**24**), or 4-hydroxyochratoxin A, was isolated from the urine of rats after injection with OTA (**21**), which indicated that OTA (**21**) was converted to (4*R*)-4*-*hydroxyochratoxin A (**24**) (Figure S8) [29].

OTA (**21**) was hydroxylated into (4*R*)-4-hydroxyochratoxin A (**24**) and (4*S*)-4-hydroxyochratoxin A (**25**) as well as 10-hydroxyochratoxin A (**26**) by rabbit liver microsomes (Figure S9) [30,31].

When ochratoxin B (OTB, **27**) was incubated with horse radish peroxidase (HPR), the high level of the hydroquinone metabolite of ochratoxin (OTHQ, **28**) was produced (Figure S10). It indicated that the hydroquinone redox couple played a significant role in OTB-mediated toxicity [32].

Sterigmatocystin (STC, **29**) was found to be produced by more than 30 fungal species, particularly by *Aspergillus* species such as *A. flavus*, *A. parasiticus*, *A. versicolor* and *A. nidulans* [33]. STC (**29**) has an aflatoxin-like structure including a furofuran ring system. Like AFB_1_ (**1**), STC (**29**) is a liver carcinogen and forms DNA adducts after metabolic activation to an eposide at the furofuran ring. Incubation of STC (**29**) with the hepatic microsomes of humans and rats, 9-hydroxy-STC (**30**) via hydroxylation of STC (**29**) aromatic ring was formed (Figure S11) [34].

T-2 toxin (**31**) was principally produced by different *Fusarium* species, detected in many crops including oats, wheat and barley. Enzyme CYP3A37 hydrolyzed the conversion of T-2 toxin (**31**) to 19-OH T-2 toxin (or called 3′-OH T-2 toxin, **32**) by chicken CYP3A37 reconstituted with NAPH-cytochrome P450 reductase (CPR) and cytochrome b5 produced by *Escherichia coli* (Figure S12) [35]. In addition, the heterologously expressed CYP1A5 in HeLa cells also metabolized T-2 toxin (**31**) into 19-OH T-2 toxin (**32**) by the 19-hydroxylation of isovaleryl group [36]. 19-Hydroxy T-2 toxin (**32**) has been found to be more toxic than T-2 toxin (**31**). Therefore, it will be more toxic to the body when T-2 toxin (**31**) is transformed to 19-OH T-2 toxin (**32**) in humans and animals [37].

Zearalenone (ZEN, **33**) was converted into (5*S*)-5-hydroxy ZEN (**34**) with hydroxylation of the aliphatic ring of ZEN (**33**) by the fungus *Cunninghamella bainieri* (Figure S13) [38]. By using reference compounds and ZEN (**33**) labeled with deuterium at specific positions, evidence was provided for the preferential hydroxylation of ZEN (**33**) at C-8 and, to a lesser extent, at C-5, C-9 and C-10 with rat liver microsomes. The stereochemistry of the aliphatic hydroxylation products of ZEN (**33**) needs to be further studied [39]. In addition, ZEN (**33**) was converted into two monohydroxylated metabolites namely 13-hydroxy-ZEN (**35**) and 15-hydroxy-ZEN (**36**) with aromatic hydroxylation by human hepatic (liver) microsomes (Figure S14) [40].

### 2.2. Oxido-Reduction between Alcohols and Ketones

Oxido-reduction between alcohols and ketones of mycotoxins include: (i) oxidation of the hydroxyl group and (ii) reduction of the carbonyl group (Table 2).

Aflatoxin B_1_ (AFB_1_, **1**) was converted into alflatoxicol (**37**) through reduction of the keto group to the hydroxyl group by several fungi such as *Eurotium herbariorum*, *Rhizopus* sp. and non-aflatoxin-producing *Aspergillus flavus* (Figure S15) [41].

A dehydrogenase responsible for the selective oxidation of deoxynivalenol (DON, **38**) at C-3 position by converting DON (**38**) to 3-keto-DON (**39**) was revealed from the bacterium *Devosia* sp. (Figure S16) [42].

Fomannoxin (**40**) is a benzohydrofuran phytotoxin that was produced by the forest pathogen *Heterobasidion annosum* during the infection process [43]. When fomannoxin (**40**) was added in the cell cultures of *Pinus sylvestris*, the phytotoxin was transformed into the non-toxic formannoxin alcohol (**41**) (Figure S17) [44].

Zearalonone (ZEN, **33**) was transformed to both α-zearalenol (α-ZEL, **42**) and β-zearalenol (β-ZEL, **43**) with reduction by the fungi *Candida tropicalis* (Figure S18) [45], *Saccharomyces cerevisae* [46], *Torulaspora delbruckii* [46] and *Zygosaccharomyces rouxii* [46]. In contrast, only α-ZEL (**42**) was transformed from ZEA by *Pichia fermentans* and several yeast strains of the genera *Candida*, *Hansenula*, *Brettanomyces*, *Schizosaccharornyces* and *Saccharomycopsis* [46], as well as *Rhizopus* sp. and *Aspergillus* sp. [47].

### 2.3. Hydrogenation of the Carbon-Carbon Double Bond

Hydrogenation of the carbon-carbon double bond of mycotoxins means the reduction of a C-C double bond through biotransformation (Table 3). Aflatoxin B_1_ (AFB_1_, **1**) was converted to AFB_2_ (**4**) with hydrogenation by the fungus *Penicillium raistrickii* (Figure S19) [14]. Zearalenone (ZEN, **33**) was transformed to zearalanone (ZAN, **44**) with reduction of the carbon-carbon bond in ovine (Figure S20) [49].

### 2.4. De-Epoxidation

De-epoxidation of mycotoxins is commonly found in trichothecene analogues (Table 4) [50,51]. Trichothecene toxicity depends heavily upon the epoxide moiety of the molecule, and opening the epoxide ring dramatically reduces the toxicity [52].

Deoxynivalenol (DON, **38**) was converted to deepoxydeoxynivalenol (DOM, **45**) with de-epoxidation by *Eubacterium* sp. DSM11798 (Figure S21) [50].

Nivalenol (NIV, **46**) belongs to group B trichothecenes. NIV (**46**) was converted to de-epoxynivalenol (de-epoxy-NIV, **47**) with de-epoxidation by the bacterium *Eubacterium* sp. BBSH 797 (Figure S22) [51]. Similarly, after the male Wistar rats were orally administered with NIV (**46**), a metabolite was isolated from rat feces and identified as de-epoxy-NIV (**47**), namely 3,4,7,15-tetrahydroxytrichothec-8,12-dien-8-one (Figure S22) [53].

### 2.5. Other Oxido-Reductions

Other oxido-reductions of mycotoxins include epoxidation, oxidation of alcohols to acids, reduction of acids to alcohols, and multi-step oxido-reductions (Table 5). Aflatoxin B_1_ (AFB_1_, **1**) was catalyzed into AFB_1_-8,9-epoxide (**48**) by channel catfish liver microsomes (Figure S23) [20]. AFB_1_ (**1**) was also transformed to AFB_1_-8,9-dihydrodiol (**49**) by the fungus *Phanerochaete sordia* (Figure S24) [54].

Two *Alternaria* toxins with a perylene quinone structure, altertoxin II (ATX II, **50**) and stemphyltoxin III (STTX III, **51**) were reduced to alcohols in human colon Caco-2 cells, resulting in the formation of altertoxin I (ATX I, **52**) and alteichin (ALTCH, **53**), respectively (Figure S25). ATX II (**50**) was also reduced to ATX I (**52**) in other human tumor cell lines such as HCT 116, HepG2 and V79 [55].

Botrydial (**54**) was converted to dihydrobotrydial (**55**) and secobotrytrienediol (**56**) with a few oxido-reductions by the fungus *Botrytis cinerea* (Figure S26) [56].

Citrinin (CTN/CIT, **57**) is a polyketide nephrotoxic mycotoxin commonly present as a natural hazardous contaminant both in food and feed world wide. It was first isolated from the fungus *Penicillium citrinum* [8]. Dihydrocitrinone (DH-CTN, **58**) was detected as the main metabolite of CTN (**57**) (Figure S27) in the urine of rats [57] and humans [58]. CTN (**57**) induced a concentration-dependent increase in micronucleus frequencies at concentrations ≥30 μM, wheas DH-CTN (**58**) showed no genotoxic effect up to 300 μM. Thus, conversion of CTN (**57**) to DH-CTN (**58**) in humans can be regarded as a detoxification step [58].

Fomannoxin (**40**) is a phytotoxic dihydrobenzofuran produced by the fungi from genus *Heterobasidion* (*Fomes*) [43]. Fomannoxin (**40**) was added to the cultures of rhizosphere-associated *Streptomyces* sp. AcH 505, it was converted into different products by oxido-reduction, most of products retained phytotoxic activity. These products included fomannoxin alcohol (**41**), fomannoxin acid (**59**), fomannoxin amide (**60**), 2,3-dihydro-2-(2′-hydroxyisopropanyl)-5-benzofurancarboxylic acid (MFA-1, **61**), 2,3-dihydro-3-hydroxyl-2-isopropenyl-5-benzofurancarboxylic acid or 3-hydroxyl-fomannoxin acid (MFA-2, **62**) and 2,3-dihydro-2-(1′,2′-dihydroxyisopropanyl)-5-benzofurancarboxylic acid (DFA, **63**) (Figure S28) [48].

Fusaric acid (FA, **19**) was reduced to fusarinol (**64**), also called 5-butyl-2-pyridinemethanol, by the fungus *Aspergillus tubingensis* (Figure S29) [59]. Fusarinol (**64**) was significantly less phytotoxic than fusaric acid (**19**). In fusarinol (**64**), the acid has been reduced to an alcohol group. The reduced phytotoxicity of fusarinol (**64**) indicates the importance of the carboxylic acid in the toxic function of FA (**19**). The fungus *A. tubingensis* provides a novel detoxification mechanism against FA (**19**), which may be utilized to control *Fusarium* wilt [59].

The marine yeast *Kodameae ohmeri* was found to transform patulin (PAT, **65**) into *E*-ascladiol (**66**) and *Z*-ascladiol (**67**) through reduction (Figure S30). High transformation rate was at a temperature of 35 °C and pH between 3 and 6 that indicated the potential application of *K. ohmeri* for PAT (**65**) detoxification of the contaminated products [60]. *E*-ascladiol (**66**) and *Z*-ascladiol (**67**) have been found to exhibit no signs of toxicity towards human cell lines derived from the intestinal tract, kidney, liver and immune system, which demonstrates that PAT (**65**) detoxification strategies leading to the accumulation of ascladiols should be approaches to limit the PAT (**65**) risk [61]. When PAT (**65**) was added in the cell cultures or cell-free supernatant of *Lactobacillus plantarum*, it was transformed to *E*-ascladiol (**66**) and *Z*-ascladiol (**67**), which were further transformed into hydroascladiol (**68**) over a 4-week cell-free incubation at 4 °C (Figure S31) [62].

### 2.6. Methylation

Methylation of myctoxins was observed on the hydroxyl groups. The transformation was catalyzed by *O*-methyltransferase (Table 6).

Alternariol (AOH, **7**) was converted into alternariol 9-*O*-methyl ether (AME, **8**) by methylation through an alternariol *O*-methyltranferase. The methyl donor was *S*-adenosyl-L-methionine (SAM) (Figure S32) [63].

Zearalenone (ZEN, **33**) was converted into zearalenone 16-methyl ether (**69**) and zearalenone 14,16-bis (methyl ether) (**70**) by the fungus *Cunninghamella bainieri* (Figure S33). Zearalenone 16-methyl ether (**69**) showed similar estrogenic activity compared with the parent compound ZEN (**33**). However, zearalenone 14,16-bis (methyl ether) (**70**) exhibited inactive estrogenic effect [38].

### 2.7. Glycosylation and Glucuronidation

Glycosylation/glucuronidation of mycotoxins is the process by which a glucose or glucuronic acid is covalently attached to a hydroxyl group (Table 7). Glycosylation/glucuronidation often increases the polarity of mycotoxins, and reduce their toxicity. Many glycosyltransferases of mycotoxins are present in plants. A UDP-glucosyltransferase involved in the detoxification of deoxynivalenol was revealed from rice (*Oryza sativa*) [65].

Alternariol (AOH, **7**) could be glycosylated into 3-*O*-β-d-glucopyranosyl AOH (**71**), 9-*O*-β-d-glucopyranosyl AOH (**72**), 9-*O*-β-d-glucopyranosyl (1→6)-β-d-glucopyranosyl AOH (**73**) by suspension cultured cells of *Nicotiana tabacum* (Figure S34) [66]. The suspension cultured cells of *N. tabacum* had also the ability to transform alternariol 9-*O*-methyl ether (AME, **8**) into 3-*O*-β-d-glucopyranosyl AME (**74**) and 7-*O*-β-d-glucopyranosyl AME (**75**) through glycosylation (Figure S35) [66]. In addition, both AOH (**7**) and AME (**8**) were metabolized to their corresponding sulfate conjugates in cultures of *Alternaria alternata*. The sulfates such as AOH 3-sulfate (**76**), AOH 9-sulfate (**77**) and AME 3-sulfate (7**8**) were subsequently conjugated to their sulfoglucosides AOH 3-sulfate-9-*O*-glucoside (**79**), AOH 9-sulfate-3-*O*-glucoside (**80**) and AME 3-sulfate-7-*O*-glucoside (**81**) in either tomato tissues or tobacco cultured cells (Figure S36) [67].

Curvularin (**82**) is a macrocyclic lactone produced by a number of fungi from the genera *Curvularia*, *Penicillium* and *Alternaria*, and have been reported to possess a variety of biological activities including phytotoxicity [68]. Curvularin (**82**) was converted into curvularin 11-*O*-β-d-glucopyranoside (**83**) and curvularin 4′-*O*-methyl-11-*O*-β-d-glucopyranoside (**84**) with glycosylation and methylglycosylation through the the fungus *Beauveria bassiana* (Figure S37) [69].

Deoxynivalenol (DON, **38**) is the main trichothecene toxin produced by the *Fusarium* species and a relevant virulence factor in *Fusarium* head blight (FHB) disease of cereal crops. Trichothecene toxins inhibited eukaryotic protein synthesis and elicited a wide range of pathophysiological effects in humans and animals. DON (**38**) could be glycosylated to DON 3-*O*-β-d-glucoside (D3G, **85**), which was a masked mycotoxin, by a recombinant UDP-glucosyltranferase from rice (Figure S38) [70]. DON (**38**), deepoxy-deoxynivalenol (DOM, **45**), iso-deoxynivalenol (iso-DON, **86**) and iso-deepoxy-deoxynivalenol (iso-DOM, **87**) were respectively transformed to a series of glucuronides (**88**–**97**) by glucuronidation through rat liver microsomes (RLM) or human liver microsomes (HLM) (Figures S39–S42) [71,72].

Both 15-monoacetoxyscirpenol (15-MAS, **98**) and 4,15-diacetoxyscirpenol (4,15-DAS, **99**) were produced by the fungi from the genus *Fusarium* such as *F. sporotrichioides* and *F. poae* [73]. In corn plants, both 15-MAS (**98**) and 4,15-DAS (**99**) were, respectively, transformed to 15-MAS 3-glucoside (**100**) and 4,15-DAS 3-glucoside (**101**), which were called masked mycotoxins. The structures of transformed products (**99** and **100**) were deduced on the basis of accurate mass measurements of characteristic ions and fragmentation patterns by using high-resolution liquid chromatography–Orbitrap mass spectrometric analysis. Although their absolute structures were not clarified, 3-OH glucosylation appeared to be the most probable (Figure S43) [74,75]. As the authors used *Fusarium* sp. infected corn material, it is not clear whether the corn plant or the fungus itself produced the glucosides just like formation of MAS glucoside [76] as well as the glucosides of HT-2 and MAS [77] produced by *Fusarium* species.

4,15-Diacetoxyscirpenol (4,15-DAS, **99**) was transformed to 4,15-DAS 3-glucuronide (**102**) with glucuronidation in rats (Figure S44) [78].

Hydroxydestruxin B (**103**) was glycosylated into glucosyl hydroxydestruxin B (**104**) in cruciferous plants such as *Brassica napus* (Figure S45) [24]. Fungal phytotoxin detoxification should be a resistance mechanism of crucifers against pathogens [25].

T-2 toxin (**31**) was converted into T-2 toxin 3-*O*-α-d-glucoside (**105**) with glycosylation by the fungi *Blastobotrys muscicola* and *B. robertii* (Figure S46) [79]. In animal tissures, T-2 toxin (**31**) was transformed to T-2 toxin-3-*O*-β-d-glucuronide (T-2 GlcA, **106**) by UDP glucuronyl transferase (UDPGT) released from rat liver microsomes (Figure S47) [80].

Zearalenone (ZEN, **33**) was glycosylated into zearalenone 14-*O*-glucoside (ZEN 14-*O*-Glc, **107**) by *Arabidopsis* UDP-glucosyltransferases expressed in *Saccharomyces cerevisiae* (Figure S48) [81]. Similarly, ZEN (**33**) was converted to ZEN 14-*O*-Glc (**107**) with glycosylation by the fungi *Mucor vainieri* and *Thamnidium elegans* [82]. ZEN (**33**) was simultaneously glycosylated into ZEN 14-*O*-Glc (**107**) and zearalenone 16-*O*-glucoside (ZEN 16-*O*-Glc, **108**) by a barley UDP-glucosyltransferase expressed in *Saccharomyces cerevisiae* (Figure S49) [83]. The monoglucosides (i.e., ZEN 14-*O*-Glc and ZEN 16-*O*-Glc) could be further transformed to ZEN 14,16-di-glucoside (**109**) by the recombinant barley glucosyltransferase (Figure S50) [84].

### 2.8. Esterification

Esterification of mycotoxins is a reaction to form ester mainly from alcohols and carboxylic acids (Table 8). Ochratoxin A (OTA, **21**) was converted to OTA methyl ester (**110**) by the cell cultures of wheat and maize (Figure S51) [31].

T-2 toxin (**31**) was converted to 3-acetyl T-2 toxin (**111**) with acetylation by bovine rumen fluid *in vitro* (Figure S52) [85].

### 2.9. Hydrolysis

Both ester and amide bonds of mycotoxins were widely investigated for hydrolysis (Table 9) [79,80,86,87]. A crude cell-free extract from cultures of *Fusairum* sp. strain C37410-90 possessed significant esterase activity and hydrolyzed the 3-acetyl deoxynivalenol (3-acetyl DON, **112**) to DON (**38**) (Figure S53) [88]. This de-acetylation was also found in the fungus *Sphaerodes mycoparasitica* incubated with 3-acetyl DON (**112**) [87].

4,15-Diacetoxyscirpenol (4,15-DAS, **99**) is a potent mycotoxin produced by some *Fusarium* species. 4,15-DAS (**99**) was hydrolyzed to either 4-monoacetoxyscirpenol (4-MAS, **113**) or 15-monoacetoxyscirpenol (15-MAS, **98**), and the final product was scirpentriol (SCP, **114**) with deacetylation in rats (Figure S54) [78].

Fumunisin B_1_ (FB_1_, **115**) was transformed to the hydrolyzed FB_1_ namely aminopentol 1 (AP1, **116**), with hydrolysis by the fungus *Exophiala spinifera* 2141.10 (Figure S55) [89]. FB_1_ (**115**) was also converted to AP1 (**116**) either with the enzyme from the bacterium *Sphingopyxis* sp. MTA144 [90] or with carboxylesterase FumD [91].

Fusarenon-X (FX, **117**) was hydrolyzed into nivalenol (NIV, **46**) via deacetylation in mice [92] and goat (*Capra hircus*) [93] excreted mainly in urine (Figure S56). The liver and kidney are the organs responsible for the conversion of FX (**117**) to NIV (**46**).

The crude lipase from *Aspergillus niger* was screened to degrade ochratoxin A (OTA, **21**) to nontoxic OTα (**118**) and L-β-phenylalanine (**119**) (Figure S57) among 23 commercial hydrolases [94]. Other active enzymes having the same conversion included protease A, prolyve PAC and pancreatin [95], as well as carboxypeptidase A [96]. In addition, OTA (**21**) was found to be hydrolyzed to OTα (**118**) and L-β-phenylalanine (**119**) by *Aspergillus niger* [97] and *Bacillus amyloliquefaciens* [98].

OTA (**21**) was converted into a lactone-opened ochratoxin A (OP-OTA, **120**), which was named *N*-[3-carboxy-5-chloro-2-hydroxy-4-(2-hydroxypropyl) benzoyl]-L-phenylalanine (Figure S58) [99]. OP-OTA (**120**) was tested to be more toxic than the parent molecule which indicated that the lactone ring was not considered to be responsible for the toxic activity of OTA (**21**) [16].

Ochratoxin C (OTC, **121**), which was also called OTA ethyl ester, was hydrolyzed into OTA (**21**) by deacetylation in rats after oral and intravenous administration (Figure S59). The very fast conversion of OTC (**121**) into OTA (**21**) is a possible explanation of the similar toxicity of ocratoxins A (**21**) and C (**121**) to animals and humans [100].

T-2 toxin (**31**) was selectively hydrolyzed at C-4 by the bacterium *Eubacterium* BBSH 797, giving rise to HT-2 toxin (**122**) as the only metabolite (Figure S60) [86]. The previous study on the metabolism of T-2 toxin (**31**) in livers of rabbits, rats, guinea pigs and mice also showed that T-2 toxin (**31**) was only hydrolyzed at C-4 to HT-2 toxin (**122**) by deacetylation [101].

In the rat liver and intestines, T-2 toxin (**31**) was first hydrolyzed into HT-2 toxin (**122**), which was further converted to 15-acety-tetraol (**123**), which was finally transformed to T-2 tetraol (**124**) with hydrolyzation (Figure S61) [102].

In addition, T-2 toxin (**31**) was selectively hydrolyzed at C-8 resulting in the removal of isovaleryl group by the fungus *Blastobotrys capitulate* to neosolaniol (**125**) (Figure S62) [79].

### 2.10. Sulfation

Sulfation of mycotoxins is the sulfotransferase-catalyzed conjugation of a sulfo group on a hydroxyl group (Table 10). Both deoxynivalenol (DON, **38**) and zearalenone (ZEN, **33**) were converted to their corresponding sulfates DON 3-sulfate (**126**) and ZEN 14-sulfate (**127**) (Figure S63) by the fungus *Sphaerodes mycoparasitica* [87]. ZEN (**33**) was also found to be converted to ZEN 14-sulfate (**127**) by pigs [103]. Both DON 3-sulfate (**126**) and ZEN 14-sulfate (**127**) were less toxic than DON (**38**) and ZEN (**33**), respectively [87].

### 2.11. Demethylation

Demethylation of mycotoxins is the process resulting in the removal of a methyl group from a molecule (Table 11). A common way of mycotoxin demethylation is replacement of a methoxyl group by a hydroxyl group. Alternariol 9-*O*-methyl ether (AME, **8**) was converted to alternariol (AOH, **7**) with demethylation by the homogenate of porcine liver in the presence of NADPH (Figure S64) [104].

Aflatoxin B_1_ (AFB_1_, **1**) was converted to aflatoxin P_1_ (AFP_1_, **128**) with demethylation by the enzyme CYP321A1 from corn earworm *Helicoverpa zea* (Figure S65). AFP_1_ (**128**) was more polar and less toxic than AFB_1_ (**1**) [105].

### 2.12. Deamination

Deamination of mycotoxins is the removal of an amino group from a molecule catalyzed by the deaminase (Table 12). Fumonisin B_4_ (FB_4_, **129**) was transformed into fumonisins La_4_ (Fla_4_, **130**) and Py_4_ (FPy_4_, **131**) through oxidative deamination by *Aspergillus* sp. (Figure S66) [106]. Using a duckweed (*Lemna minor*) bioassay, both Fla_4_ (**130**) and FPy_4_, (**131**) were significantly less toxic in comparision to the fumonisin B mycotoxins. This demonstrated that *Aspergillus* fungi have the ability to produce enzymes that could be used for fumonisin detoxification [106].

Hydrolyzed fumonisin B_1_ (HFB_1_, **116**), which was also named as aminopentol 1 (AP1), was converted into 2-keto HFB_1_ (**132**) or namely 2-keto AP1 through oxidative deamination by the fungus *Exophiala spinifera* (Figure S67) [107].

### 2.13. Miscellaneous Reactions

Many other types of biotransformation of mycotoxins have also been reported, such as epimerization, epoxidation, dehydrogenation, dechlorination and their multi-step conversions (Table 13).

Aflatoxin B_1_ (AFB_1_, **1**) was converted to dihydrohydroxyaflatoxin B_1_ (**133**), which was also named as aflatoxin B_2a_ (AFB_2a_), via reduction and oxidation by *Pleurotus ostreatus* GHBBF10 (Figure S68) [108].

AFB_1_ (**1**) was detoxified to aflatoxin D_1_ (AFD_1_, **134**), aflatoxin D_2_ (AFD_2_, **135**) and aflatoxin D_3_ (AFD_3_, **136**) through hydrolysis, decarboxylation, and oxidation-reduction by *Pseudomonas putida*, which was isolated from sugarcane (Figure S69). The conversion mechanism from AFB_1_ (**1**) to AFD_1_ (**134**) was also entirely elucidated [109]. Cytotoxicity study clearly implied that AFD_1_ (**134**) was non-toxic, and both AFD_2_ (**135**) and AFD_3_ (**136**) were much less toxic by comparing with AFB_1_ (**1**) [110].

Alternariol (AOH, **7**) was transformed to 3-*O*-(6-*O*-malonyl-β-d-glucopyranosyl) AOH (**137**) and 9-*O*-(6-*O*-malonyl-β-d-glucopyranosyl) AOH (**138**) through glycosylation and esterification by the cell cultures of *Nicotiana tabacum* (Figure S70) [66]. Similarly, alternariol 9-*O*-methyl ether (AME, **8**) was also transformed to 3-*O*-(4-*O*-malonyl-β-d-glucopyranosyl) AME (**139**) and 3-*O*-(6-*O*-malonyl-β-d-glucopyranosyl) AME (**140**) with glycosylation and esterificationby by the suspension cell cultures of *Nicotiana tabacum* (Figure S71) [66]

Botrydial (**54**) was converted into botryenedial (**141**) with dehydration by the fungus *Botrytis cinerea* (Figure S72). Botryenedial (**141**) was less phytotoxic than botrydial (**54**) [56].

Citrinin (CTN, **57**) was first identified as an antibiotic. It was also considered as a mycotoxin to damage DNA. CTN (**57**) was converted to decarboxycitrinin (**142**) with decarboxylation by the bacterium *Moraxella* sp. MB1 (Figure S73) [111]. Decarboxycitrinin (**142**) was also the product of heat treatment of CTN (**57**), and retained antibiotic activity, but was not toxic to mice [112].

Deoxynivalenol (DON, **38**) was converted into 3-*epi*-deoxynivalenol (**143**) (Figure S74) with epimerization by *Nocardioides* sp. WSN05-2 [113], and *Devosia* sp. [114]. It was known that DON (**38**) epimerization proceeded through the formation of 3-keto-DON (**39**) intermediate to convert to 3-*epi*-deoxynivalenol (**143**) by a two-step biocatalysis [115].

When deoxynivalenol (DON, **38**) was incubated with rat liver microsomes (RLM), the isomer iso-DON (**86**) was formed with isomerization [71]. Iso-DON (**86**), which was less toxic than DON (**38**), was also found to be converted from DON (**38**) under harsh condition non-enzymatically [116]. Other transformed products included iso-DON-3-GlcA (**92**), iso-DON-8-GlcA (**93**) and DON-8,15-hemiketal-8-GlcA (**144**) (Figures S75 and S76) [71]. When deepoxy-deoxynivalenol (DOM, **45**) was incubated with RLM, iso-DOM (**87**), iso-DOM-3-GlcA (**95**) and iso-DOM-8-GlcA (**96**) were transformed with isomerization and glucuronization (Figure S77) [71].

When fomannoxin (**40**) was added in the cell cultures of *Pinus sylvestris*, formannoxin (**40**) was transformed into the non-toxic fomannoxin acid (**59**), which was further glycosylated to fomannoxin acid β-glucoside (**145**) with time prolonging (Figure S78) [44].

Degradation transformation of fumonisin B_1_ (**115**) comprises at least two enzymatic steps, an initial hydrolysis (deesterification reaction) to form aminopentol 1 (AP1, **116**), followed by deamination of AP1 (**116**) to form 2-keto AP1 (**132**). Two recombinant enzymes carboxylesterase and aminotransferase from the bacterium *Sphingopyxis* sp. were expressed heterologously. Carboxylesterase catalyzed fumonisin B_1_ (**115**) to AP1 (**116**) with deesterification (Figure S79). Aminotransferase catalyzed AP1 (**116**) to 2-keto AP1 (**132**) with deamination in the presence of pyruvate and pyridoxal phosphate [90].

Ochratoxin (OTA, **21**) was hydroxylated into (4*R*)-4-hydroxyochratoxin A (**24**) and (4*S*)-4-hydroxyochratoxin A (**25**), which were further glycosylated and esterified, respectively, by the cell cultures of wheat and maize to (4*R*)-4-hydroxyochratoxin A methyl ester (**146**), (4*S*)-4-hydroxyochratoxin A methyl ester (**147**), (4*R*)-4-hydroxyochratoxin A 4-*O*-β-d-glucoside (**148**) and (4*S*)-4-hydroxyochratoxin A 4-*O*-β-d-glucoside (**149**) (Figure S80) [31]. OTA (**21**) was dechlorinated into ochratoxin B (OTB, **27**) in the renal microsomes of rabbits pretreated with phenobarbital (PB). OTB (**27**) was less toxic than OTA (**21**) (Figure S81) [117].

Patulin (PAT, **65**) was transformed to desoxypatulinic acid (DPA, **150**) with multi-step reactions (Figure S82) by the fungi *Rhodotorula kratochvilovae* [118] and *Rhodosporidium paludigenum* [119]. Conversion of PAT (**65**) to the less toxic desoxypatulinic acid (**150**) was considered a detoxification process [120].

Zearalenone (ZEN, **33**) was transformed to α-zearalenol (**42**), β-zearalenol (**43**), α-zearalanol (**151**), β-zearalanol (**152**) and zearalanone (**44**) with multi-step oxido-reductions in human body (Figure S83) [121]. In addition, ZEN (**33**) was converted to hydrolyzed ZEN (**155**) through hydrolysis, and then to decarboxylated hydrolyzed ZEN (**156**) through spontaneous decarboxylation by *Bacillus pumilus* (Figure S84) [122]. Similar biotransformation of ZEN (**33**) was also observed by using a lactonase named ZHD101 from *Clonostachys rosea* [123,124].

## 3. Detoxification of Important Mycotoxins by Biotransformation

### 3.1. Detoxification of Aflatoxins

Aflatoxins (AFs) are a group of furanocoumarin mycotoxins mainly produced by *Aspergillus* species. They are threats to human and animal health, and are classified by the International Agency for Research on Cancer (IARC) as group 1 carcinogens [7,14,125,126]. The furofuran ring of AFs has been recognized as responsible for the toxic and carcinogenic activity upon metabolic activation of the C8-C9 double bond to 8-9 epoxide [7]. The epoxidation is a crucial reaction for carcinogenicity of AFs, since it allows the binding to N7-guanine and the subsequent G to T transversions in the DNA molecule [127]. Activated AFs are also able to form Schiff bases with cellular and microsomal proteins (via methionine, histidine and lysine), thus leading to acute toxicity [128]. The lactone ring also plays a role in AFs toxicity and carcinogenicity. DNA alkylation depends upon both difuranocumarin and lactone moieties of AFs [129].

Many studies have focused on the detoxification of aflatoxins [130]. However, only a few studies detected the converted products and analyzed their toxicity. Two main detoxification pathways, which are modifications of the difuran ring and coumarin structure of AFs, have been investigated. Some reviews on the detoxification of aflatoxins through biotransformation have been available [12,131,132,133,134].

Among the isolated AFs, aflatoxin B_1_ (AFB_1_, **1**) is not only the compound with the highest content, but also the most toxic. It is responsible for liver cancer in animals. Microbial degradation of AFB_1_ (**1**) has been well reviewed [130]. For the AFB_1_ (**1**), biotransformation pathways mainly include epoxidation of the carbon-carbon double bond of the furan ring, hydroxylation of the difuran ring, demethylation of methoxy group of aromatic ring, reduction of carbonyl group of cyclopentenone and C3 hydroxylation of cyclopentenone [131,134]. Among them, the reduction of carbonyl group in pantan cycle to hydroxyl group by several fungal species (Figure S15) [41], and multi-step oxidation-reduction reactions of AFB_1_ (**1**) to AFD_1_ (**134**), AFD_2_ (**135**) and AFD_3_ (**136**) by *Pseudomonas putida* (Figure S69) [109] were effective reactions for detoxification. The main transformation pathways of AFB_1_ (**1**) are as follows.

(i) Reduction of the carbonyl group: the carbonyl group in pantan cylcle of AFB_1_ (**1**) was reduced to hydroxyl group. AFB_1_ (**1**) was converted into alflatoxicol (**37**) by several fungi [41]. Aflatoxicol (**37**) was 18 times less toxic than AFB_1_ (**1**) [130].

(ii) Epoxidation: AFB_1_ (**1**) was catalyzed into AFB_1_-8,9-epoxide (**48**), which was more toxic than AFB_1_ (**1**) [130].

(iii) Hydroxylation: AFB_1_ (**1**) was hydroxylated to AFM_1_ (**2**) and AFQ_1_ (**3**) by hepatic microsomal mixed-function oxidase from the rhesus monkey [22]. AFB_1_ (**1**) was catalyzed to AFQ_1_ (**3**) by rat liver microsomal cytochrome P450p. Furthermore, treatment of male rats with pregnenolone-16α-carbonitrile resulted in a 16-fold increase in the formation of AFQ_1_ (**3**) [21].

(iv) Reduction and oxidation: AFB_1_ (**1**) was converted to AFB_2a_ (**133**), which was also called dihydrohydroxyaflatoxin B_1_, by *Pleurotus ostreatus* GHBBF10. AFB_2a_ (**133**) was 200 times less toxic than AFB_1_ (**1**) [108].

(v) Demethylation: AFB_1_ (**1**) was converted to AFP_1_ (**128**) with demethylation by the enzyme CYP321A1 from corn earworm *Helicoverpa zea*. AFP_1_ (**128**) was more polar and less toxic than AFB_1_ (**1**) [105].

(vi) Multi-step reactions including hydrolysis, decarboxylation and oxidation-reduction: AFB_1_ (**1**) was transformed to structurally different AFD_1_ (**134**), AFD_2_ (**135**) and AFD_3_ (**136**) with hydrolysis, decarboxylation and oxidation-reduction by *Pseudomonas putida*. The converted products were nontoxic or much less toxic than AFB_1_ (**1**) towards HeLa cells [109,110].

### 3.2. Detoxification of Alternaria Toxins

*Alternaria* species produce several groups of mycotoxins to cause plant diseases as well as to pose human and animal health problem. *Alternaria* mycotoxins mainly include alternariol (AOH, **7**), alternariol 9-methyl ether (AME, **8**), altenuisol, altenuene, tenuazonic acid, altertoxin II (**50**), stemphyltoxin III (**51**), tentoxin and AAL-toxins. Both AOH (**7**) and AME (**8**), which belong to dibenzo-α-pyranones, are main mycotoxins [135]. They have been considered to cause esophageal cancer [136], as well as to exhibit mutagenicity and genotoxicity [137,138]. The main detoxification reactions of *Alternaria* toxins were glycosylation and glucuronidation by plants [66,67]. The transformation pathways of *Alternaria* toxins are as follows.

(i) Glycosylation: AOH (**7**) could be glycosylated into 3-*O*-β-glucopyranosyl AOH (**71**), 9-*O*-β-glucopyranosyl AOH (**72**), 9-*O*-β-glucopyranosyl (1→6)-β-d-glucopyranosyl AOH (**73**) by the suspension cultured cells of *Nicotiana tabacum* [66]. The suspension cultured cells of *N. tabacum* had also the ability to transform AME (**8**) into 3-*O*-β-d-glucopyranosyl AME (**74**) and 7-*O*-β-d-glucopyranosyl AME (**75**) through glycosylation [66]. Both the glucosides of AOH (**7**) and AME (**8**) could be further esterified into the malonyl acylation products by *N. tabacum* cultured cells [66]. Furthermore, the sulfate conjugates of AOH (**7**) and AME (**8**) were converted into their sulfoglucosides such as AOH 3-sulfate-9-*O*-glucosie (**79**), AOH 9-sulfate-3-*O*-glucoside (**80**) and AME 3-sulfate-7-*O*-glucoside (**81**) by tobacco suspension cells or ex planta tomato tissues [67].

(ii) Hydroxylation: Both AOH (**7**) and AME (**8**) can be hydroxylated to their monohydroxy products by the microsomes from rat, human, and porcine liver [23].

(iii) Reduction: Both altertoxin II (**50**) and stemphyltoxin III (**51**) with a perylene quinone structure were reduced to alcohols in human colon Caco-2 cells, resulting in the formation of altertoxin I (**52**) and alteichin (**53**), respectively. Altertoxin II (**50**) was also reduced to alcohol in other human cell lines such as HCT 116, HepG2 and V79 [55].

(iv) Methylation: AOH (**7**) was converted into AME (**8**) through an alternariol *O*-methyltranferase [63].

(v) Demethylation: AME (**8**) was demethylated into AOH (**7**) by the homogenate of porcine liver in the presence of NADPH [104].

### 3.3. Detoxification of Citrinin

Citrinin (CTN/CIT, **57**) is a mycotoxin produced by the fungi from genera *Monascus*, *Aspergillus* and *Penicillium*, which contaminate plant seeds and other foods. CTN (**57**) is known as a hepata-nephrotoxinc mycotoxin with immunotoxic and carcinogenic properties [8]. The main transformation pathways of CTN (**57**) are as follows.

(i) Decarboxylation: CTN (**57**) was converted to decarboxycitrinin (**142**) by the bacterium *Moraxella* sp. MB1 [111].

(ii) Oxido-reduction: CTN (**57**) was transformed to dihydrocitrinone (**58**) in the urine of rats [57] and humans [58].

### 3.4. Detoxification of Fomannoxin

Fomannoxin (**40**) is a fungal benzohydrofuran aldehyde phytotoxin, which was produced by the forest pathogenic basidiomycete *Heterobasidion annosum* during the infection process [43]. Fomannoxin (**40**) showed growth-inhibiting effects on callus and suspension cultures of conifer cells [44]. The most effective detoxification for fomannoxin (**40**) was reduction by the cultured cells of *Pinus sylvestris* (Figure S17) [44]. The main transformation pathways of fomannoxin (**40**) are as follows.

(i) Reduction: when fomannoxin (**40**) was added in the cell cultures of *Pinus sylvestris*, formannoxin was transformed into the non-toxic formannoxin alcohol (**41**) [44].

(ii) Reduction and esterification: the transformed formannoxin alcohol (**41**) from fomannoxin (**40**) by the cell cultures of *P. sylvestris* was further converted to fomannoxin acid β-glucoside (**145**) with time prolonging [44].

(iii) Oxido-reduction: fomannoxin (**40**) was converted into different products such as fomannoxin alcohol (**41**), fomannoxinn acid (**60**), MFA-1 (**61**), MFA-2 (**62**), and DFA (**63**) through oxido-reduction by rhizosphere-associated *Streptomyces* sp. AcH 505. Most of the products retained phytotoxic activity [48].

### 3.5. Detoxification of Fumonisins

Fumonisins are mainly produced by *Fusarium verticillinoids* and *F. proliferatum*, and are structurally similar to sphingolipid long-chain bases such as sphinganine and sphingosine [17]. Fumonisin B_1_ (FB_1_, **115**) is the most prevalent fumonisin and holds the highest risk for human and animal nutrition among FBs. They contaminate corn and its processed foods [139]. This feature is tightly related to their toxicity mechanism through the inhibition of the sphingolipid biosynthesis in animals, plants, and yeasts [140,141]. A wide variety of diseases in animals such as liver cancer in rats, equine leukoencephalomalacia and porcine pulmonary edema have been associated with fumonisins [142]. They may cause neural tube defects in some maize-consuming populations [143].

Detoxification of fumonisins through biotransformation mainly include hydrolysis with loss of two tricarballylic side-chains by carboxylesterase, and deamination by aminotransferase [90]. The main transformation pathways of fumonisins are as follows.

(i) Hydrolysis: FB_1_ (**115**) was hydrolyzed into aminopentol 1 (AP1, **116**) with deesterification reaction by the fungus *Exophiala spinifera* 2141.10 [89]. FB_1_ (**115**) was also catalyzed by the recombinant carboxylesterase from the bacterium *Sphingopyxis* sp. [90].

(ii) Hydrolysis and deamination: FB_1_ (**115**) was hydrolyzed by carboxylesterase with loss of two tricarballylic acid (TCA) groups, followed by deamination by aminotransferase in the presence of pyruvate and pyridoxal phosphate [90].

(iii) Oxidative deamination: fumonisn B_4_ (FB_4_, **129**) was transformed into fumonisins La_4_ (Fla_4_, **130**) and Py_4_ (FPy_4_, **131**) through oxidative deamination by *Aspergillus* sp. Nonaminated Fla_4_ (**130**) and FPy_4_ (**131**) was significantly less toxic in comparison to the fumonisin B mycotoxins, which provided new insight into the mechanism of fumonisin toxicity [106].

### 3.6. Detoxification of Ochratoxins

Ochratoxins (OTs) are a group of mycotoxins as the carcinogenic substances to humans sharing an isocoumarin moiety substituted with: (i) a phenylalanine group including ochratoxin A (OTA, **21**), 10-hydroxyl-OTA (**26**), and OTB (**27**); (ii) a phenylalanine ester group including OTC (**121**), OTA methylester, OTB methyl ester and OTB ethyl ester; and (iii) a hydroxyl group including ochratoxin α (OTα, **118**) and OTβ. Among them, OTA (**21**) is the most hazardous mycotoxin which has been categorized as a carcinogenic of group 2B to humans [16]. Furthermore, OTA (**21**) showed a number of toxic effects to animals where the most prominent one was nephrotoxicity [144]. OTA (**21**) can be produced by various *Aspergillus* and *Penicillium* species. The most effective detoxification for OTA (**21**) was hydrolysis with transformed products as OTα (**118**) and phenylalanine (**119**) by several bacterial and fungal species (Figure S57) [94,95,96,97,98,145,146,147]. The main transformation pathways of OTs are as follows [16].

(i) Hydrolysis: hydrolysis of OTs include degradations of amide and ester bonds. OTA (**21**) could be hydrolyzed into OTα (**118**) and phenylalanine (Phe, **119**) by bacteria such as *Bacillus amyloliquefaciens* [98], *Alcaligenes faecalis* [145] and *Brevibacterium* sp. [146], as well as fungi such as *Aspergillus niger* [97] and *A. tubingensis* [147]. OTA (**21**) was further hydrolyzed into OTα (**118**) by carboxypeptidase produced by bacteria and fungi [94]. OTC (**121**) was also named as ochratoxin A ethyl ester which was converted into OTA (**21**) by deacetylation in rats after oral and intravenous administration [100]. OTA (**21**) was converted into a lactone-opened ochratoxin A (OP-OTA, **120**), which was named *N*-[3-carboxy-5-chloro-2-hydroxy-4-(2-hydroxypropyl) benzoyl]-L-phenylalanine [99].

(ii) Hydroxylation: hydroxylation of OTA (**21**) usually occurs at its C-4, C-5, C-8′, C-9′ and C-10 positons. Hydroxylation as an inactivation pathway in animal is catalyzed by CYP 450. OTA (**21**) was hydroxylated into 4*R*-hydroxyochratoxin A in the urine of rats after injection with OTA (**21**) [29]. OTA (**21**) was hydroxylated into 7-carboxy-(2′-hydroxy-1-phenylalanine-amide)-5-chloro-8-hydroxy-3,4-dihydro-3*R*-methylisocoumarin (**22**) and a dihydrodiol derivative (**23**) by a Gram-negative bacterium *Phenylobacterium immobile* [28].

(iii) Hydroxylation and glycosylation or esterification: OTA (**21**) was hydroxylated into (4*R*)-4-hydroxyochratoxin A (**24**) and (4*S*)-4-hydroxyochratoxin A (**25**), which were further glycosylated or esterificated by the cell cultures of wheat and maize [31].

(iv) Esterification: OTA (**21**) was catalyzed to OTA methyl ester (**110**) by the cell cultures of wheat and maize [31].

(v) Dechlorination: OTA (**21**) was dechlorinated into OTB (**27**) in the renal microsomes of rabbits pretreated with phenobarbital (PB) [117].

### 3.7. Detoxification of Patulin

Patulin (PAT, **65**), a polyketide lactone, exhibits genotoxic, mutagenic, carcinogenic, teratogenic and cytotoxic properties for humans and animals as well as plants [148,149]. PAT (**65**) can be produced by various fungal species belonging the genera *Penicillium, Aspergillus* and *Byssochlamys*, and particularly, *Penicillium expansum* is considered as the major producer of PAT (**65**), which is causative agent of the blue mold disease of stored apples [150]. Detoxification of PAT (**65**) through biotransformation by using yeast, bacteria and fungi have been shown good results, and it seems to be attractive since it works under mild and environment-friendly conditions [149,151]. If furan or the pyran ring of PAT (**65**) was destroyed, the transformed products became less or non toxic (Figures S30, S31 and S82). The main transformation pathways of PAT (**65**) are as follows.

(i) Oxido-reduction: when PAT (**65**) was added in the cell cultures or cell-free supernatant of *Lactobacillus plantarum*, it was transformed to *E*-ascladiol (**66**) and *Z*-ascladiol (**67**). The ascladiol isomers were then further transformed over a 4-week cell-free incubation (4 °C) into the metabolite hydroascladiol (**68**) [62].

(ii) Multi-step biotransformations: PAT (**65**) was converted into desoxypatulinic acid (DPA, **150**), which was less cytotoxic than PAT (**65**) to *in vitro* human lymphocytes, by the yeast *Rhodotorula kratochvilovae* LS11 [118,120], and fungus *Rhodosporidium paludigenum* [119]. Isotopic labeling experiments revealed that DPA (**150**) derived from PAT (**65**) through hydrolysis of the γ-lactone ring and subsequent enzymatic modifications by reductase and dehydratase [118]. For PAT (**65**) conversion into *E*/*Z*-ascladiols (**66** and **67**) and DPA (**150**), other reported microorganisms included fungi such as *Kodameae ohmeri* [60], *Pichia caribbica* [152], *Rhodotorula mucilaginosa* JM19 [153], *Saccharomyces cerevisiae* [154] and *Sporobolomyces* sp. IAM 13,481 [155], as well as bacteria such as *Lactobacillus plantarum* [62].

### 3.8. Detoxification of Trichothecenes

Trichothecenes are mainly produced by the fungi from the genus of *Fusarium*. Other minor trichothecenes producing species are from the genera of *Cephalosporium*, *Myrothecium*, *Stachybotrys*, *Trichoderma* and *Trichothecium* [156,157,158]. Trichothecenes are commonly found in contaminated cereals, particularly in wheat, barley, oats and maize [15].

Trichothecenes are chemically tricyclic sesquiterpenoids, characterized by a double bond at the C-9 and C-10 position, an epoxy functional group at the C-12 and C-13 position and variable numbers of hydroxyl and acetoxy groups. Trichothecenes can be divided into four types (i.e., types A-D) according to characteristic functional groups [159]. Among them, types A and B are of more concern to humans due to their broad and highly toxicity. Data about their natural occurrence in foods are mostly limited to HT-2 toxin (**122**), T-2 toxin (**31**), DON (**38**), 3-acetyl DON (**112**), 15-acetyl DON, 4,15-DAS (**99**) and nivalenol (NIV, **46**) due to their high toxicity and prevalent occurrence. DON (**38**) may be the most commonly occurring trichothecene in nature. T-2 toxin (**31**) does not occur as much as DON (**38**), but its toxicity is higher than that of DON (**38**) [156,160].

The double bond between C-9 and C-10, the 12, 13-epoxide ring, and hydroxyl group at C-3 have been considered as the toxicity of trichothecenes in eukaryotic organisms [161]. Removal of these groups results in a complete loss of toxicity [159]. Common toxicity effects of the trichothecenes in humans and animals include diarrhea, vomiting, feed refusal, growth retardation, immunosuppression, reduced ovarian functions/reproductive disorders and even death [162]. At the molecular level, trichothecenes bind to the ribosome, induce a ribotoxic stress leading to the activation of MAP kinases, cellular cell-cycle arrest and apoptosis [160].

Detoxification of trichothecenes via various biotransformation pathways mainly include oxygenation [163], de-epoxidation at the C-12 and C-13 positions [50,164], epimerization of hydroxyl group at C-3 position [113], glycosylation of hydroxyl groups [79] and hydrolysis of acetoxy groups [79,86]. Biotransformation systems include several bacteria and fungi along with their isolated enzymes. The main transformation pathways of trichothecenes are as follows.

(i) Glycosylation or glucuronidation: Glycosylation of C-3 hydroxy group by plant glycosyltransferase enzymes can convert trichothecenes into less toxic glycosides [65,70]. DON (**38**) was transformed to different mono-*O*-glucuronides by glucuronidation through animal liver microsomes [71]. Similarly, T-2 toxin (**31**) was transformed to T-2 toxin 3-*O*-β-d- glucuronide (**106**) by UDP glucuronyl transferase (UDPGT) released from rat liver microsomes [80]. In humans and animals, DON (**38**) is dominantly excreted as a glucuronide [165].

(ii) Epimerization: DON (**38**) was converted to 3-*epi*-deoxynivalenol (**143**) with epimerization by the bacterium *Nocardioides* sp. WSN05-2 [113].

(iii) De-epoxidation: transformation of DON (**38**) with de-epoxidaton at the C-12 and C-13 positions by *Eubacterium* sp. DSM 11798. The converted product was de-epoxydeoxynivalenol (DOM, **45**) [50].

(iv) Hydrolysis: T-2 toxin (**31**) was hydrolyzed either into HT-2 toxin (**122**) by the bacterium *Eubacterium* sp. BBSH 797 [86] or into neosolaniol (**125**) by the fungus *Blastobotrys capitulate* [79].

### 3.9. Detoxification of Zearalenone Analogues

Zearalenone (ZEN, **33**) is a macrocyclic phenolic β-resorcyclic acid lactone. It mainly produced by fungi belonging to the genus *Fusarium* such as *F. graminaearum* and *F. culmorum*. It possesses estrogenic activity in pigs, cattle and sheep [166]. Moreover, alcohol metabolites, such as α-zearalenol (**42**) and β-zearalenol (**43**) of ZEN, are also estrogenic and the effects of ZEN (**33**) and related metabolites on animal reproductive function have been reported [166,167]. ZEN (**33**) also damages the liver and kidneys and reduces immune function which leads to cytotoxicity and immunotoxicity [12]. The possible pathways available for ZEN (**33**) biotransformation relate mainly to the hydrolysis of the lactone ring, reduction of the ketonic carbonyl group, modification of the hydroxyl groups (i.e., sulfation and glycosylation), and reduction of the carbon-carbon double bond. The main transformation pathways of ZEN analogues are as follows.

(i) Hydrolysis: ZEN (**33**) was transformed to decarboxylated hydrolyzed ZEN, namely 1-(3,5-dihydroxyphenyl)- 6′-hydroxy-l’- undecen-l0′- one (**154**), at degradation rate of 95.7% by *Bacillus pumilus* ES-21 [122] or purified lactonase [123].

(ii) Reduction of the carbon-carbon double bond: ZEN (**33**) was transformed to zearalanone (**44**) with reduction of the carbon-carbon bond in ovine [49].

(iii) Reduction of the ketonic carbonyl group: ZEN (**33**) was converted to α-zearalenol (**42**) and β-zearalenol (**43**) with reduction of the keto group by the fungi *Rhizopus* sp. and *Aspergillus* sp. [47] and *Candida tropicalis* [46].

(iv) Multi-step oxido-reductions: ZEN (**33**) was transformed to α-zearalenol (**42**), β-zearalenol (**43**), zearalanone (**44**), α-zearalanol (**151**), β-zearalanol (**152**) and with multi-step oxido-reductions in human body [121].

(v) Sulfation: ZEN (**33**) was converted to ZEN 14-sulfate (**127**) by the fungus *Sphaerodes mycoparasitica* [87].

(vi) Methylation: ZEN (**33**) was converted to ZEN 16-methyl ether (**69**) and ZEN 14,16-bis (methyl ether) (**70**) by the fungus *Cunninghamella bainieri*. ZEN 14,16-bis (methyl ether) (**70**) showed an inactive estrogenic effect [38].

(vii) Glycosylation: ZEN (**33**) was converted to zearalenone-14-*O*-glucoside (**107**) by *Arabidopsis* UDP-glucosyltransferases expressed in *Saccharomyces cerevisiae* [81]. ZEN (**33**) was also glycosylated into zearalenone 14-*O*-β-glucoside (**107**) and zearalenone 16-*O*-β-glucoside (**108**) by a barley UDP-glucosyltransferase [83]. The products **107** and **108** of glycosylated ZEN showed nontoxic activity [38].

## 4. Conclusions and Future Perspectives

This review described detoxification of mycotoxins through biotransformation by using bacteria, fungi, plants and animals, as well as the isolated enzymes. As can be seen from the examples given in this review that both organisms and the isolated enzymes possess considerable biochemical potentials to convert mycotoxins. The recently-reported examples, coupled with the emergence of some efficient commercialized biological/enzymatic agents, highlight the promise of this approach to address the safety of animal feed and human food [11,168,169].

The reaction types and stereochemistry of conversion depend on the functional groups in the molecular structures of mycotoxins together with the specific enzymes provided by the organisms. Detoxification through biotransformation is now an important strategy to eliminate mycotoxins from food and feed. Indeed, not all converted products are nontoxic. Some converted products are still toxic or even more toxic [170,171]. Some mycotoxins are converted into the masked forms such as ZEN 14-*O*-glucoside (**107**) and ZEN 16-*O*-glucoside (**108**), which can be accumulated in organisms and cannot be eliminated [81,172,173]. Through the studies of structure-activity relationship (SAR), the essential functional groups for toxicitiy of mycotoxins have been clarified [7,161,174]. This should be the focus of the research on mycotoxin biotransformation for detoxification in the coming years.

Bacteria and fungi that can transform mycotoxins to less toxic or nontoxic products serve as a source of enzymes that can be used to decontaminate agricultural commodities or used as feed additives. More and more degradation enzymes of mycotoxins have been purified and identified from microorganisms [7,175]. The corresponding genes have been cloned and expressed in the engineered microorganisms. Both purified enzymes and engineered strains have potential applications for mycotoxin degradation in food and feed industry. Some genes that control the detoxifications of mycotoxins, such as the genes of trichothecene acetyltransferase (TRI101) [176] and the zearalenone lactonohydrolase (zhd101) [177], have been cloned and expressed in plants to limit pre-harvest contamination of crops.

In plants, sequential hydroxylation, glycosylation and demethylation of fungal phytotoxins can avoid plant cell death and overcome the fungal invader [24]. Detoxification of fungal phytotoxins through biotransformation by plants should be an important plant defensive mechanism against fungal pathogens [25].

Many other mycotoxins such as beauvericin [178,179], enniatins [180], ustiloxins [181], ustilaginoidins [182,183,184,185], sorbicillinoids [186,187] and mycotoxins from mushrooms have not been studied for their detoxification through biotransformation. Although the structures of some converted products from mycotoxins were elucidated, their toxicity to animals and humans are not clear.

In short, detoxification of mycotoxins through biotransformation provides a reliable reference strategy for the management of mycotoxins in foods and feeds. It will help us to better understand the fate of mycotoxins in animals and humans, as well as to provide basic information for the risk assessment of mycotoxins for food and feed safety. Further investigations, especially the development of methods to utilize the multi-reaction processes as well as to clone and express the genes of detoxification enzymes in organisms, will be necessary for the detoxification of mycotoxins through biotransformation.

## Figures and Tables

**Table 1:**
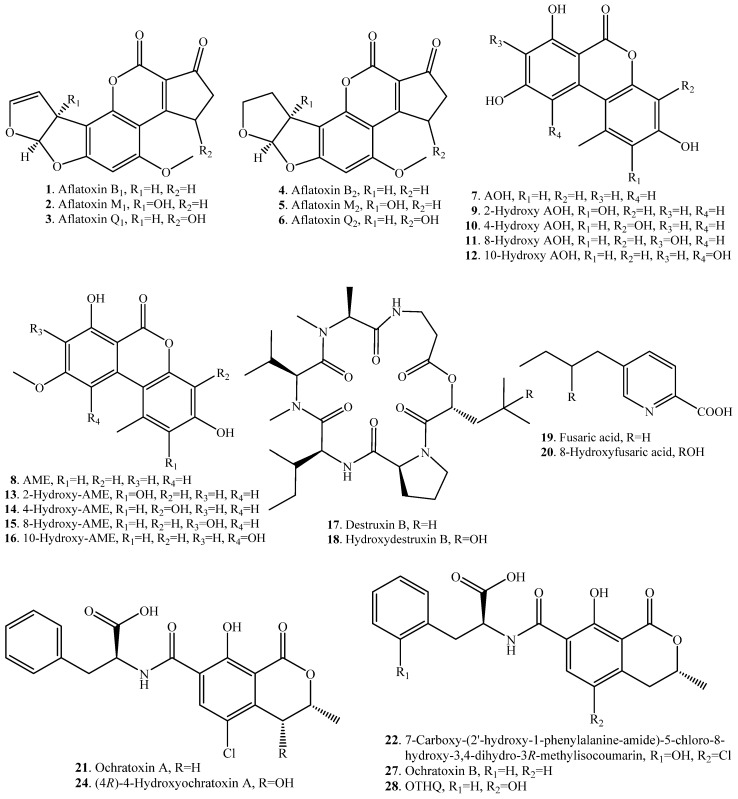

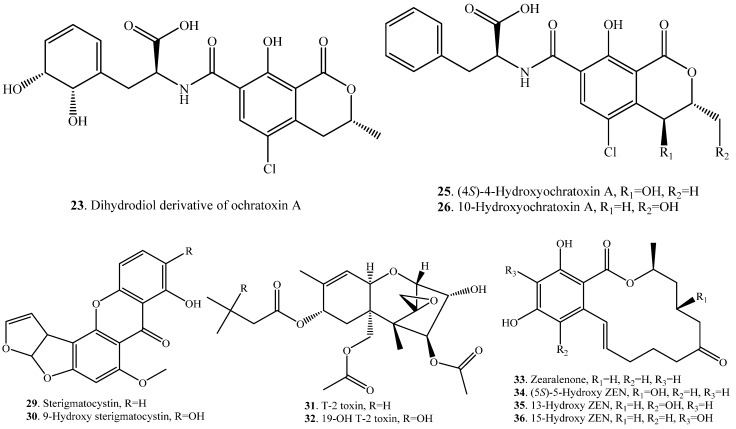
Hydroxylation of mycotoxins.

Substrate	Product	Biotransformation System	Ref.
Aflatoxin B_1_ (AFB_1_, **1**)	Aflatoxins M_1_ (**2**) and Q_1_ (**3**)	Hepatic microsomal mixed-function oxidase of rhesus monkey	[22]
	Aflatoxin M_1_ (**2**)	Channel catfish liver	[20]
	Aflatoxin Q_1_ (**3**)	Rat liver microsomal cytochrome P450p	[21]
Aflatoxin B_2_ (AFB_2_, **4**)	Aflatoxins M_2_ (**5**) and Q_2_ (**6**)	Animal liver microsomes	[14]
Alternariol (AOH, **7**)	2-Hydroxy AOH (**9**)	Microsomes from rat, human and porcine liver	[23]
	4-Hydroxy AOH (**10**)	Microsomes from rat, human and porcine liver	[23]
	8-Hydroxy AOH (**11**)	Microsomes from rat, human and porcine liver	[23]
	10-Hydroxy AOH (**12**)	Microsomes from rat, human and porcine liver	[23]
Alternariol 9-*O*-methyl ether (AME, **8**)	2-Hydroxy AME (**13**)	Microsomes from rat, human and porcine liver	[23]
	4-Hydroxy AME (**14**)	Microsomes from rat, human and porcine liver	[23]
	8-Hydroxy AME (**15**)	Microsomes from rat, human and porcine liver	[23]
	10-Hydroxy AME (**16**)	Microsomes from rat, human and porcine liver	[23]
Destruxin B (**17**)	Hydroxydestruxin B (**18**)	Crucifers such as *Brassica napus*	[24]
Fusaric acid (**19**)	8-Hydroxyfusaric acid (**20**)	*Mucor rouxii* (fungus)	[27]
Ochratoxin A(OTA, **21**)	7-Carboxy-(2′-hydroxy-1- phenylalanine-amide)- 5-chloro-8-hydroxy-3,4-dihydro-3*R*- methylisocoumarin (**22**)	*Phenylobacterium immobile* (bacterium)	[28]
	Dihydrodiol derivative of ochratoxin A (**23**)	*Phenylobacterium immobile* (bacterium)	[28]
	(4*R*)-4-Hydroxyochratoxin A (**24**)	Rat liver microsomes	[29]
		Cell cultures of wheat and maize	[29]
		Rabbit liver microsomes	[30]
	(4*S*)-4-Hydroxyochratoxin A (**25**)	Cell cultures of wheat and maize	[31]
		Rabbit liver microsomes	[30]
	10-Hydroxyochratoxin A (**26**)	Rabbit liver microsomes	[30]
Ochratoxin B (OTB, **27**)	Hydroquinone metabolite of ochratoxin (OTHQ, **28**)	Horse radish peroxidase (HPR)	[32]
Sterigmatocystin (**29**)	9-Hydroxy sterigmatocystin (**30**)	Human and rat hepatic microsomes	[34]
T-2 toxin (**31**)	19-OH T-2 toxin = 3‘-OH T-2 toxin (**32**)	Chicken CYP3A37 (enzyme)	[35]
Zearalenone (ZEN, **33**)	(5*S*)-5-Hydroxy ZEN (**34**)	*Cunninghamella bainieri* (fungus)	[38]
	13-Hydroxy ZEN (**35**)	Human liver microsomes	[40]
	15-Hydroxy ZEN (**36**)	Human liver microsomes	[40]

**Table 2:**
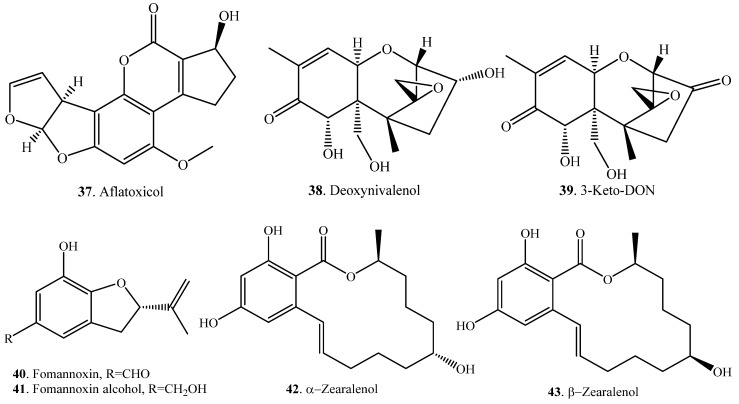
Oxido-reduction between alcohols and ketones of mycotoxins.

Substrate	Product	Biotransformation System	Ref.
Aflatoxin B_1_ (AFB_1_, **1**)	Aflatoxicol (**37**)	Fungi *Aspergillus niger*, *Eurotium herbariorum*, *Rhizopus* sp.	[41]
Deoxynivalenol (DON, **38**)	3-Keto-DON (**39**)	*Devosia mutans* (bacterium)	[42]
Fomannoxin (**40**)	Fomannoxin alcohol (**41**)	*Pinus sylvestris* cell cultures	[44]
		Rhizosphere-associated bacterium *Streptomyces* sp. AcH 505	[48]
Zearalenone(ZEN, **33**)	α-Zearalenol (**42**)	*Candida tropicalis* (fungus)	[45]
		Fungi: *Saccharomyces cerevisae*, *Torulaspora delbruckii*, *Zygosaccharomyces rouxii*, *Pichia fermentans*, and several yeast strains of the genera *Candida*, *Hansenula*, *Brettanomyces*, *Schizosaccharornyces* and *Saccharomycopsis*	[46]
		Fungi *Rhizopus* sp. and *Aspergillus* sp.	[47]
	β-Zearalenol (**43**)	*Candida tropicalis* (fungus)	[45]
		Fungi: *Saccharomyces cerevisae*, *Torulaspora delbruckii* and *Zygosaccharomyces rouxii*	[46]

**Table 3:**
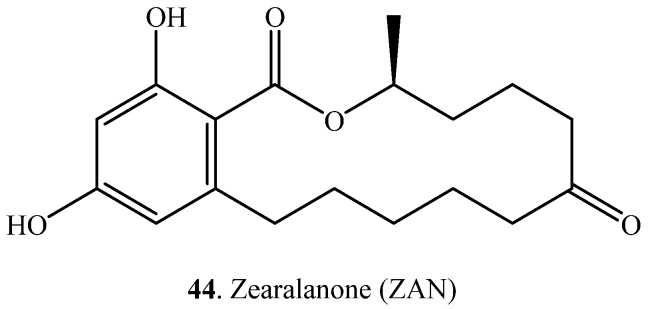
Reduction of the carbon-carbon double bond of mycotoxins.

Substrate	Product	Biotransformation System	Ref.
Aflatxin B_1_ (AFB_1_, **1**)	Aflatoxin B_2_ (AFB_2_, **4**)	*Penicillium raistrickii* (fungus)	[14]
Zearalenone (ZEN, **33**)	Zearalanone (ZAN, **44**)	Ovine	[49]

**Table 4:**
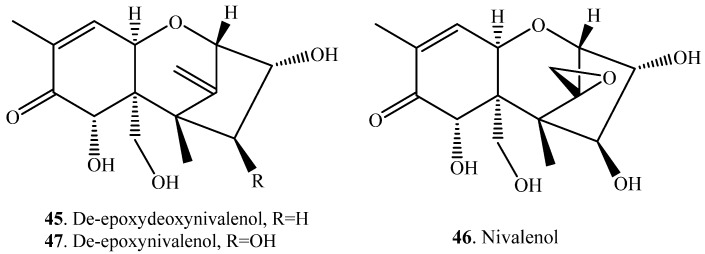
De-epoxidation of mycotoxins.

Substrate	Product	Biotransformation System	Ref.
Deoxynivalenol (DON, **38**)	Deepoxydeoxynivalenol (DOM, **45**)	*Eubacterium* sp. DSM 11,798 (bacterium)	[50]
Nivalenol (NIV, **46**)	De-epoxy NIV (**47**)	*Euacterium* sp. BBSH 797 (bacterium)	[51]
		Wistar rats	[53]

**Table 5:**
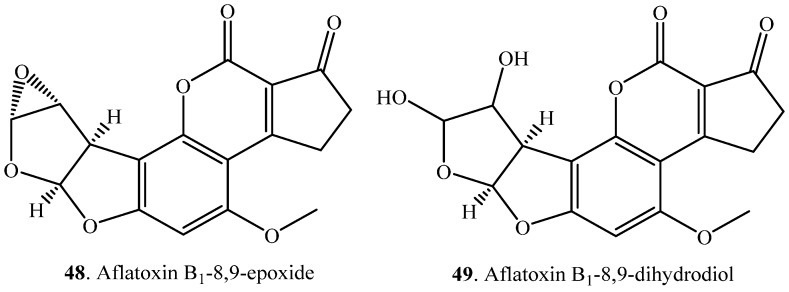

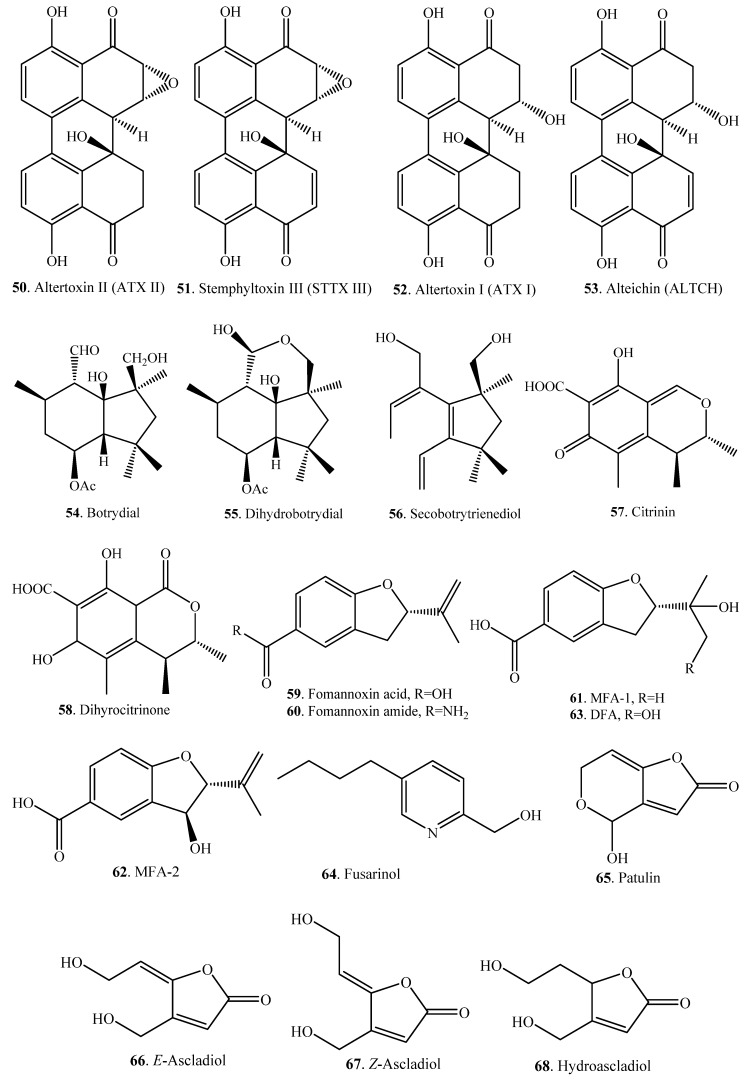
Other oxido-reductions of mycotoxins.

Substrate	Product	Biotransformation System	Ref.
Aflatoxin B_1_ (AFB_1_, **1**)	AFB_1_-8,9-epoxide (**48**)	Channel catfish liver	[20]
	AFB_1_-8,9-dihydrodiol (**49**)	*Phanerochaete sordida* YK-624 (fungus)	[54]
Altertoxin II (**50**)	Altertoxin I (**52**)	Mammalian cell lines Caco-2, HCT 116, HepG2, V79	[55]
Stemphyltoxin III (**51**)	Alteichin (**53**)	Mammalian cell line Caco-2	[55]
Botrydial (**54**)	Dihydrobotrydial (**55**)	*Botrytis cinerea* (fungus)	[56]
	Secobotrytrienediol (**56**)	*Botrytis cinerea* (fungus)	[56]
Citrinin (**57**)	Dihydrocitrinone (**58**)	Rats and humans	[57]
Fomannoxin (**40**)	Fomannoxin acid (**59**)	Rhizosphere-associated bacterium *Streptomyces* sp. AcH 505	[48]
	Fomannoxin amide (**60**)	Rhizosphere-associated bacterium *Streptomyces* sp. AcH 505	[48]
	MFA-1 (**61**)	Rhizosphere-associated bacterium *Streptomyces* sp. AcH 505	[48]
	MFA-2 (**62**)	Rhizosphere-associated bacterium *Streptomyces* sp. AcH 505	[48]
	DFA (**63**)	Rhizosphere-associated bacterium *Streptomyces* sp. AcH 505	[48]
Fusaric acid (**19**)	Fusarinol (**64**)	*Aspergillus tubingensis* (fungus)	[59]
Patulin (**65**)	*E*-Ascladiol (**66**), *Z*-ascladiol (**67**)	*Kodameae ohmeri* (fungus)	[60]
	*E*-Ascladiol (**66**), *Z*-ascladiol (**67**), hydroascladiol (**68**)	*Lactobacillus plantarum* (bacterium)	[62]

**Table 6:**
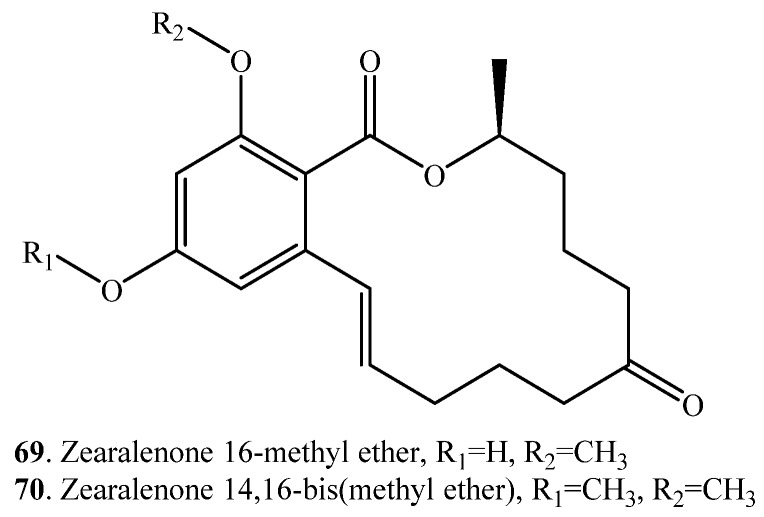
Methylation of mycotoxins.

Substrate	Product	Biotransformation System	Ref.
Alternariol (AOH, **7**)	Alternariol 9-*O*-methyl ether (AME, **8**)	Methyltransferase	[64]
Zearalenone (ZEN, **33**)	Zearalenone 16-methyl ether (**69**)	*Cunninghamella bainieri* (fungus)	[38]
	Zearalenone 14,16-bis (methyl ether) (**70**)	*Cunninghamella bainieri* (fungus)	[38]

**Table 7:**
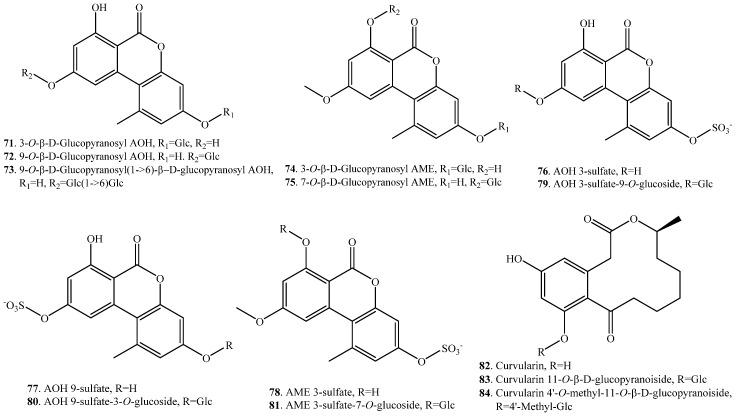

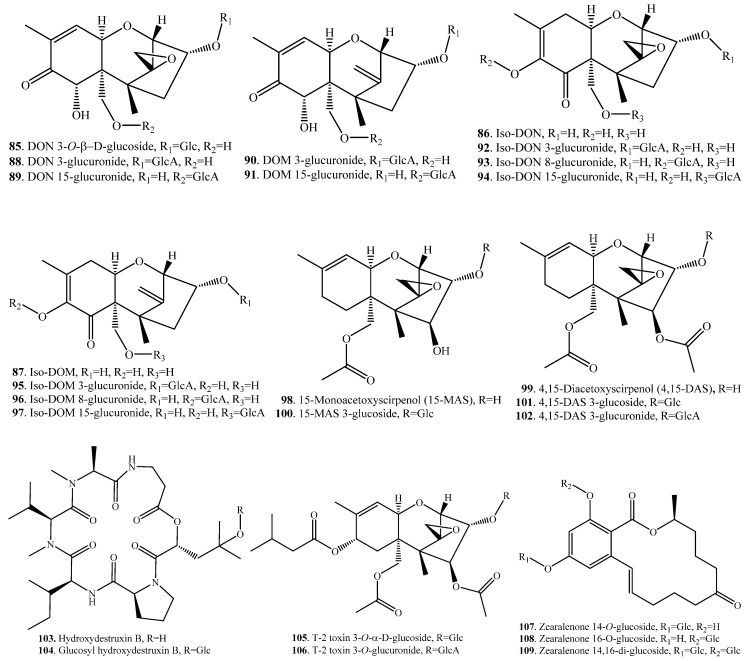
Glycosylation and glucuronidation of mycotoxins.

Substrate	Product	Biotransformation System	Ref.
Alternariol (AOH, **7**)	3-*O*-β-d-Glucopyranosyl alternariol (**71**)	Suspension cell cultures of *Nicotiana tabacum*	[66]
	9-*O*-β-d-Glucopyranosyl alternariol (**72**)	Suspension cell cultures of *Nicotiana tabacum*	[66]
	9-*O*-β-d-Glucopyranosyl (1→6)-β-d-glucopyranosyl alternariol (**73**)	Suspension cell cultures of *Nicotiana tabacum*	[66]
Alternariol 9-*O*-methyl ether (AME, **8**)	3-*O*-β-d-Glucopyranosyl AME (**74**)	Suspension cell cultures of *Nicotiana tabacum*	[66]
	7-*O*-β-d-Glucopyranosyl AME (**75**)	Suspension cell cultures of *Nicotiana tabacum*	[66]
AOH 3-sulfate (**76**)	AOH 3-sulfate 9-*O*-glucoside (**79**)	Tomato tissues and cultured tobacco cells	[67]
AOH 9-sulfate (**77**)	AOH 9-sulfate 3-*O*-glucoside (**80**)	Tomato tissues and cultured tobacco cells	[67]
AME 3-sulfate (**78**)	AME 3-sulfate 7-*O*-glucoside (**81**)	Tomato tissues and cultured tobacco cells	[67]
Curvularin (**82**)	Curvularin 11-O-β-d-glucopyranoside (**83**)	*Beauveria bassiana* (fungus)	[69]
	Curvularin 4‘-*O*-methyl-11-*O*-β-d-glucopyranoside (**84**)	*Beauveria bassiana* (fungus)	[69]
Deoxynivalenol (DON, **38**)	DON 3-*O*-β-d-glucoside (**85**)	A combinant UDP-gluosyltransferase from rice	[70]
	DON-3-GlcA (**88**)	Rat liver microsomes (RLM), human liver microsomes (HLM)	[71]
	DON-15-GlcA (**89**)	RLM, HLM	[71]
Deepoxy-deoxynivalenol (DOM, **45**)	DOM-3-GlcA (**90**)	RLM, HLM	[71]
	DOM-15-GlcA (**91**)	RLM, HLM	[71]
Iso-DON (**86**)	Iso-DON-3-GlcA (**92**)	RLM, HLM	[71]
	Iso-DON-8-GlcA (**93**)	RLM	[71]
	Iso-DON-15-GlcA (**94**)	RLM, HLM	[71]
Iso-DOM (**87**)	Iso-DOM-3-GlcA (**95**)	RLM, HLM	[71]
	Iso-DOM-8-GlcA (**96**)	RLM	[71]
	Iso-DOM-15-GlcA (**97**)	RLM, HLM	[71]
15-Monoacetoxyscirpenol (15-MAS, **98**)	15-MAS 3-glucoside (**100**)	Corn (*Zea Mays*) plants	[75]
4,15-Diacetoxyscirpenol (4,15-DAS, **99**)	4,15-DAS 3-glucoside (**101**)	Corn (*Zea Mays*) plants	[75]
	4,15-DAS 3-Glucuronide (**102**)	Rats	[78]
Hydroxydestruxin B (**103**)	Glucosyl hydroxydestruxin B (**104**)	Crucifers such as *Brassica napus*	[24]
T-2 toxin (**31**)	T-2 toxin 3-*O*-α-d-glucoside (**105**)	Fungi *Blastobotrys muscicola*, *B. robertii*	[79]
	T-2 toxin 3-*O*-glucuronide (T-2 GlcA, **106**)	Rat hepatic microsomes	[80]
Zearalenone (ZEN, **33**)	ZEN 14-*O*-glucoside (**107**)	*Arabidopis* UDP-glucosyltransferase	[81]
		*Mucor bainieri* (fungus)	[82]
		*Thamnidium elegans* (fungus)	[82]
		Barley UDP-glucosyltransferase	[83]
	ZEN 16-*O*-glucoside (**108**)	Barley UDP-glucosyltransferase	[83]
	ZEN 14,16-di-glucoside (**109**)	Recombinant barley glucosyltransferase	[84]

**Table 8:**
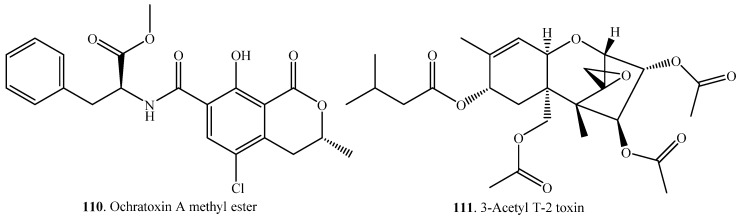
Esterification of mycotoxins.

Substrate	Product	Biotransformation System	Ref.
Ochratoxin A (**21**)	Ochratoxin A methyl ester (**110**)	Cell cultures of wheat and maize	[31]
T-2 toxin (**31**)	3-Acetyl T-2 toxin (**111**)	Bovine rumen fluid *in vitro*	[85]

**Table 9:**
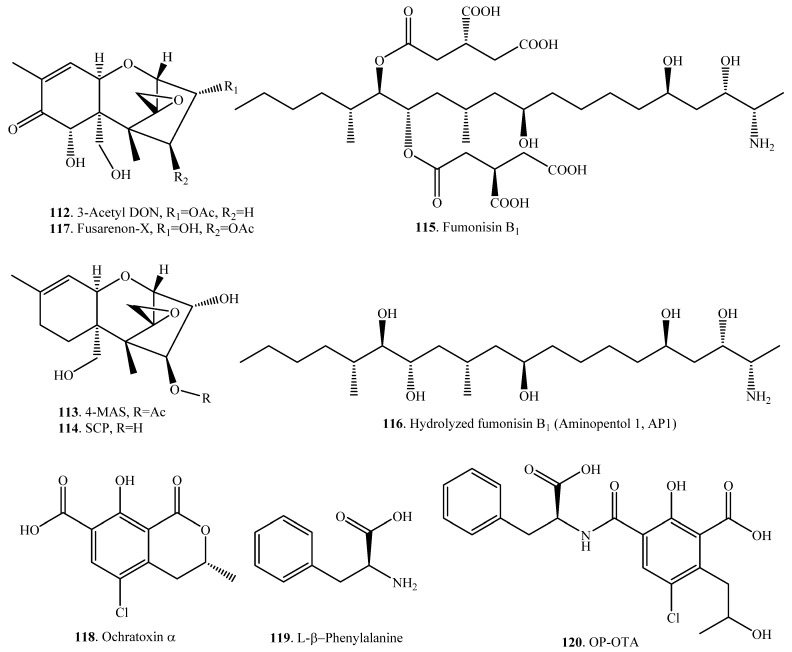

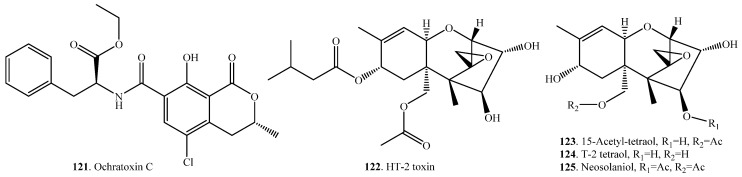
Hydrolysis of mycotoxins.

Substrate	Product	Biotransformation System	Ref.
3-Acetyl DON (**112**)	DON (**38**)	Cell-free extracts of the fungus *Fusarium* sp.	[88]
		*Sphaerodes mycoparasitica* (fungus)	[87]
4,15-Diacetoxyscirpenol (4,15-DAS, **99**)	4-Monoacetoxyscirpenol (4-MAS, **113**)	Rats	[78]
	15-Monoacetoxyscirpenol (15-MAS, **98**)	Rats	[78]
	Scirpentriol (SCP, **114**)	Rats	[78]
Fumonisin B_1_ (**115**)	Hydrolyzed fumonisin B_1_ = Aminopentol 1 (AP1, **116**)	*Exophiala spinifera* 2141.10 (fungus)	[89]
		Hydroxylase from the bacterium *Sphingopyxis* sp. MTA144	[90]
		Carboxylesterase FumD	[91]
Fusarenon-X (FX, (**117**)	Nivalenol (NIV, **46**)	Mice	[92]
		Goat (*Capra hircus*)	[93]
Ochratoxin A (OTA, **21**)	Ochratoxin α (**118**) and L-β-phenylalanine (**119**)	Crude lipase from *Aspergillus niger*	[94]
		Protease A prolyve PAC and pancreatin	[95]
		Carboxypeptidase A	[96]
		Fungus: *Aspergillus niger* (fungus)	[97]
		*Bacillus amyloliquefaciens* (bacterium)	[98]
	Lactone-opened ochratoxin A (OP-OTA, **120**)	Rats	[99]
Ochratoxin C (OTC) = Ochratoxin A ethyl ester (**121**)	Ochratoxin A (OTA, **21**)	Rats	[100]
T-2 toxin (**31**)	HT-2 toxin (**122**)	*Eubacterium* BBSH 797 (bacterium)	[86]
	HT-2 toxin (**122**),15-acetyl-tetraol (**123**),T-2 tetraol (**124**)	Liver and intestines of rats	[102]
	Neosolaniol (**125**)	*Blastobotrys capitulate* (fungus)	[79]

**Table 10:**
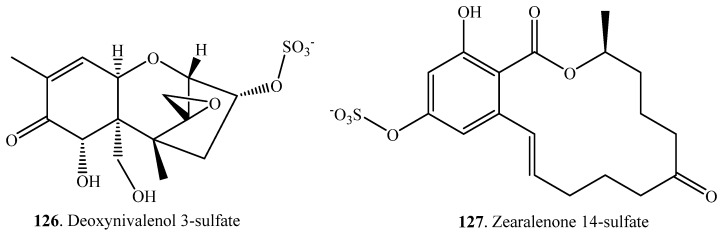
Sulfation of mycotoxins.

Substrate	Product	Biotransformation System	Ref.
Deoxynivalenol (**38**)	Deoxynivlenol 3-sulfate (**126**)	*Sphaerodes mycoparasitica* (fungus)	[87]
Zearalenone (**33**)	Zearalenone 14-sulfate (**127**)	*Sphaerodes mycoparasitica* (fungus)	[87]
		Pigs	[103]

**Table 11:**
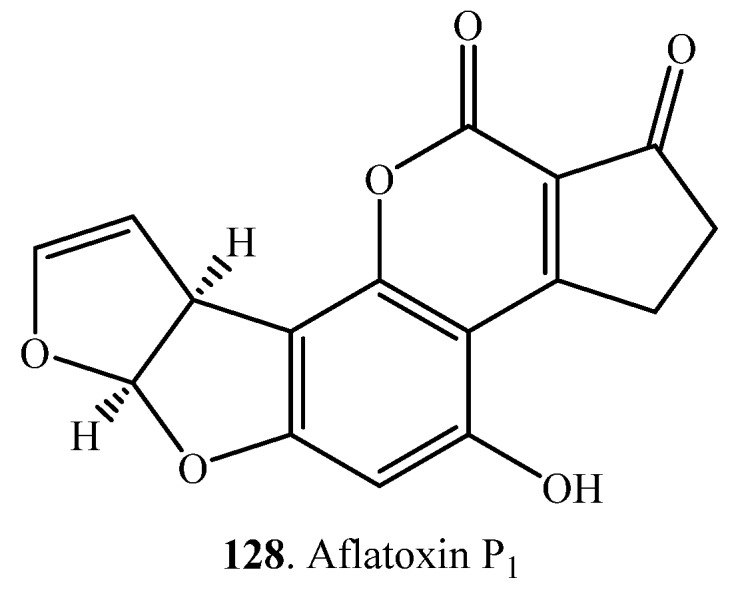
Demethylation of mycotoxins.

Substrate	Product	Biotransformation System	Ref.
AME (**8**)	AOH (**7**)	Homogenate of porcine liver in the presence of NADPH	[104]
AFB_1_ (**1**)	Aflatoxin P_1_ (AFP_1_, **128**)	Enzyme CYP321A1 from *Helicoverpa zea*	[105]

**Table 12:**
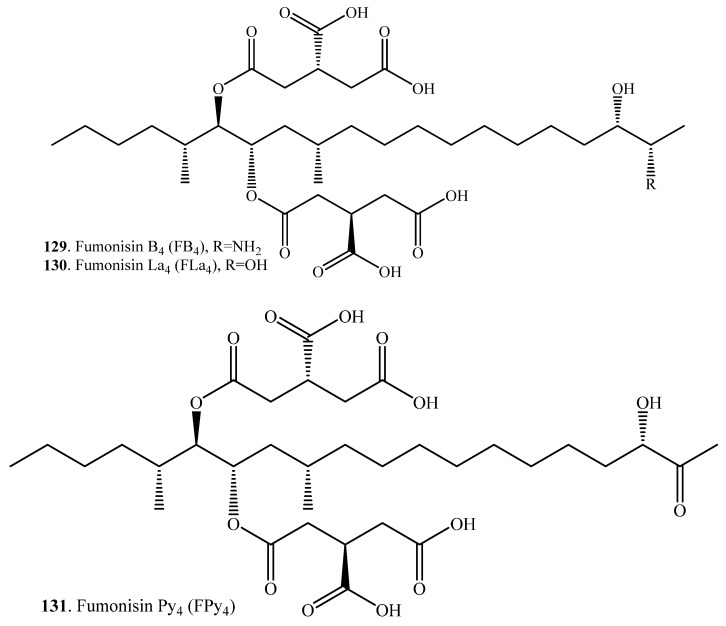

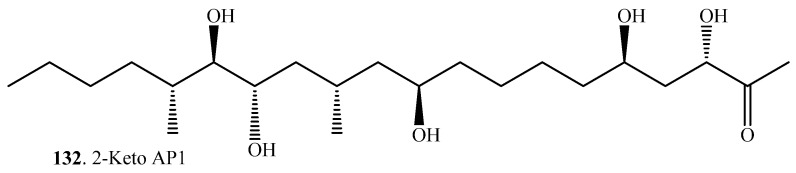
Deamination of mycotoxins.

Substrate	Product	Biotransformation System	Ref.
Fumonisin B_4_ (**129**)	Fumonisin La_4_ (FLa_4_, **130**)	*Aspergillus* sp. (fungus)	[106]
	Fumonisin Py_4_ (FPy_4_, **131**)	*Aspergillus* sp. (fungus)	[106]
Hydrolyzed fumonisin B_1_ = Aminopentol 1 (AP1, **116**)	2-Keto HFB_1_ = 2-keto AP1 (**132**)	*Exophiala spinifera* (fungus)	[107]

**Table 13:**
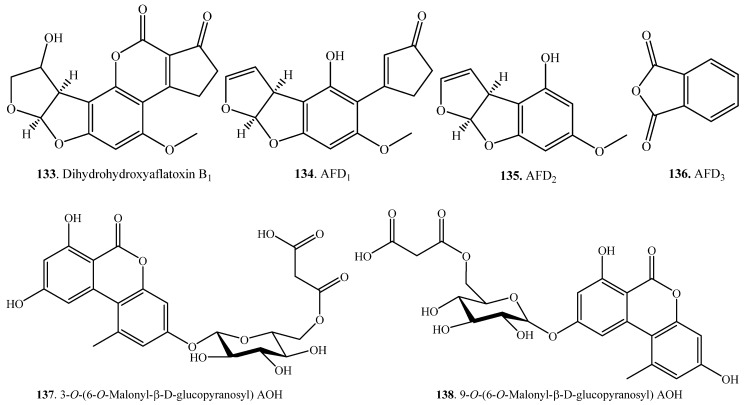

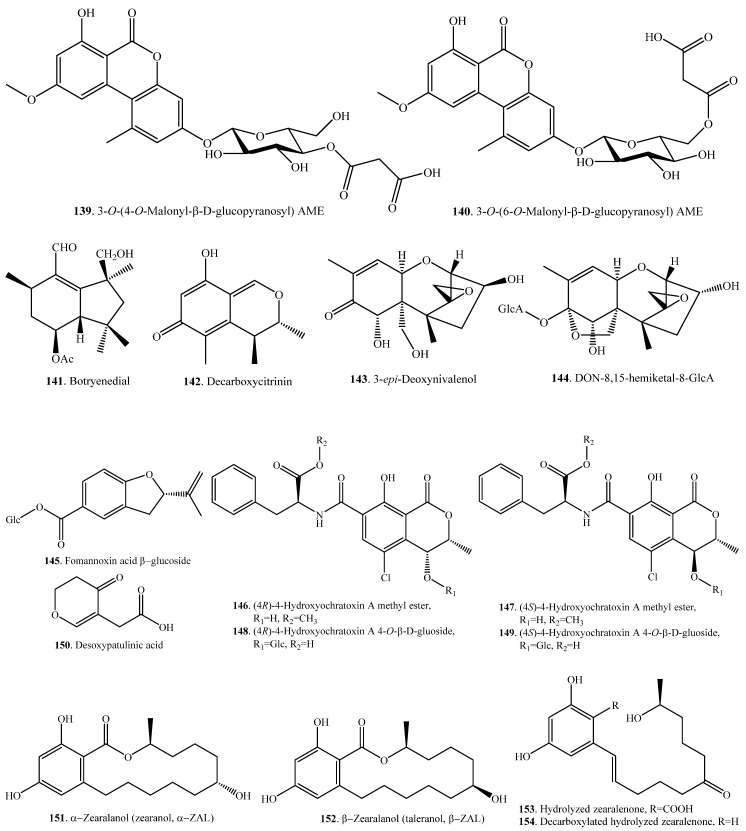
Miscellaneous biotransformation of mycotoxins.

Substrate	Product	Type	Biotransformation System	Ref.
Aflatoxin B_1_ (AFB_1_, **1**)	Dihydrohydroxyalfatoxin B_1_ (AFB_2a_, **133**)	Reduction and oxidation	*Pleurotus ostreatus* (fungus)	[86]
	AFD_1_ (**134**), AFD_2_ (**135**), and AFD_3_ (**136**)	Hydolysis, decarboxylation, oxidation-reduction	*Pseudomonas putida* (bacterium)	[110]
Alternariol (AOH, **7**)	3-*O*-(6-*O*-Malonyl-β-d-glucopyranosyl) AOH (**137**)	Glycosylation and Esterification	Suspension cell cultures of *Nicotiana tabacum*	[66]
	9-*O*-(6-*O*-Malonyl-β-d-glucopyranosyl) AOH (**138**)	Glycosylation and Esterification	Suspension cell cultures of *Nicotiana tabacum*	[66]
Alternariol 9-*O*-methyl ether = AME (**8**)	3-*O*-(4-*O*-Malonyl-β-d-glucopyranosyl) AME (**139**)	Glycosylation and Esterification	Suspension cell cultures of *Nicotiana tabacum*	[66]
	3-*O*-(6-*O*-Malonyl-β-d-glucopyranosyl) AME (**140**)	Glycosylation and Esterification	Suspension cell cultures of *Nicotiana tabacum*	[66]
Botrydial (**54**)	Botryenedial (**141**)	Dehydration	*Botrytis cinerea* (fungus)	[56]
Citrinin (**57**)	Decarboxycitrinin (**142**)	Decarboxylation	*Moraxella* sp. MB1 (bacterium)	[111]
Deoxynivalenol (DON, **38**)	3-*epi*-Deoxynivalenol (**143**)	Epimerization	*Nocardioides* sp. WSN05-2 (bacterium)	[113]
			*Devosia* sp. (bacterium)	[114]
	DON-8,15-hemiketal-8-GlcA (**144**)	Oxidation and glucuronidation	Rat liver microsomes (RLM)	[71]
	Iso-DON (**86**)	Isomerization	RLM	[71]
	Iso-DON-3-GlcA (**92**) and iso-DON-8-GlcA (**93**)	Isomerization and glucuronization	RLM	[71]
Deepoxy-deoxynivalenol (DOM, 45)	Iso-DOM (**87**)	Isomerization	RLM	[71]
	Iso-DOM-3-GlcA (**95**) and iso-DOM-8-GlcA (**96**)	Isomerization and glucuronization	RLM	[71]
Fomannoxin (**40**)	Fomannoxin acid (**59**) and fomannoxin acid β-glucoside (**145**)	Oxidation and glycosylation	Cell cultures of *Pinus sylvestris*	[44]
Fumonisin B_1_ (**115**)	Hydrolyzed fumonisin B_1_ = Aminopentol 1 (AP1, **116**) and 2-keto AP1 (**132**)	Hydolysis and deamination	Recombinant enzymes from the bacterium *Sphingopyxis* sp.	[87]
Ochratoxin A (OTA, **21**)	(4*R*)-4-Hydroxyochratoxin A methyl ester (**146**)	Hydroxylation and esterification	Cell cultures of wheat and maize	[31]
	(4*S*)-4-Hydroxyochratoxin A methyl ester (**147**)	Hydroxylation and esterification	Cell cultures of wheat and maize	[31]
	(4*R*)-4-Hydroxyochratoxin A 4-*O*–β-d-glucoside (**148**)	Hydroxylation and glycosylation	Cell cultures of wheat and maize	[31]
	(4*S*)-4-Hydroxyochratoxin A 4-*O*-β-d-glucoside (**149**)	Hydroxylation and glycosylation	Cell cultures of wheat and maize	[31]
	Ochratoxin B (OTB, **27**)	Dechlorination	Renal microsomes	[117]
Patulin (**65**)	Desoxypatulinic acid (**150**)	Hydolysis, reduction and dehydration	*Rhodotorula kratochvilovae* (fungus)	[118]
		Hydolysis, reduction and dehydration	*Rhodosporidium paludigenum* (fungus)	[119]
Zearalenone (ZEN, **33**)	α-Zearalenol (**42**), β-zearalenol (**43**), zearalanone (**44**), α-zearalanol (**151**), and β-zearalanol (**152**)	Reduction and oxidation	Human	[121]
	Hydrolyzed ZEN (**153**) and decarboxylated hydrolyzed ZEN (**154**)	Hydrolysis, spontaneous decarboxylation	*Bacillus pumilus* (bacterium)	[122]
		Hydrolysis, spontaneous decarboxylation	Lactonase	[123]
		Hydrolysis, spontaneous decarboxylation	Lactonase	[124]

## Supplementary Materials

The following are available online at https://www.mdpi.com/2072-6651/12/2/121/s1, Figure S1. Transformation of aflatoxin B_1_ (**1**) by hepatic microsomal mixed-function oxidase of rhesus monkey, Figure S2. Transformation of aflatoxin B_2_ (**4**) with hydroxylation by animal liver microsomes, Figure S3. Transformation of alternariol (**7**) with hydroxylation by the microsomes from rat, human and porcine livers, Figure S4. Transformation of alternariol 9-*O*-methyl ether (**8**) with hydroxylation by the microsomes from rat, human and porcine livers, Figure S5. Transformation of destruxin B (**17**) with hydroxylation by crucifers such as *Brassica napus*, Figure S6. Transformation of fusaric acid (**19**) with hydroxylation by *Mucor rouxii*, Figure S7. Transformation of ochratoxin A (**21**) with hydroxylation by *Phenylobacterium immobile*, Figure S8. Transformation of ochratoxin A (**21**) with hydroxylation by rat liver microsomes, Figure S9. Transformation of ochratoxin A (**21**) with hydroxylation by rabbit liver microsomes, Figure S10. Transformation of ochratoxin B (**27**) with hydroxylation by horse radish peroxidase, Figure S11. Transformation of sterigmatocystin (**29**) with hydroxylation by human and rat hepatic microsomes, Figure S12. Transformation of T-2 toxin (**31**) with hydroxylation by chicken CYP3A37, Figure S13. Transformation of zearalenone (**33**) with hydroxylation by *Cunninghamella bainieri*, Figure S14. Transformation of zearalenone (**33**) with hydroxylation by human liver microsomes, Figure S15. Transformation of aflatoxin B_1_ (**1**) with reduction by several fungi, Figure S16. Transformation of deoxynivalenol (**38**) with oxidation by *Devosia* sp., Figure S17. Transformation of fomannoxin (**40**) with reduction by cell cultures of *Pinus sylvestris*, Figure S18. Transformation of zearalenone (**33**) with reduction by *Candida tropicalis*, Figure S19. Transformation of aflatoxin B_1_ (**1**) with reduction by the fungus *Penicillium raistrickii*, Figure S20. Transformation of zearalenone (**33**) with reduction in ovine, Figure S21. Transformation of deoxynivalenol (**38**) with de-epoxidaton by *Eubacterium* sp. DSM 11798, Figure S22. Transformation of nivalenol (**46**) with de-epoxidation by the bacterium *Eubacterium* sp. BBSH 797 and Wistar rats, Figure S23. Transformation of aflatoxin B_1_ (**1**) with oxidation by channel catafish microsomes, Figure S24. Transformation of aflatoxin B_1_ (**1**) with oxidation by *Phanerochaete sordida* YK-624, Figure S25. Transformation of altertoxin II (**50**) and stemphyltoxin III (**51**) with reduction by mammalian cells, Figure S26. Transformation of botrydial (**54**) with oxido-reductions by *Botrytis cinereal*, Figure S27. Transformation of citrinin (**57**) with oxido-reduction in the urine of rats and humans, Figure S28. Transformation of fomannoxin (**40**) with oxido-reduction by rhizosphere-associated *Streptomyces* sp. AcH 505, Figure S29. Transformation of fusaric acid (**19**) with reduction by *Aspergillus tubingensis*, Figure S30. Transformation of patulin (**65**) with reduction by the yeast *Kodameae ohmeri*, Figure S31. Transformation of patulin (**65**) with reduction by the bacterium *Lactobacillus plantarum*, Figure S32. Transformation of alternariol (**7**) with methylation by *O*-methyltransferase, Figure S33. Transformation of zearalenone (**33**) with methylation by *Cunninghamella bainieri*, Figure S34. Transformation of alternariol (**7**) with glycosylation by suspension cell cultures of *Nicotiana batacum*, Figure S35. Transformation of alternariol 9-*O*-methyl ether (**8**) with glycosylation by suspension cell cultures of *Nicotiana batacum*, Figure S36. Transformation of alternariol 3-sulfate (**76**), alternariol 9-sulfate (**77**), and alternariol 9-*O*-methyl ether 3-sulfate (**78**) by tomato tissues and cultured tobacco cells, Figure S37. Transformation of curvularin (**82**) with glycosylation or methylglycosylation by *Beauveria bassiana*, Figure S38. Transformation of deoxynivalenol (**38**) with glycosylation by a recombinant UDP-glucosyltransferase from rice, Figure S39. Transformation of deoxynivalenol (DON, **38**) with glucuronidation by rat and human liver microsomes, Figure S40. Transformation of deepoxy-deoxynivalenol (DOM, **45**) with glucuronidation by rat and humam liver microsomes, Figure S41. Transformation of iso-deoxynivalenol (iso-DON, **86**) with glucuronidation by rat and human liver microsomes, Figure S42. Transformation of iso-deepoxy-deoxynivalenol (iso-DOM, **87**) with glucuronidation by rat and humam liver microsomes, Figure S43. Transformation of 15-monoacetoxyscirpenol (**98**) and 4,15-diacetoxyscirpenol (**99**) with glycosylation respectively by corn plants, Figure S44. Transformation of 4,15-diacetoxyscirpenol (**99**) with glucuronidation in rats, Figure S45. Transformation of hydroxydestruxin B (**103**) with glycosylation by crucifers such as *Brassica napus*, Figure S46. Transformation of T-2 toxin (**31**) with glycosylation by *Blastobotrys muscicola* and *B. robertii*, Figure S47. Transformation of T-2 toxin (**31**) with glucuronidation by rat liver microsomes, Figure S48. Transformation of zeralenone (**33**) by *Arabidopsis* UDP-glucosyltransferases expressed in *Saccharomyces cerevisiae*, Figure S49. Transformation of zeralenone (**33**) by barley UDP-glucosyltransferases expressed in *Saccharomyces cerevisiae*, Figure S50. Transformation of zeralenone 14-*O*-glucoside (**107**) and zeralenone 16-*O*-glucoside (**108**) by the recombinant barley glucosyltransferases, Figure S51. Transformation of ochratoxin A (**21**) with esterification by the cell cultures of wheat and maize, Figure S52. Transformation of T-2 toxin (**31**) with esterification (acetylation) by *Pleurotus ostreatus*, Figure S53. Transformation of 3-acetyl-deoxynivalenol (**112**) with hydrolysis (deacetylation) by cell-free extract of *Fusarium* sp., Figure S54. Transformation of 4,15-diacetoxyscirpenol (**99**) with hydrolysis (deacetylation) in rats, Figure S55. Transformation of fumonisin B_1_ (**115**) with hydrolysis by the enzyme from *Sphingopyxis* sp. MTA144, Figure S56. Transformation of fusarenon-X (**117**) with hydrolysis (deacetylation) in animal liver, Figure S57. Transformation of ochratoxin A (**21**) with hydrolysis by enzymes, Figure S58. Transformation of ochratoxin A (**21**) with hydrolysis in rats, Figure S59. Transformation of ochratoxin C (**121**) with hydrolysis in rats, Figure S60. Transformation of T-2 toxin (**31**) with deacetylation by *Eubacterium* sp. 797, Figure S61. Transformation of T-2 toxin (**31**) with multi-step hydrolysis in rat liver and intestines, Figure S62. Transformation of T-2 toxin (**31**) with deesterification by the fungus *Blastobotrys capitulate*, Figure S63. Both deoxynivalenol (DON, **38**) and zearalenone (ZEN, **33**) were converted to their corresponding sulfates DON 3-sulfate (**126**) and ZEN 14-sulfate (**127**) by the fungus *Sphaerodes mycoparasitica*. ZEN (**33**) was converted to ZEN 14-sulfate (**127**) by pigs, Figure S64. Transformation of alternariol 9-*O*-methylether (AME, **8**) with demethylation by the homogenate of porcine liver in the presence of NADPH, Figure S65. Transformation of aflatoxin B_1_ (**1**) with demethylation by CYP21A1 in *Helicoverpa zea*, Figure S66. Transformation of fumonisin B4 (**129**) with oxidative deamination by *Aspergillus* sp, Figure S67. Transformation of aminopentol 1 (AP1, **116**) with oxidative deamination by *Exophiala spinifera*, Figure S68. Transformation of aflatoxin B_1_ (**1**) with reduction and oxidation by *Pleurotus ostreatus* GHBBF10, Figure S69. Transformation of aflatoxin B_1_ (**1**) with hydrolysis, decarboxylation and oxidation-reduction by *Pseudomonas putida*, Figure S70. Transformation of alternariol (**7**) through glycosylation and esterification by suspension cell cultures of *Nicotiana batacum*, Figure S71. Transformation of alternariol 9-*O*-methyl ether (**8**) through glycosylation and esterification by suspension cell cultures of *Nicotiana batacum*, Figure S72. Transformation of botrydial (**54**) with dehydration by *Botrytis cinereal*, Figure S73. Transformation of citrinin (**57**) with decarboxylation by *Moraxella* sp. MB1, Figure S74. Transformation of deoxynivalenol (**38**) with epimerization by *Nocardioies* sp. WSN05-2, Figure S75. Transformation of deoxynivalenol (DON, **38**) with oxidation and glucuronidation by rate liver microsomes, Figure S76. Transformation of deoxynivalenol (DON, **38**) with isomerization and glucuronidation by rate liver microsomes, Figure S77. Transformation of deepoxy-deoxynivalenol (DOM, **45**) with isomerization and glucuronidation by rate liver microsomes, Figure S78. Transformation of fomannoxin (**40**) with oxidation and glycosylation by cell cultures of *Pinus sylvestris*, Figure S79. Transformation of fumonisin B_1_ (**115**) with hydrolysis and deamination by the recombinant enzymes from the bacterium *Sphingopyxis* sp. MTA144, Figure S80. Transformation of ochratoxin A (**21**) with hydroxylation further esterification or glycosylation by the cell cultures of wheat and maize, Figure S81. Transformation of ochratoxin A (**21**) with dechlorination in the renal microsomes, Figure S82. Transformation of patulin (**65**) with hydrolysis, reduction and dehydration by *Rhodotorla kratochvilovae*, Figure S83. Transformation of zearalenone (**33**) with multi-step oxido-reductions in human body, Figure S84. Transformation of zearalenone (**33**) with hydrolysis and decarboxylation by *Bacillus pumilus*.
