# Supplementary material for: Detoxification of Mycotoxins through Biotransformation
Source: Toxins (Basel). 2020 Feb 14;12(2):121. doi: 10.3390/toxins12020121 (PMC7076809; doi:10.3390/toxins12020121)
Supplement: Supplementary file 1 [file toxins-12-00121-s001.pdf]

Supplementary Materials: Detoxification of Mycotoxins through Biotransformation

Peng Li, Ruixue Su, Ruya Yin, Daowan Lai, Mingan Wang, Yang Liu and Ligang Zhou

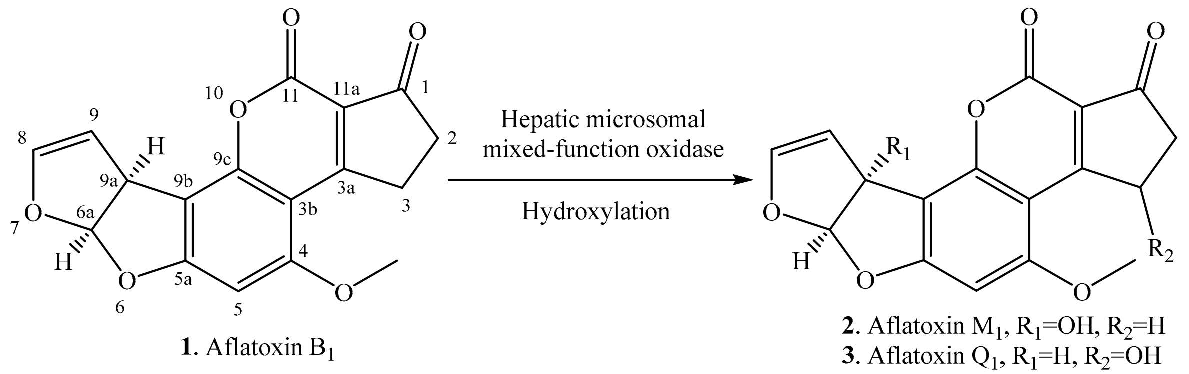

1. Aflatoxin B_1

2. Aflatoxin M_1, R_1=OH, R_2=H
3. Aflatoxin Q_1, R_1=H, R_2=OH

Figure S1. Transformation of aflatoxin B_1 (**1**) by hepatic microsomal mixed-function oxidase of rhesus monkey [1].

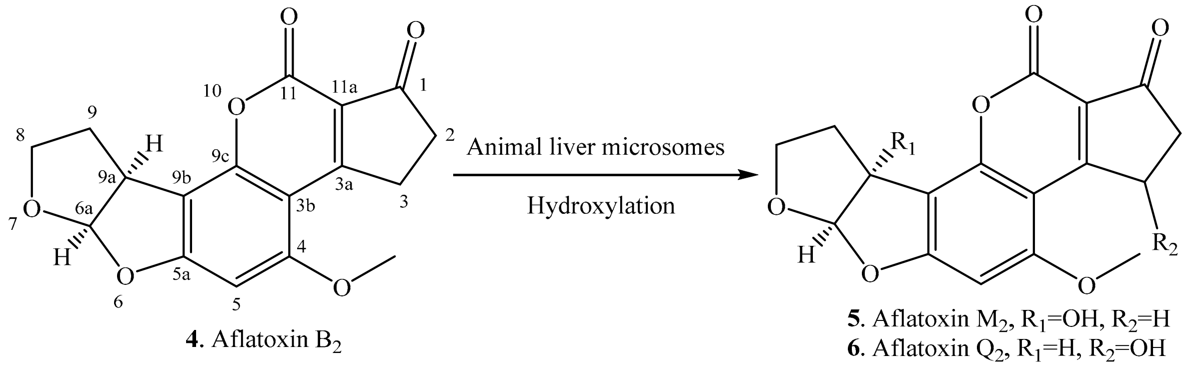

4. Aflatoxin B_2

5. Aflatoxin M_2, R_1=OH, R_2=H
6. Aflatoxin Q_2, R_1=H, R_2=OH

Figure S2. Transformation of aflatoxin B_2 (**4**) with hydroxylation by animal liver microsomes [2].

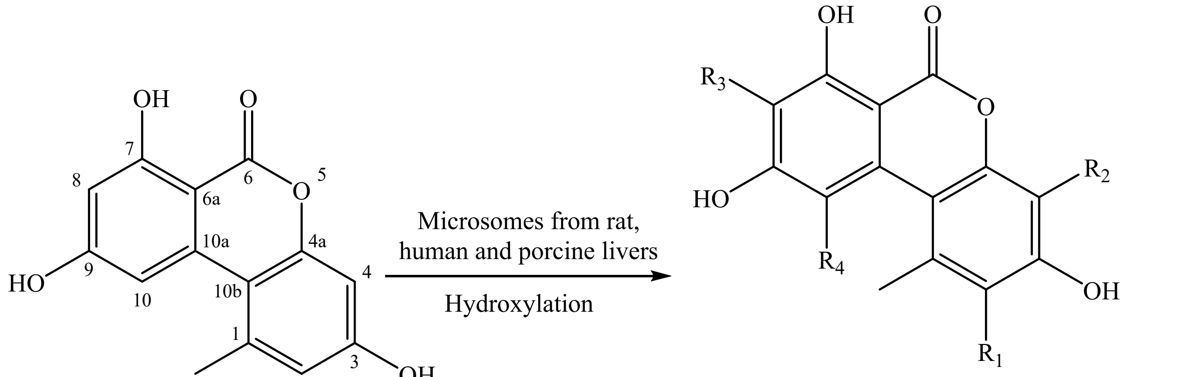

7. Alternariol (AOH)

9. 2-Hydroxy AOH, R_1=OH, R_2=H, R_3=H, R_4=H
10. 4-Hydroxy AOH, R_1=H, R_2=OH, R_3=H, R_4=H
11. 8-Hydroxy AOH, R_1=H, R_2=H, R_3=OH, R_4=H
12. 10-Hydroxy AOH, R_1=H, R_2=H, R_3=H, R_4=OH

Figure S3. Transformation of alternariol (**7**) with hydroxylation by the microsomes from rat, human and porcine livers [3].

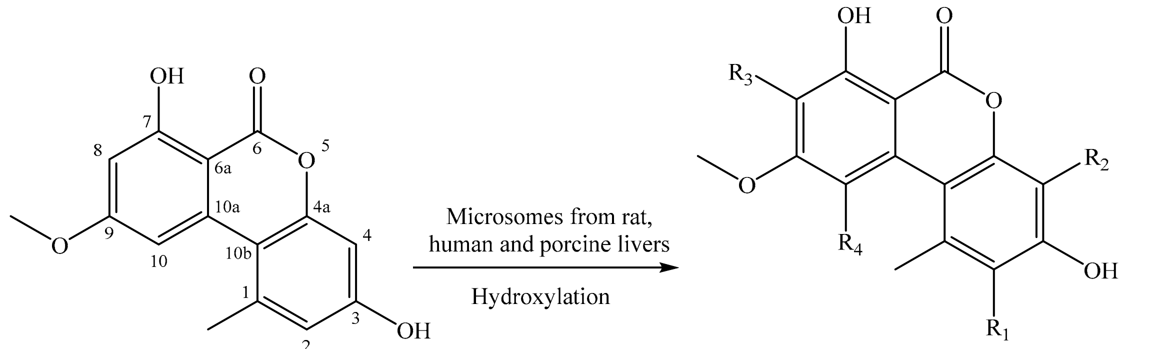

8. Alternariol 9-*O*-methyl ether (AME)

13. 2-Hydroxy AME, R_1=OH, R_2=H, R_3=H, R_4=H
14. 4-Hydroxy AME, R_1=H, R_2=OH, R_3=H, R_4=H
15. 8-Hydroxy AME, R_1=H, R_2=H, R_3=OH, R_4=H
16. 10-Hydroxy AME, R_1=H, R_2=H, R_3=H, R_4=OH

Figure S4. Transformation of alternariol 9-*O*-methyl ether (**8**) with hydroxylation by the microsomes from rat, human and porcine livers [3].

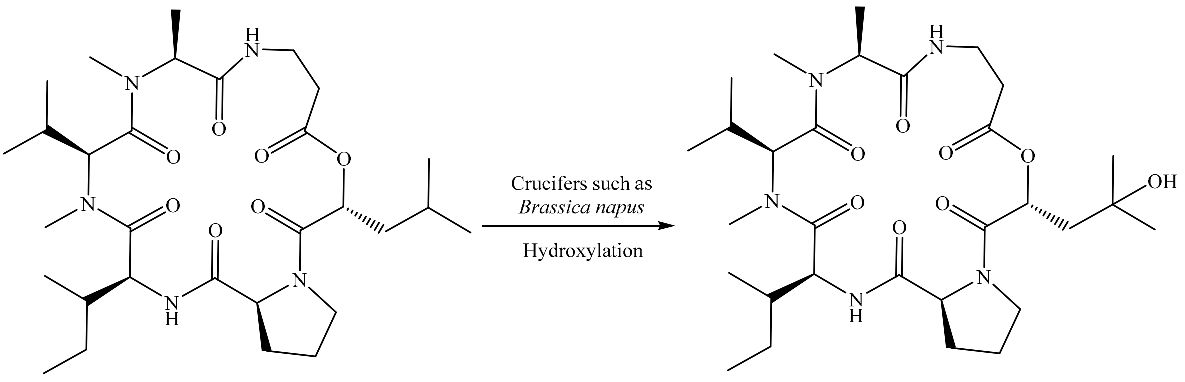

17. Destruxin B

18. Hydroxydestruxin B

Figure S5. Transformation of destruxin B (**17**) with hydroxylation by crucifers such as *Brassica napus* [4].

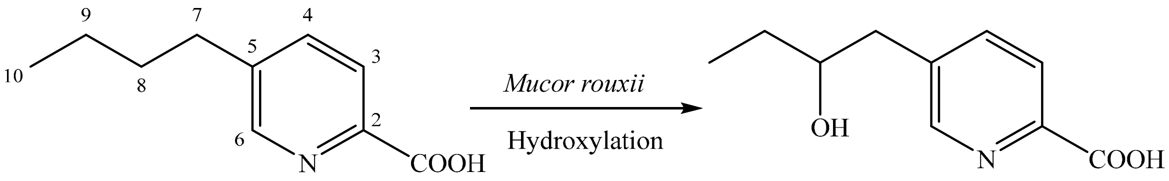

19. Fusaric acid

20. 8-Hydroxyfusaric acid

Figure S6. Transformation of fusaric acid (**19**) with hydroxylation by *Mucor rouxii* [5].

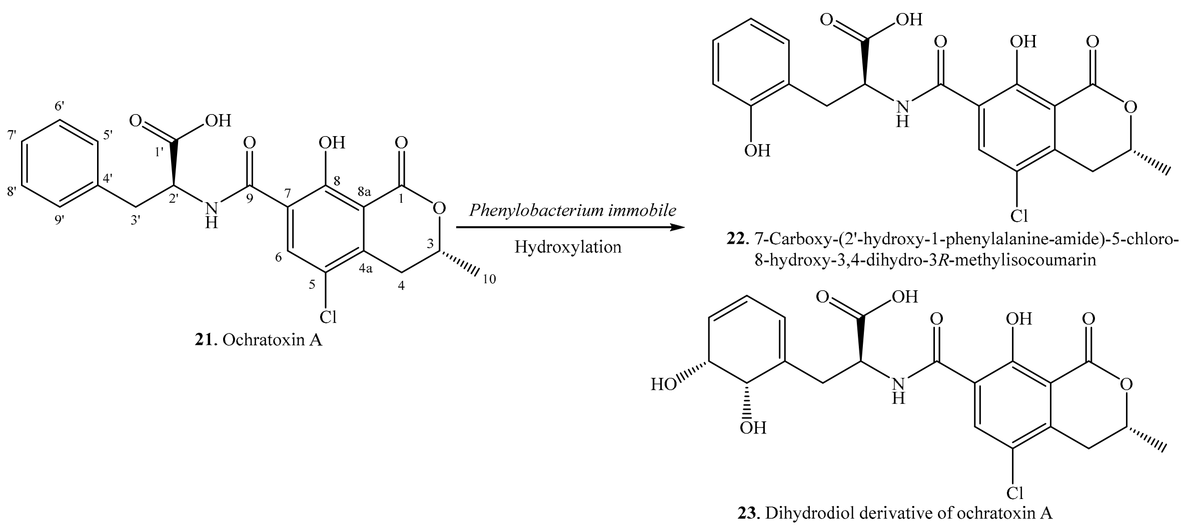

Figure S7. Transformation of ochratoxin A (**21**) with hydroxylation by *Phenylobacterium immobile* [6].

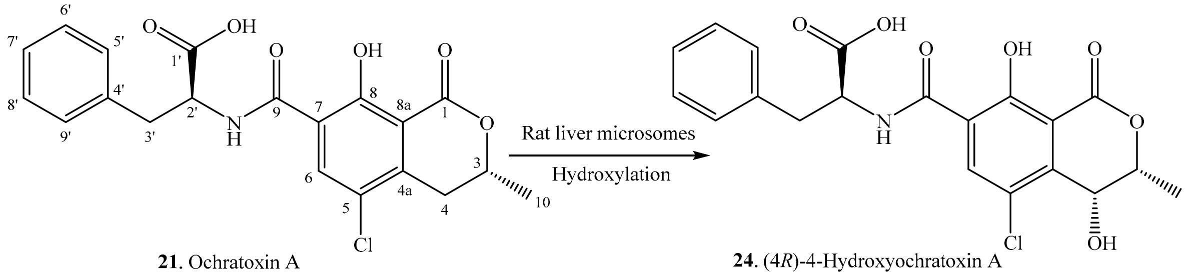

Figure S8. Transformation of ochratoxin A (**21**) with hydroxylation by rat liver microsomes [7].

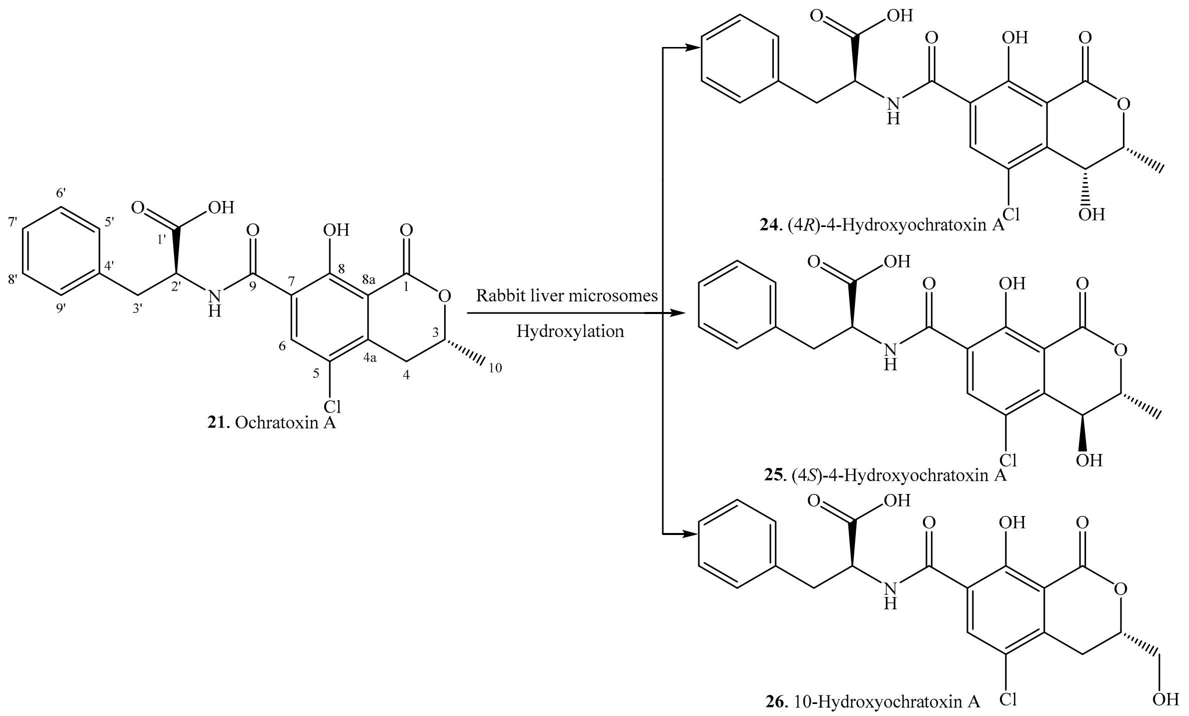

Figure S9. Transformation of ochratoxin A (**21**) with hydroxylation by rabbit liver microsomes [8].

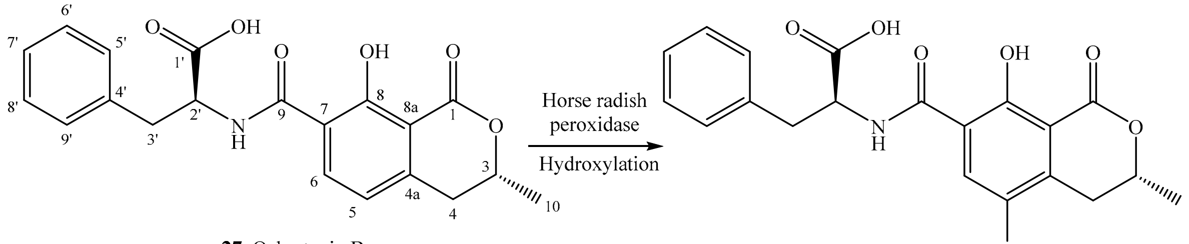

27. Ochratoxin B **28**. OTHQ

Figure S10. Transformation of ochratoxin B (**27**) with hydroxylation by horse radish peroxidase [9].

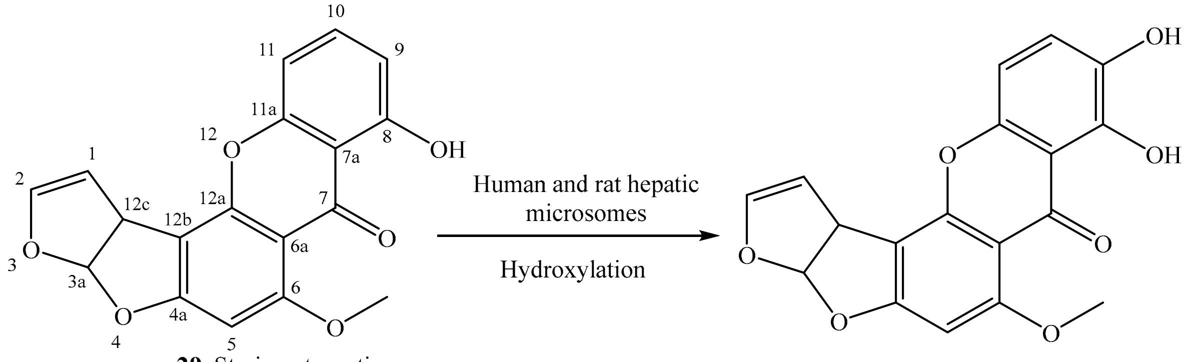

29. Sterigmatocystin **30**. 9-Hydroxy sterigmatocystin

Figure S11. Transformation of sterigmatocystin (**29**) with hydroxylation by human and rat hepatic microsomes [10].

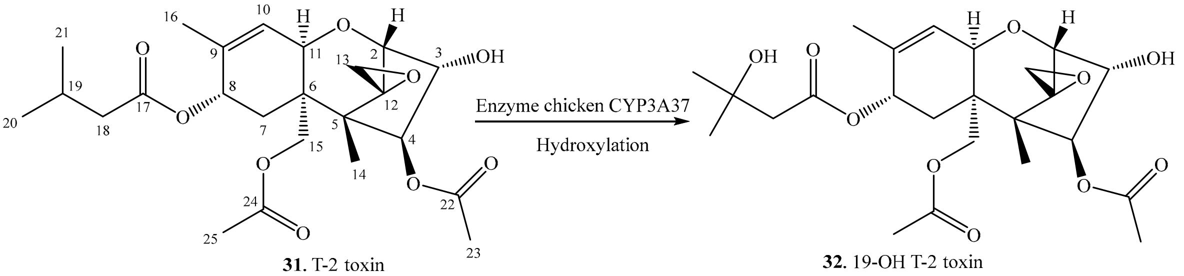

31. T-2 toxin **32**. 19-OH T-2 toxin

Figure S12. Transformation of T-2 toxin (**31**) with hydroxylation by chicken CYP3A37 [11].

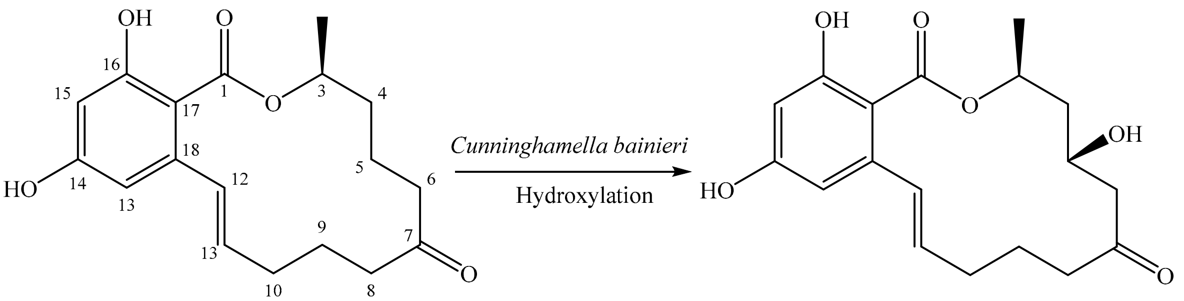

33. Zearalenone (ZEN) **34**. (5*S*)-5-Hydroxy ZEN

Figure S13. Transformation of zearalenone (**33**) with hydroxylation by *Cunninghamella bainieri* [12].

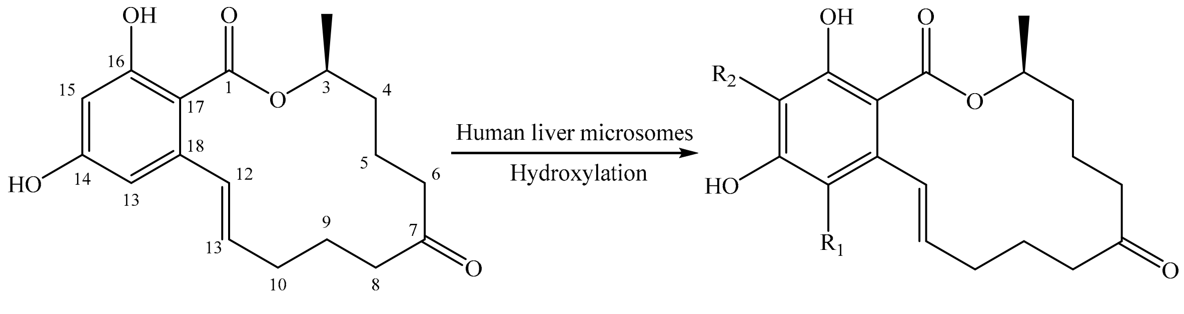

33. Zearalenone (ZEN)

35. 13-Hydroxy ZEN, R_1=OH, R_2=H
36. 15-Hydroxy ZEN, R_1=H, R_2=OH

Figure S14. Transformation of zearalenone (**33**) with hydroxylation by human liver microsomes [13].

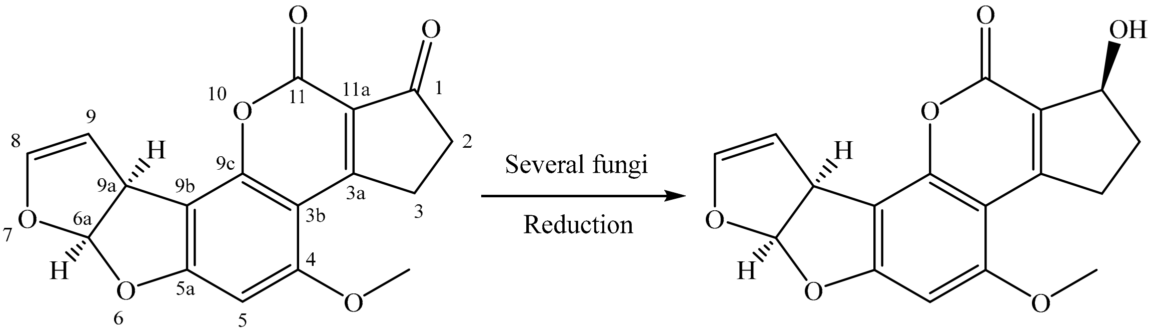

1. Aflatoxin B_1

37. Aflatoxicol

Figure S15. Transformation of aflatoxin B1 (**1**) with reduction by several fungi [14].

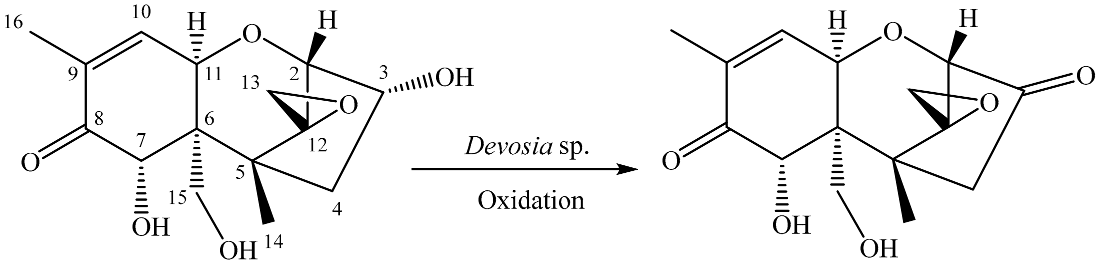

38. Deoxynivalenol (DON)

39. 3-Keto-DON

Figure S16. Transformation of deoxynivalenol (**38**) with oxidation by *Devosia* sp. [15].

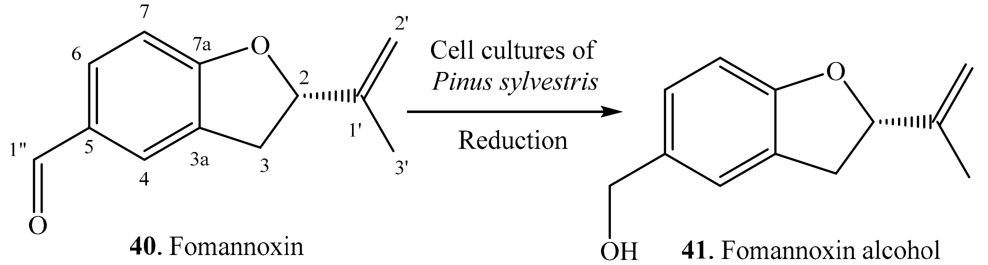

40. Fomannoxin

41. Fomannoxin alcohol

Figure S17. Transformation of fomannoxin (**40**) with reduction by cell cultures of *Pinus sylvestris* [16].

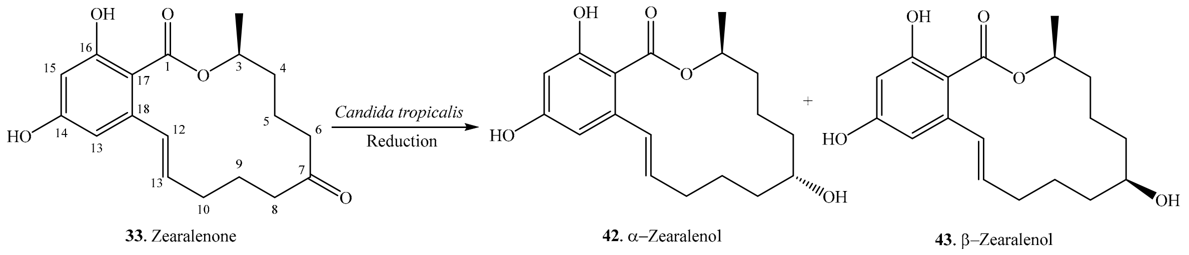

Figure S18. Transformation of zearalenone (**33**) with reduction by *Candida tropicalis* [17].

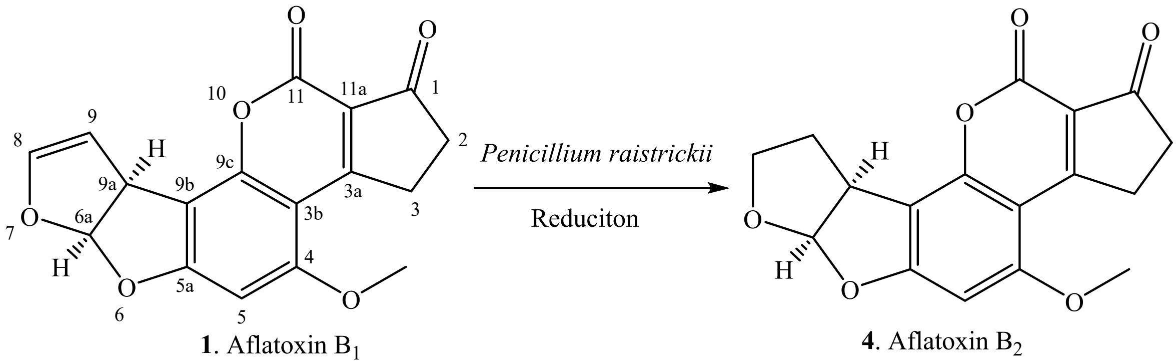

Figure S19. Transformation of aflatoxin B1 (**1**) with reduction by the fungus *Penicillium raistrickii* [2].

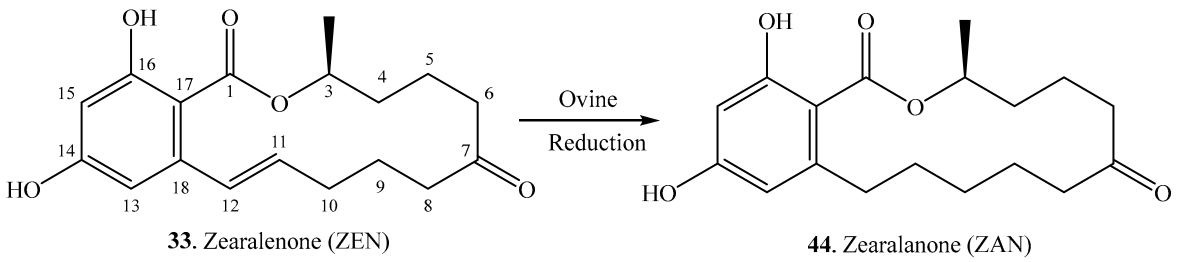

Figure S20. Transformation of zearalenone (**33**) with reduction in ovine [18].

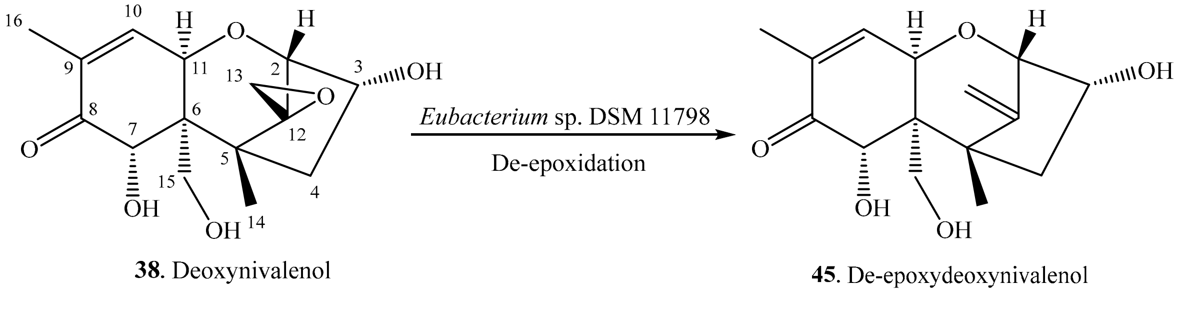

Figure S21. Transformation of deoxynivalenol (**38**) with de-epoxidaton by *Eubacterium* sp. DSM 11798 [19].

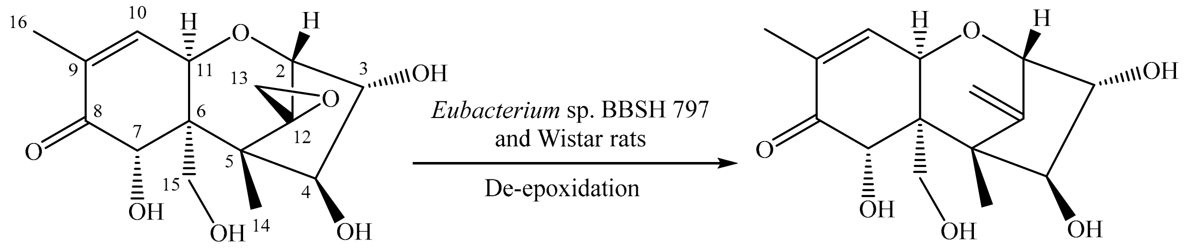

46. Nivalenol **47**. De-epoxynivalenol

Figure S22. Transformation of nivalenol (**46**) with de-epoxidation by the bacterium *Eubacterium* sp. BBSH 797 [20] and Wistar rats [21].

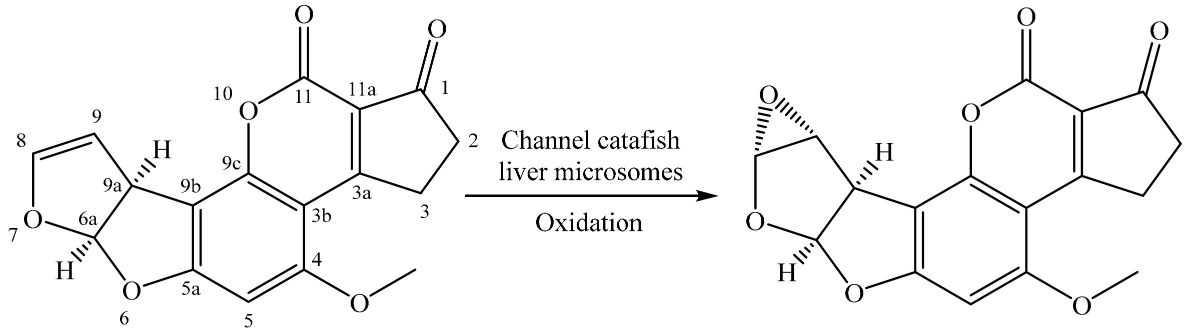

1. Aflatoxin B_1 **48**. Aflatoxin B_1-8,9-epoxide

Figure S23. Transformation of aflatoxin B_1 (**1**) with oxidation by channel catafish microsomes [22].

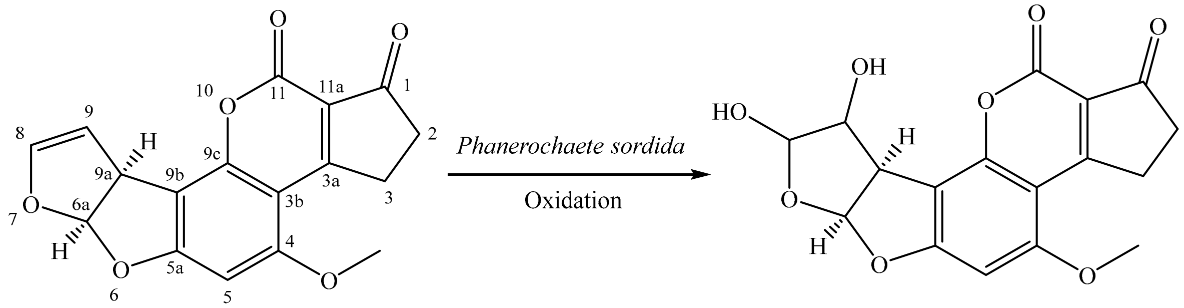

1. Aflatoxin B_1 **49**. Aflatoxin B_1-8,9-dihydrodiol

Figure S24. Transformation of aflatoxin B_1 (**1**) with oxidation by *Phanerochaete sordida* YK-624 [23].

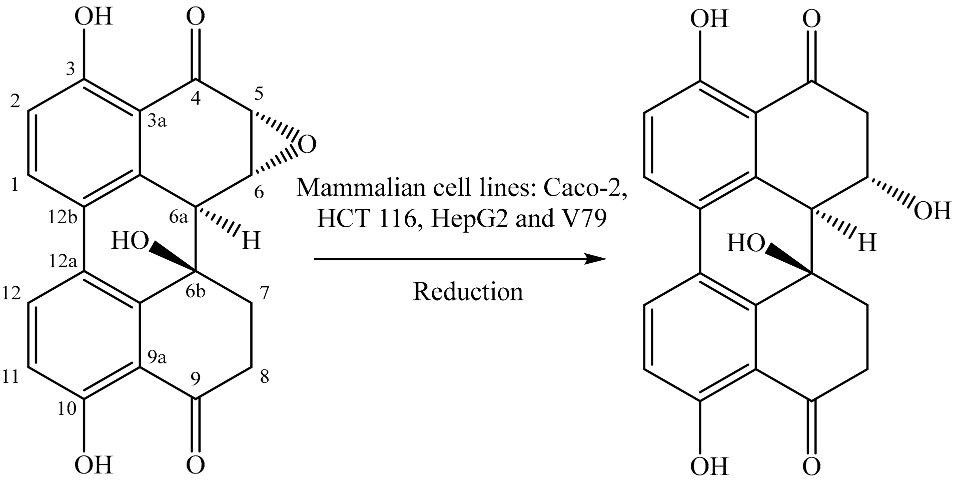

50. Altertoxin II (ATX II) **52**. Altertoxin I (ATX I)

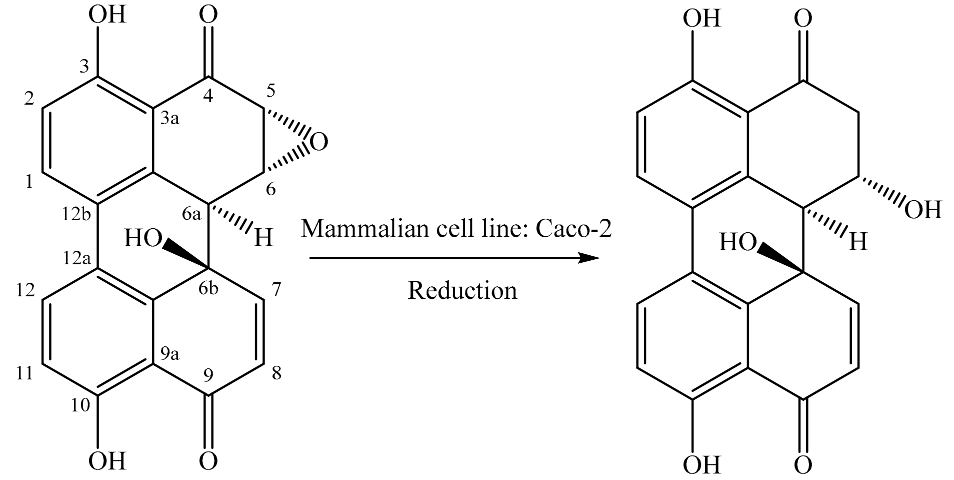

51. Stemphyltoxin III (STTX III) **53**. Alteichin (ALTCH)

Figure S25. Transformation of altertoxin II (**50**) and stemphyltoxin III (**51**) with reduction by mammalian cells [24].

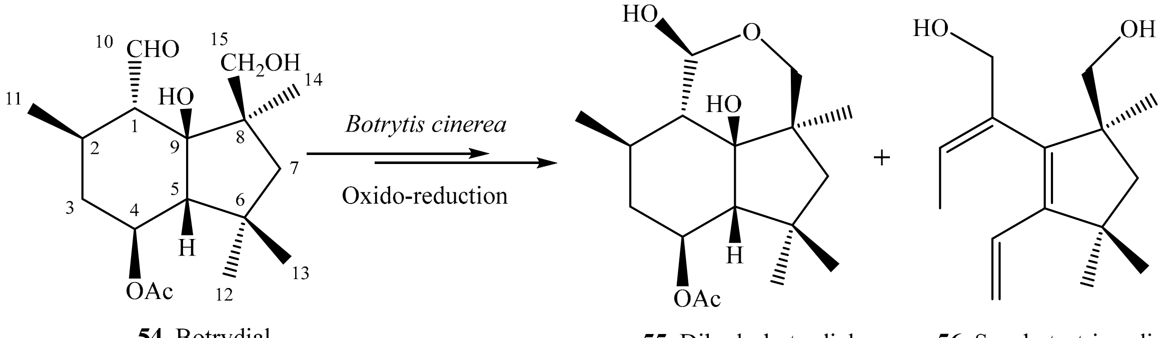

54. Botrydial **55**. Dihydrobotrydial **56**. Secobotrytrienediol

Figure S26. Transformation of botrydial (**54**) with oxido-reductions by *Botrytis cinerea* [25].

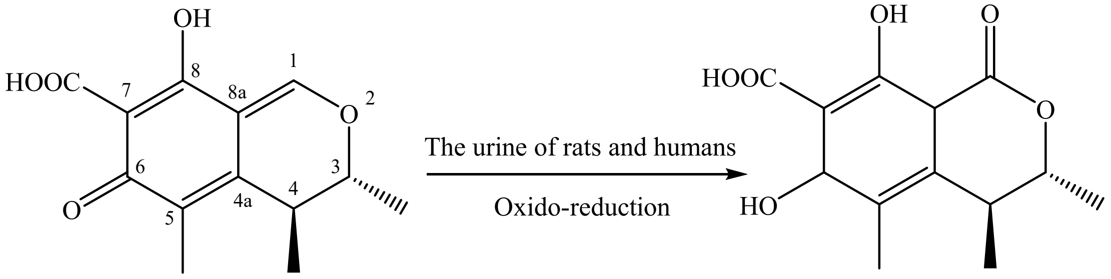

57. Citrinin **58**. Dihyrocitrinone

Figure S27. Transformation of citrinin (**57**) with oxido-reduction in the urine of rats and humans [26,27].

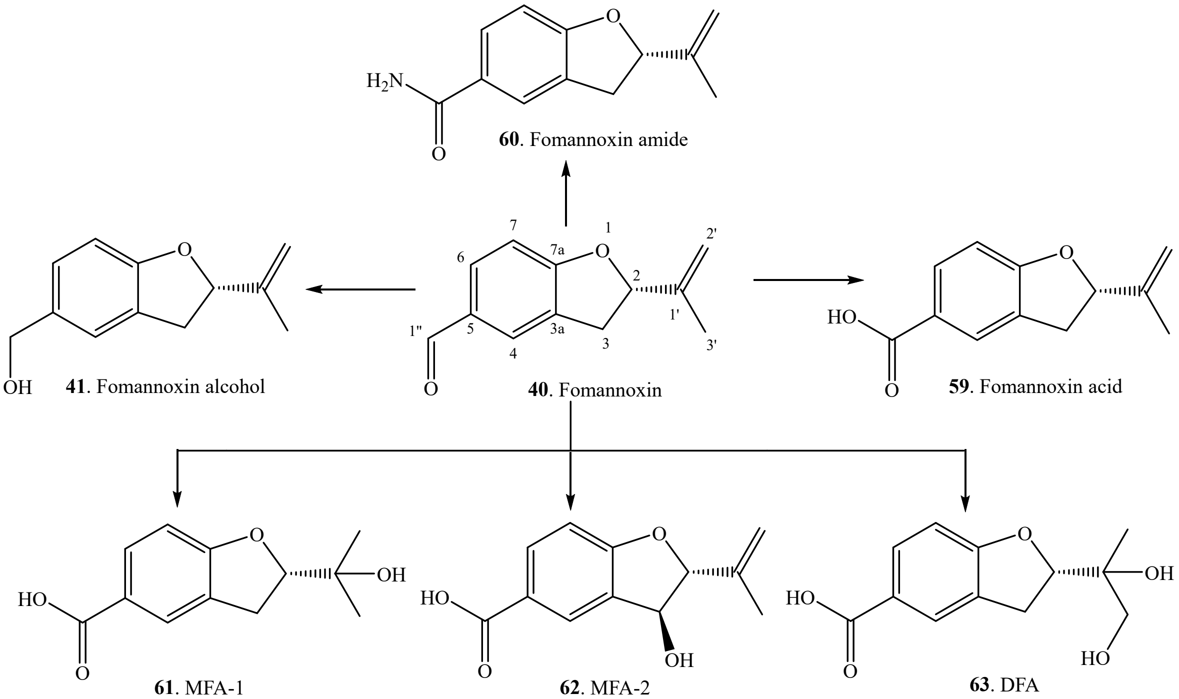

Figure S28. Transformation of fomannoxin (**40**) with oxido-reduction by rhizosphere-associated *Streptomyces* sp. AcH 505 [28].

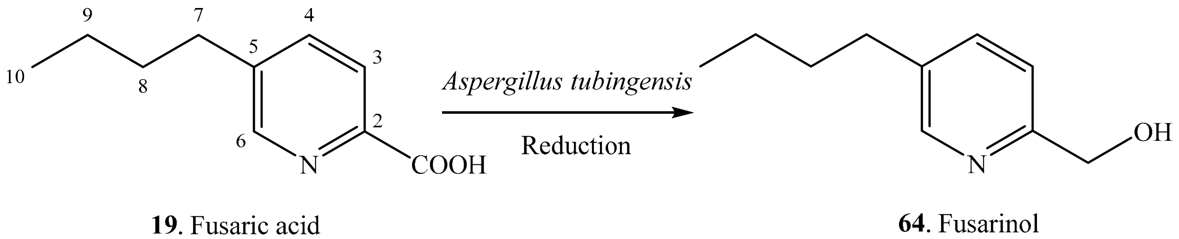

19. Fusaric acid **64**. Fusarinol

Figure S29. Transformation of fusaric acid (**19**) with reduction by *Aspergillus tubingensis* [29].

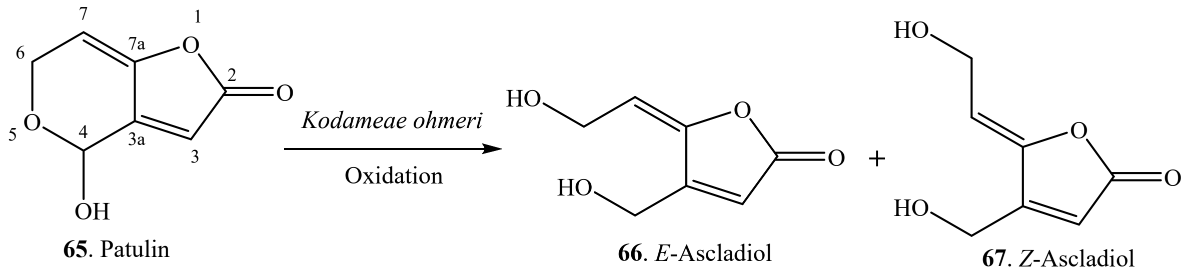

Figure S30. Transformation of patulin (**65**) with reduction by the yeast *Kodameae ohmeri* [30].

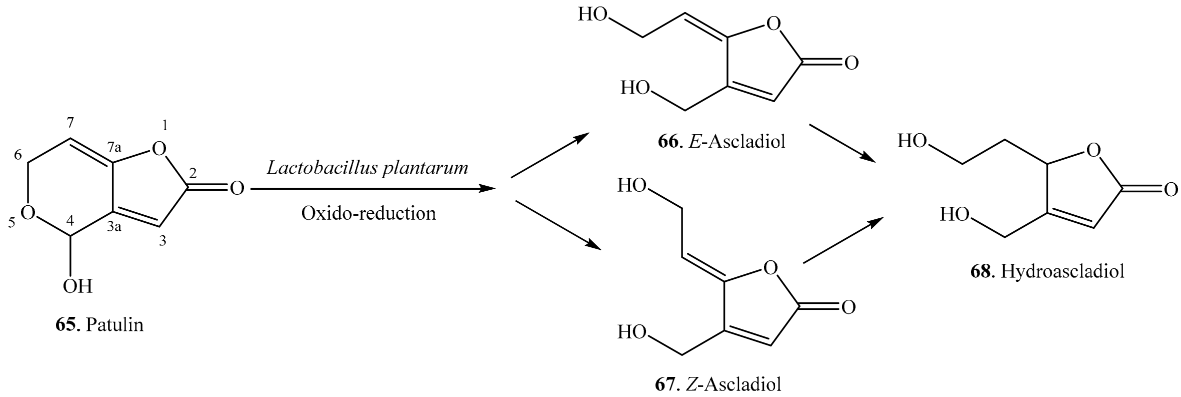

Figure S31. Transformation of patulin (**65**) with reduction by the bacterium *Lactobacillus plantarum* [31].

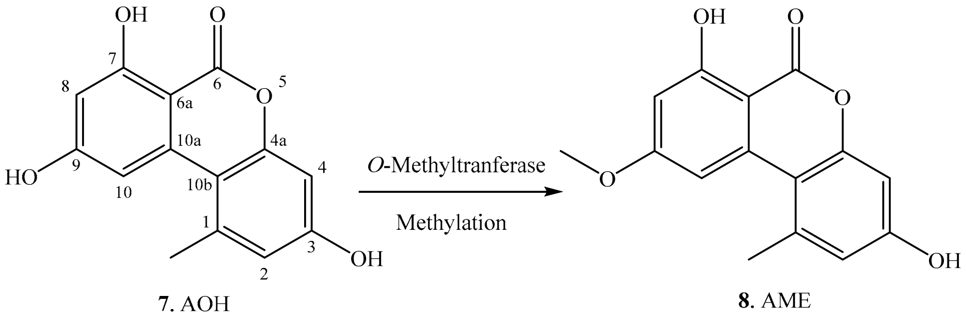

Figure S32. Transformation of alternariol (**7**) with methylation by *O*-methyltransferase [32].

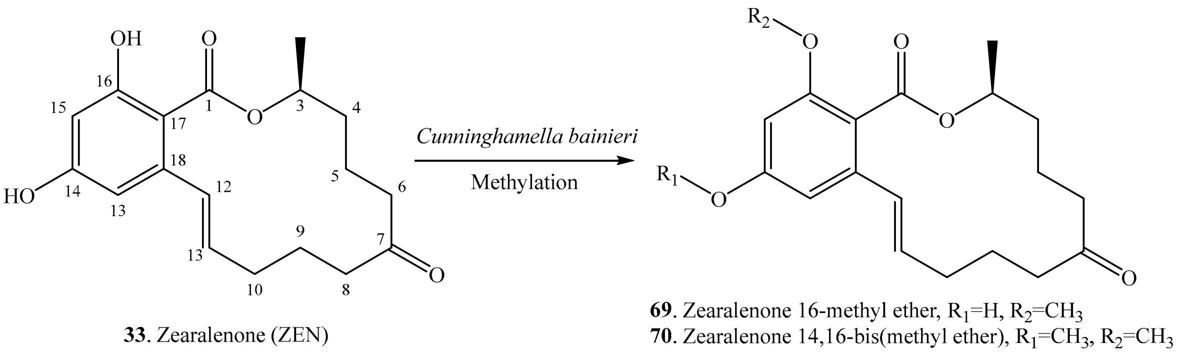

Figure S33. Transformation of zearalenone (**33**) with methylation by *Cunninghamella bainieri* [12].

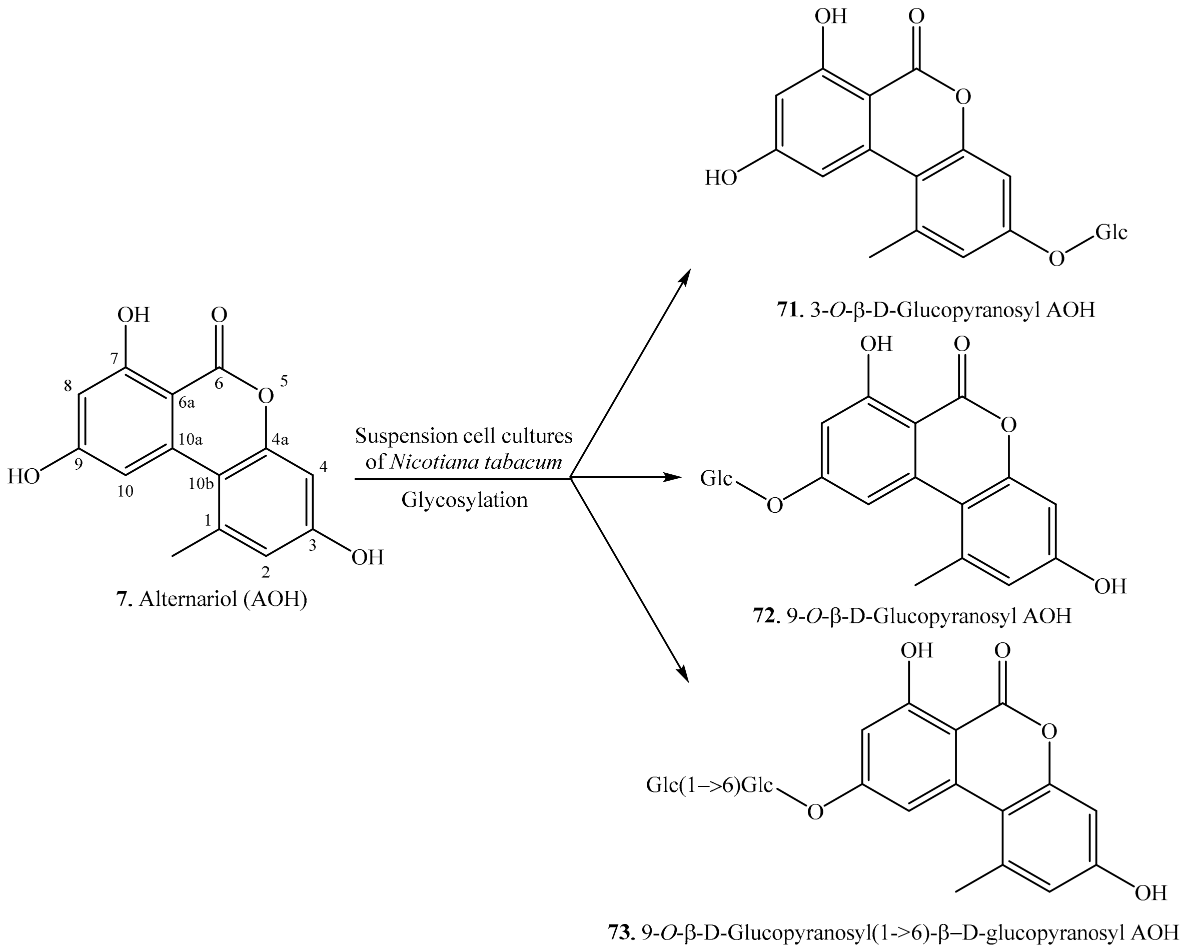

Figure S34. Transformation of alternariol (**7**) with glycosylation by suspension cell cultures of *Nicotiana batacum* [33].

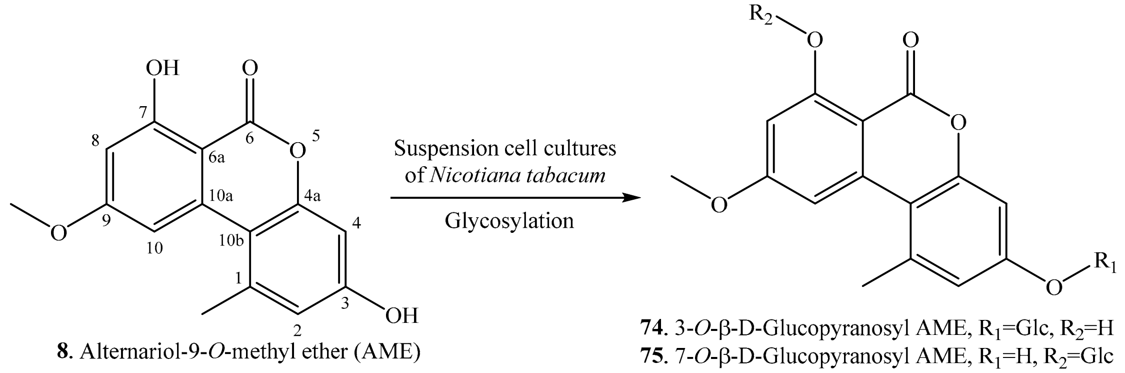

Figure S35. Transformation of alternariol 9-*O*-methyl ether (**8**) with glycosylation by suspension cell cultures of *Nicotiana batacum* [33].

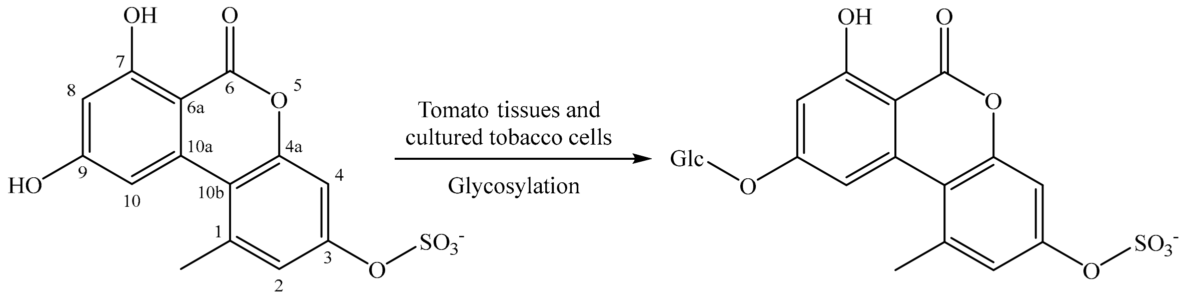

76. AOH 3-sulfate

79. AOH 3-sulfate-9-*O*-glucoside

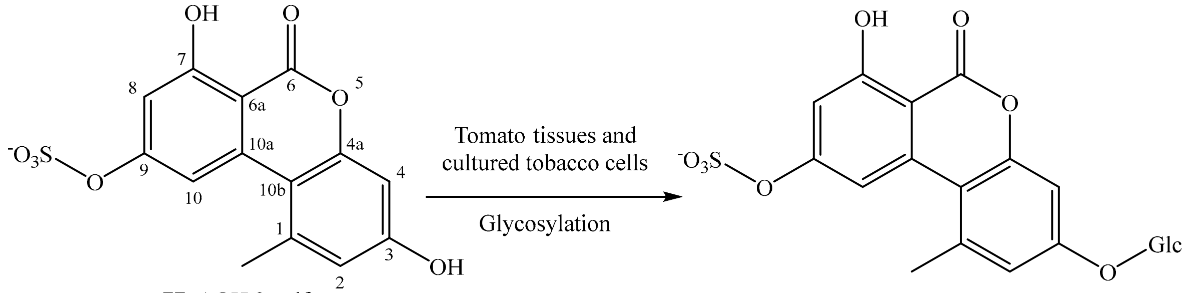

77. AOH 9-sulfate

80. AOH 9-sulfate-3-*O*-glucoside

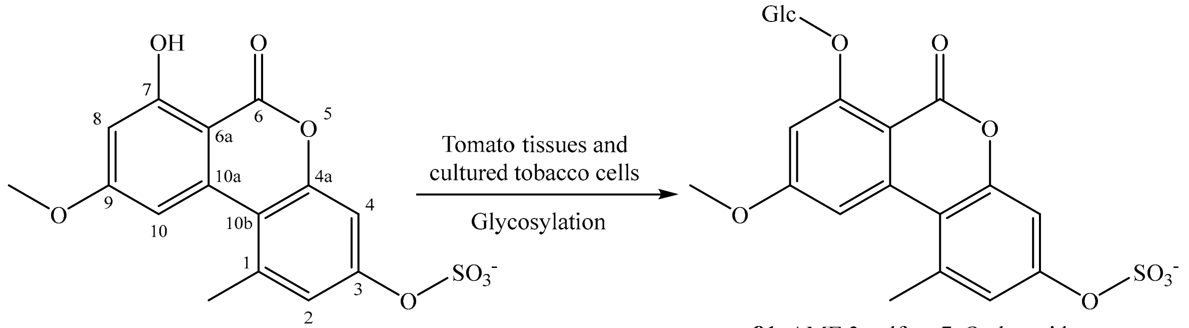

78. AME 3-sulfate

81. AME 3-sulfate-7-*O*-glucoside

Figure S36. Transformation of alternariol 3-sulfate (**76**), alternariol 9-sulfate (**77**), and alternariol 9-*O*-methyl ether 3-sulfate (**78**) by tomato tissues and cultured tobacco cells [34].

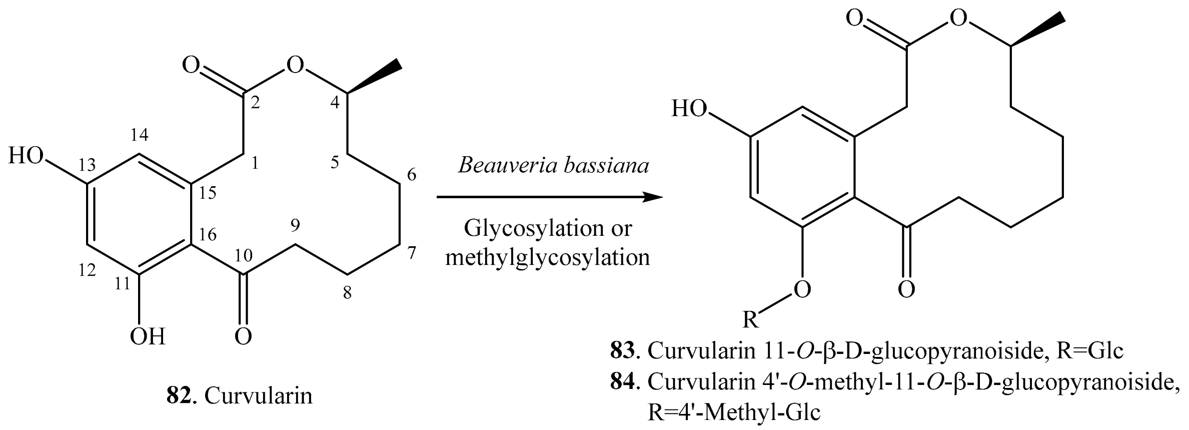

82. Curvularin

83. Curvularin 11-*O*-β-D-glucopyranoiside, R=Glc

84. Curvularin 4'-*O*-methyl-11-*O*-β-D-glucopyranoiside, R=4'-Methyl-Glc

Figure S37. Transformation of curvularin (**82**) with glycosylation or methylglycosylation by *Beauveria bassiana* [35].

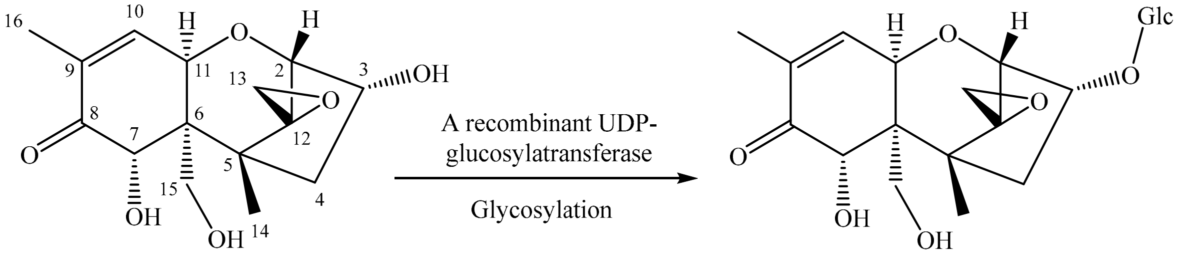

38. Deoxynivalenol (DON)

85. DON 3-*O*-β–D-glucoside (D3G)

Figure S38. Transformation of deoxynivalenol (**38**) with glycosylation by a recombinant UDP-glucosyltransferase from rice [36].

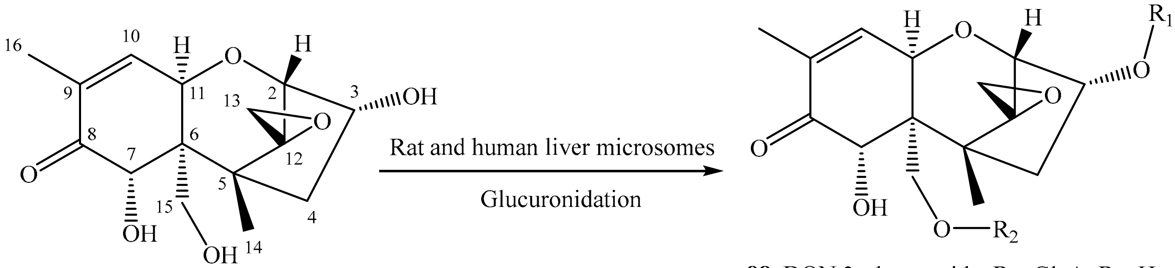

38. Deoxynivalenol (DON)

88. DON 3-glucuronide, R_1=GlcA, R_2=H
89. DON 15-glucuronide, R_1=H, R_2=GlcA

Figure S39. Transformation of deoxynivalenol (DON, **38**) with glucuronidation by rat and human liver microsomes [37,38].

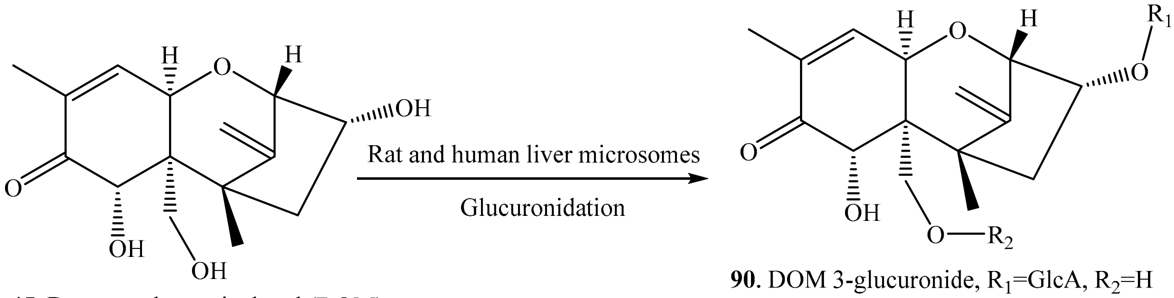

45. Deepoxy-deoxynivalenol (DOM)

90. DOM 3-glucuronide, R_1=GlcA, R_2=H
91. DOM 15-glucuronide, R_1=H, R_2=GlcA

Figure S40. Transformation of deepoxy-deoxynivalenol (DOM, **45**) with glucuronidation by rat and humam liver microsomes [37,38].

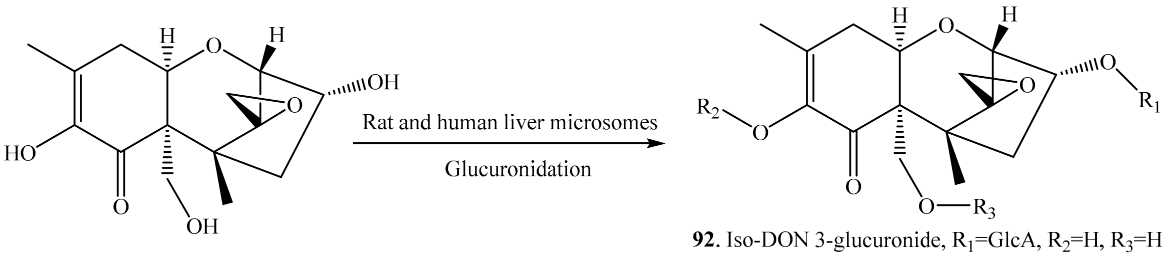

86. Iso-deoxynivalenol (Iso-DON)

92. Iso-DON 3-glucuronide, R_1=GlcA, R_2=H, R_3=H
93. Iso-DON 8-glucuronide, R_1=H, R_2=GlcA, R_3=H
94. Iso-DON 15-glucuronide, R_1=H, R_2=H, R_3=GlcA

Figure S41. Transformation of iso-deoxynivalenol (iso-DON, **86**) with glucuronidation by rat and human liver microsomes [37,38].

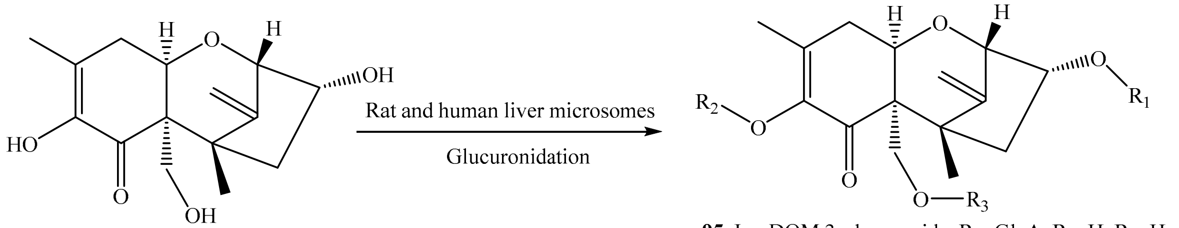

87. Iso-deepoxy-deoxynivalenol (Iso-DOM)

95. Iso-DOM 3-glucuronide, R_1=GlcA, R_2=H, R_3=H
96. Iso-DOM 8-glucuronide, R_1=H, R_2=GlcA, R_3=H
97. Iso-DOM 15-glucuronide, R_1=H, R_2=H, R_3=GlcA

Figure S42. Transformation of iso-deepoxy-deoxynivalenol (iso-DOM, **87**) with glucuronidation by rat and humam liver microsomes [37,38].

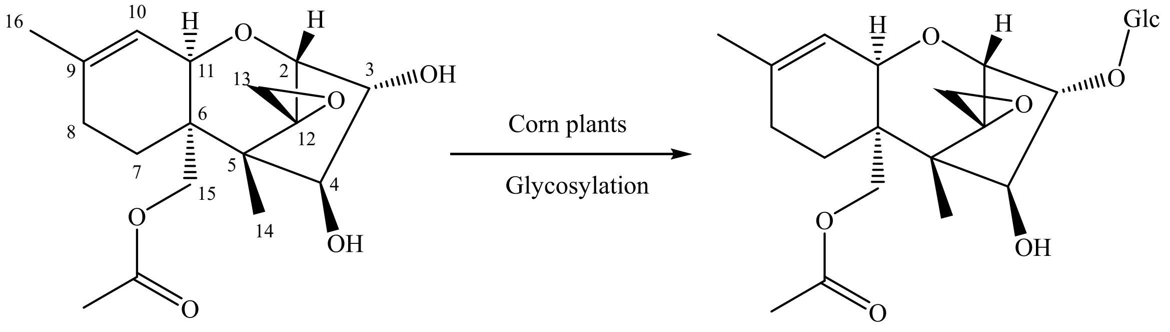

98. 15-Monoacetoxyscirpenol (15-MAS) **100**. 15-MAS 3-glucoside

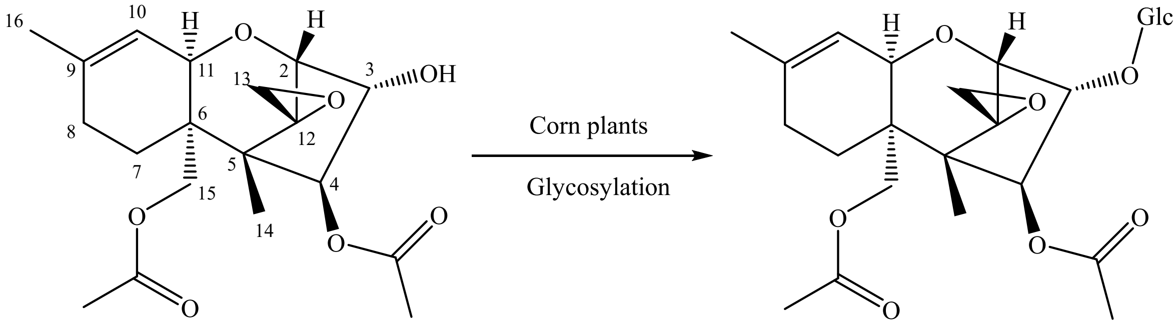

99. 4,15-Diacetoxyscirpenol (4,15-DAS) **101**. 4,15-DAS 3-glucoside

Figure S43. Transformation of 15-monoacetoxyscirpenol (**98**) and 4,15-diacetoxyscirpenol (**99**) with glycosylation respectively by corn plants [39].

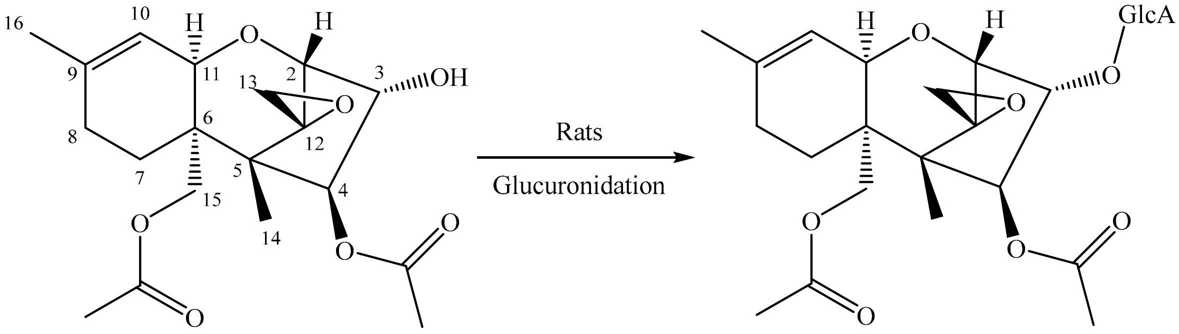

99. 4,15-Diacetoxyscirpenol (4,15-DAS) **102**. 4,15-DAS 3-glucuronide

Figure S44. Transformation of 4,15-diacetoxyscirpenol (**99**) with glucuronidation in rats [40].

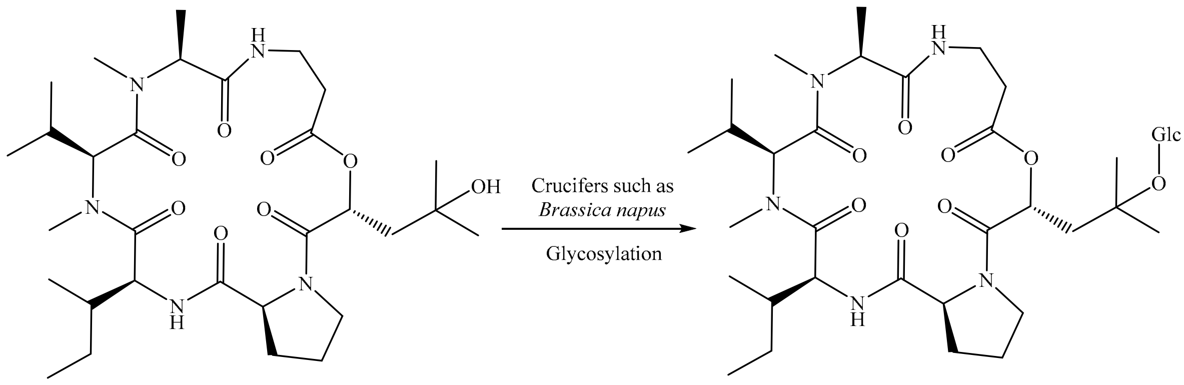

103. Hydroxydestruxin B

104. Glucosyl hydroxydestruxin B

Figure S45. Transformation of hydroxydestruxin B (**103**) with glycosylation by crucifers such as *Brassica napus* [4].

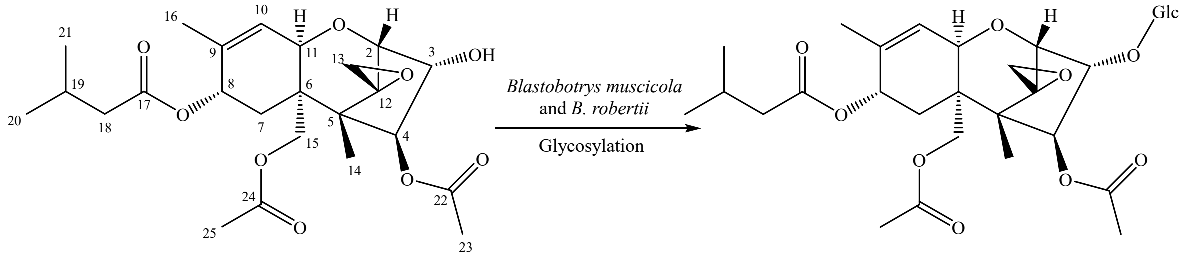

31. T-2 toxin

105. T-2 toxin 3-*O*-α-D-glucoside

Figure S46. Transformation of T-2 toxin (**31**) with glycosylation by *Blastobotrys muscicola* and *B. robertii* [41].

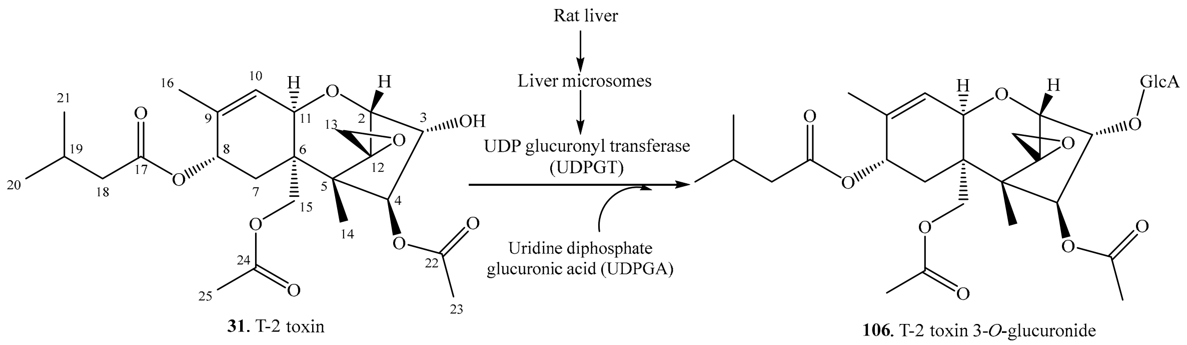

31. T-2 toxin

106. T-2 toxin 3-*O*-glucuronide

Figure S47. Transformation of T-2 toxin (**31**) with glucuronidation by rat liver microsomes [42].

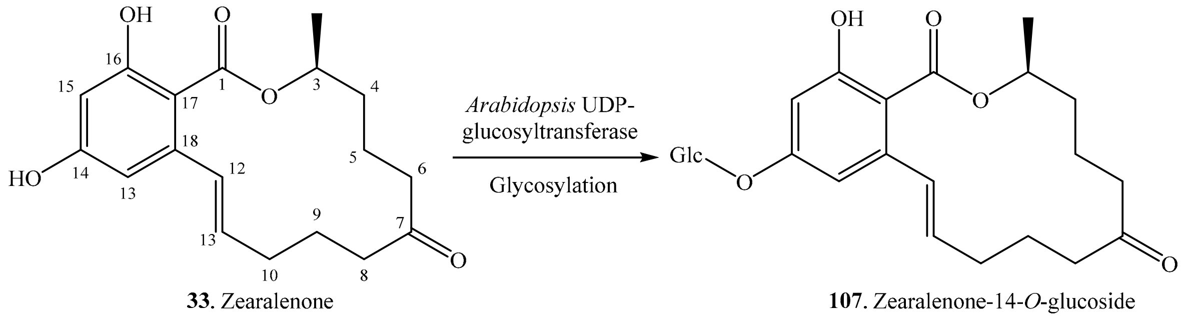

33. Zearalenone

107. Zearalenone-14-*O*-glucoside

Figure S48. Transformation of zeralenone (**33**) by *Arabidopsis* UDP-glucosyltransferases expressed in *Saccharomyces cerevisiae* [43].

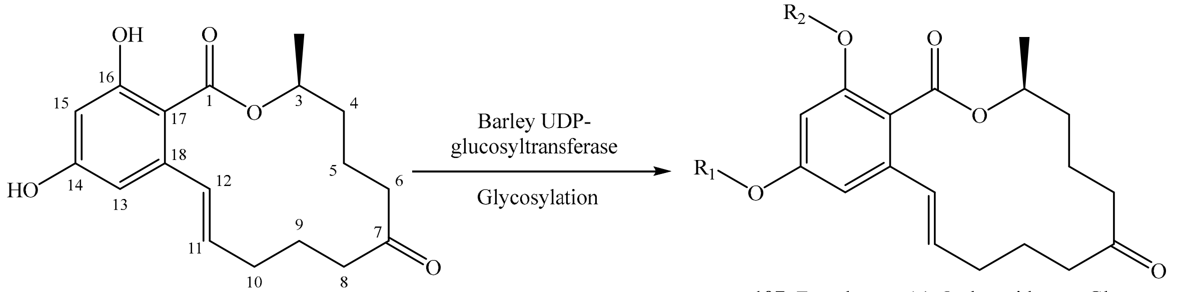

33. Zearalenone

107. Zearalenone 14-*O*-glucoside, R_1=Glc, R_2=H
108. Zearalenone 16-*O*-glucoside, R_1=H, R_2=Glc

Figure S49. Transformation of zeralenone (**33**) by barley UDP-glucosyltransferases expressed in *Saccharomyces cerevisiae* [44].

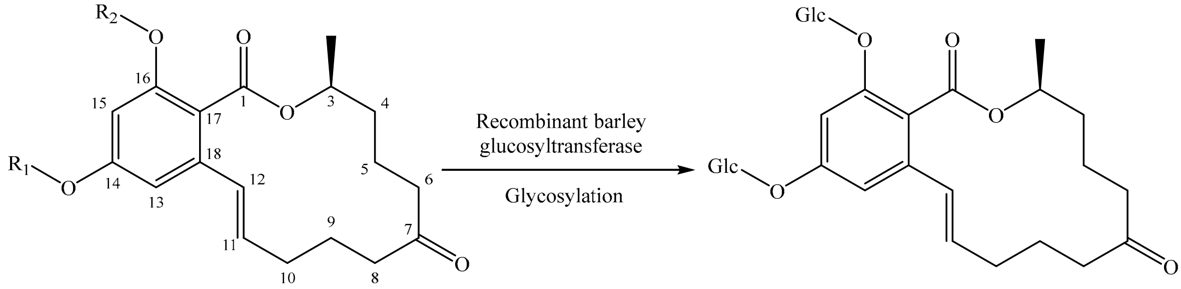

107. Zearalenone 14-*O*-glucoside, R_1=Glc, R_2=H
108. Zearalenone 16-*O*-glucoside, R_1=H, R_2=Glc

109. Zearalenone 14,16-di-glucoside

Figure S50. Transformation of zeralenone 14-*O*-glucoside (**107**) and zeralenone 16-*O*-glucoside (**108**) by the recombinant barley glucosyltransferases [45].

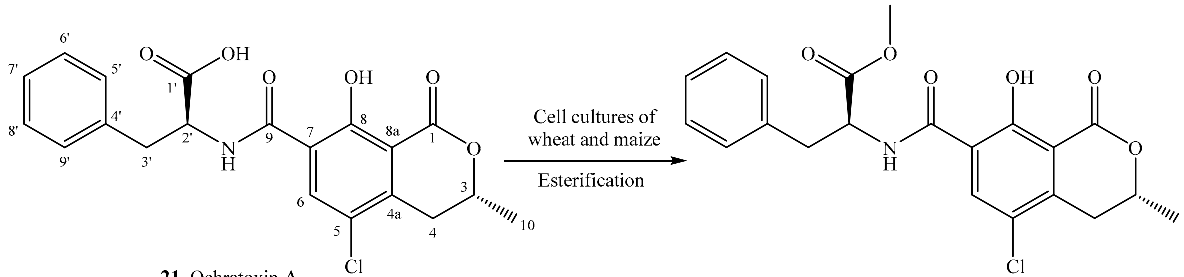

21. Ochratoxin A

110. Ochratoxin A methyl ester

Figure S51. Transformation of ochratoxin A (**21**) with esterification by the cell cultures of wheat and maize [46].

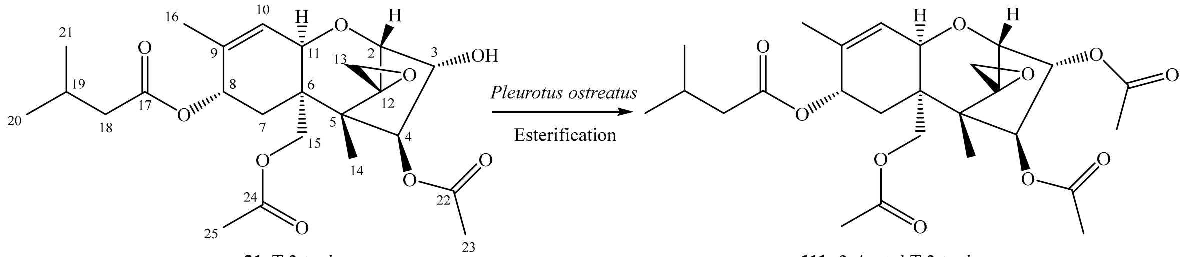

31. T-2 toxin

111. 3-Acetyl T-2 toxin

Figure S52. Transformation of T-2 toxin (**31**) with esterification (acetylation) by *Pleurotus ostreatus* [47].

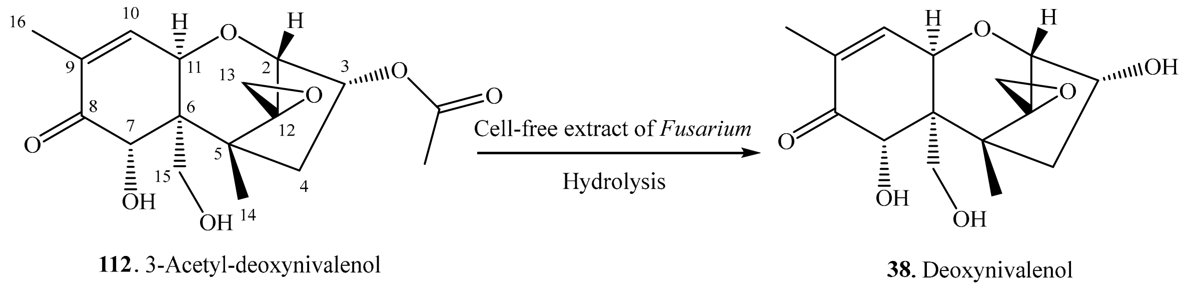

Figure S53. Transformation of 3-acetyl-deoxynivalenol (**112**) with hydrolysis (deacetylation) by cell-free extract of *Fusarium* sp. [48].

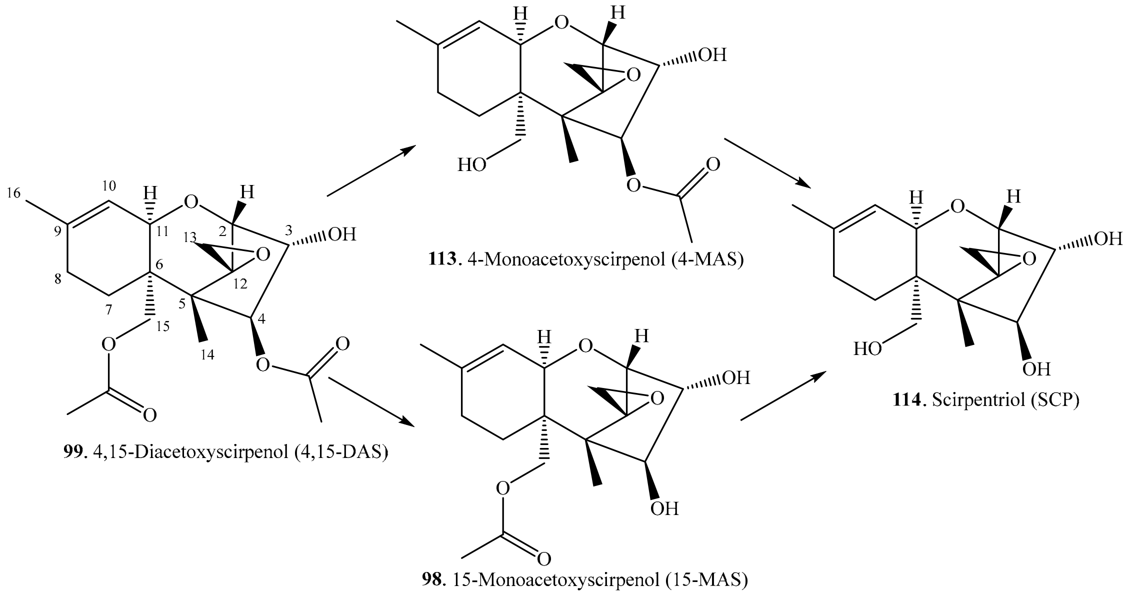

Figure S54. Transformation of 4,15-diacetoxyscirpenol (**99**) with hydrolysis (deacetylation) in rats [40].

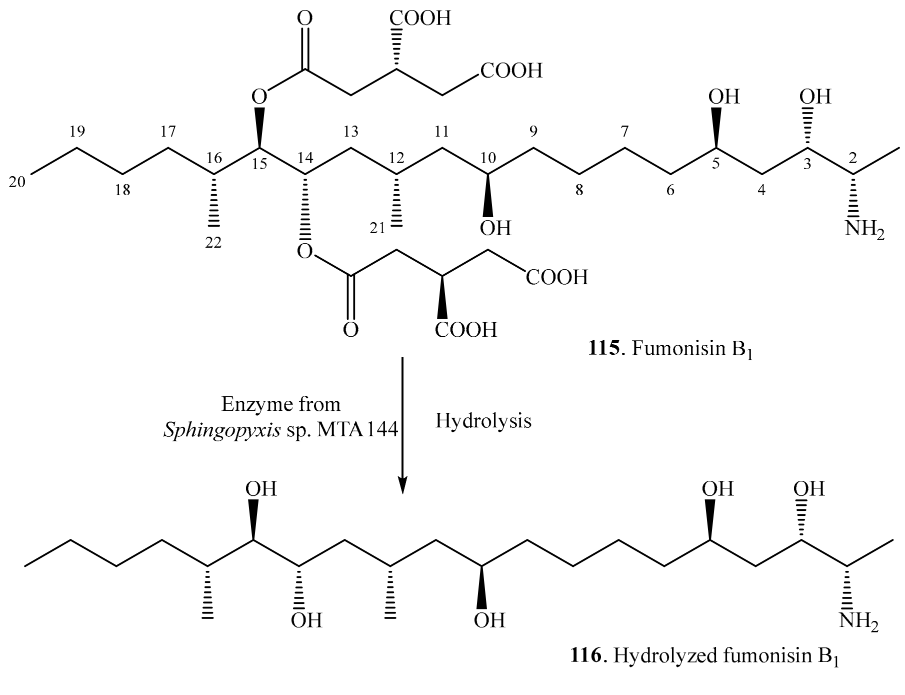

Figure S55. Transformation of fumonisin B1 (**115**) with hydrolysis by the enzyme from *Sphingopyxis* sp. MTA144 [49].

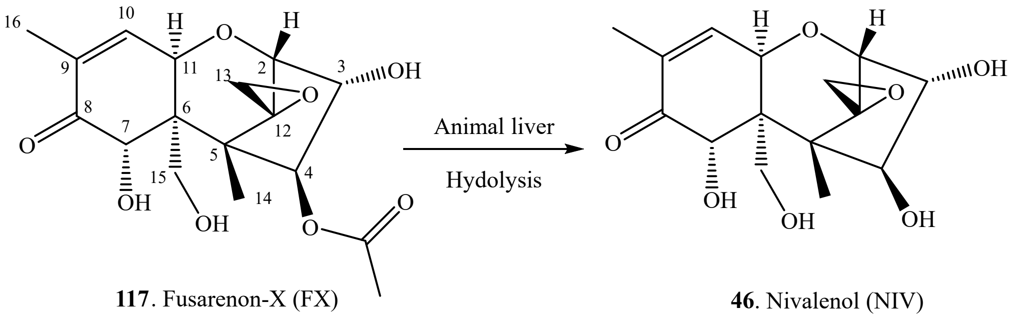

Figure S56. Transformation of fusarenon-X (**117**) with hydrolysis (deacetylation) in animal liver [50,51].

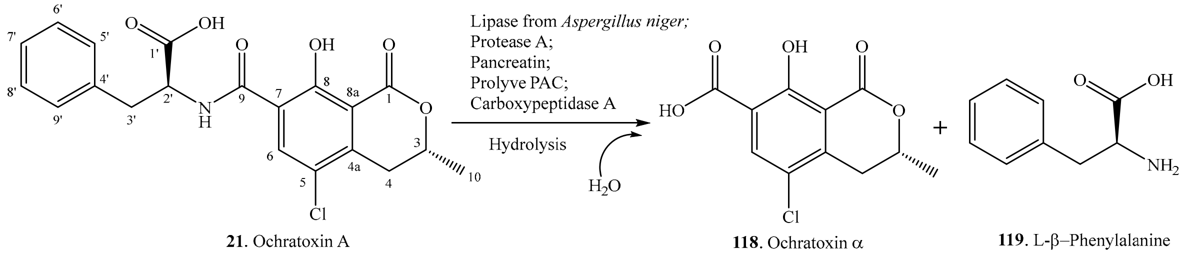

Figure S57. Transformation of ochratoxin A (**21**) with hydrolysis by enzymes [52–54].

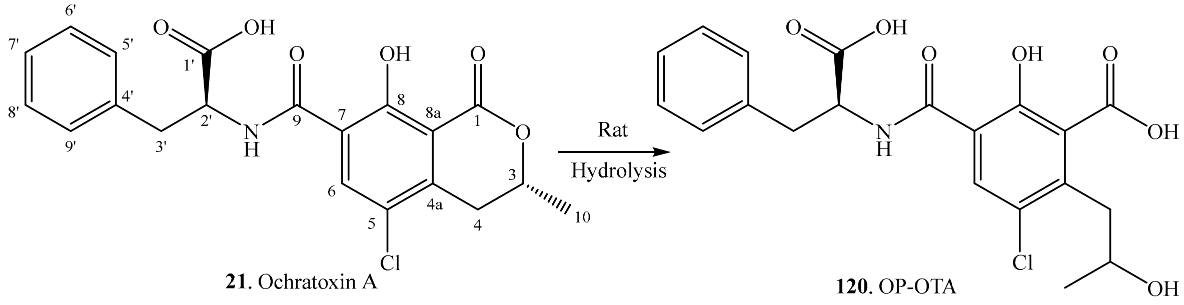

Figure S58. Transformation of ochratoxin A (**21**) with hydrolysis in rats [55].

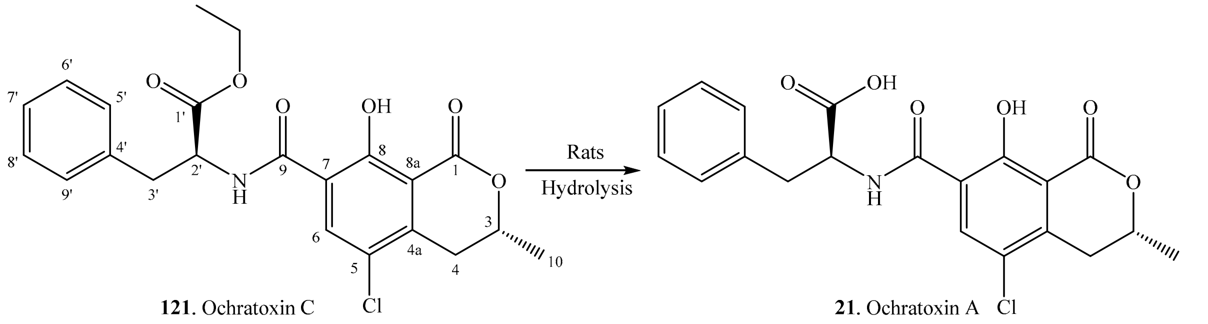

Figure S59. Transformation of ochratoxin C (**121**) with hydrolysis in rats [56].

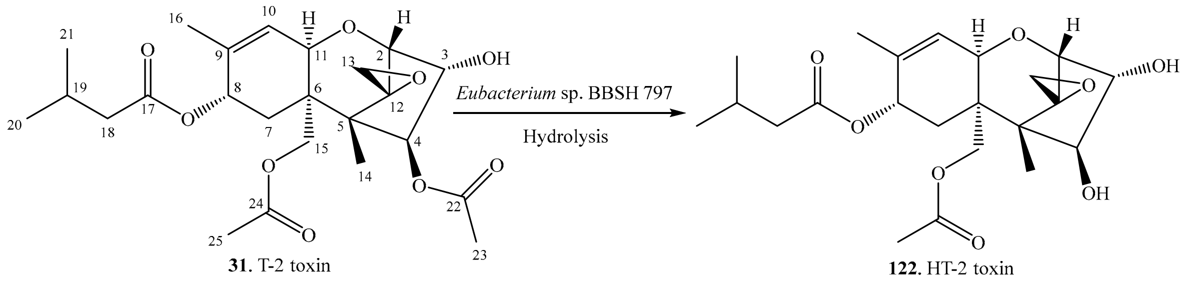

Figure S60. Transformation of T-2 toxin (**31**) with deacetylation by *Eubacterium* sp. 797 [57].

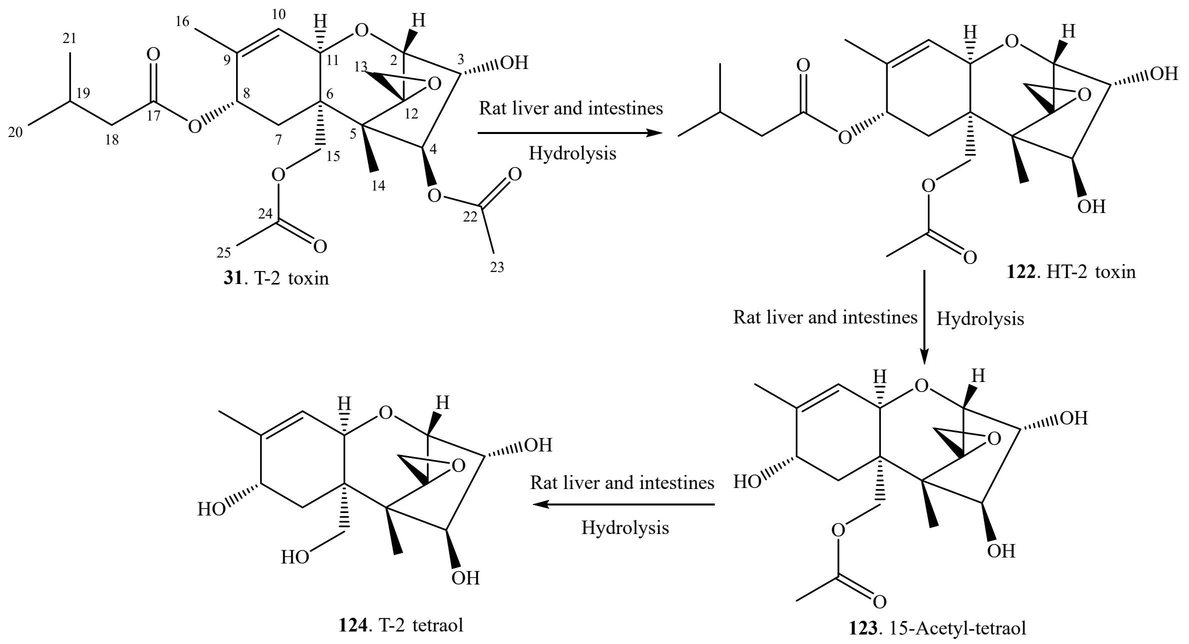

Figure S61. Transformation of T-2 toxin (**31**) with multi-step hydrolysis in rat liver and intestines [58].

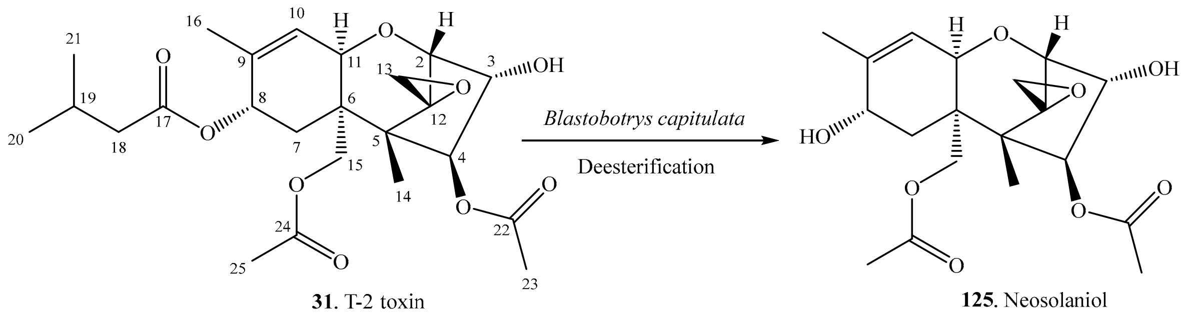

Figure S62. Transformation of T-2 toxin (**31**) with deesterification by the fungus *Blastobotrys capitulata* [41].

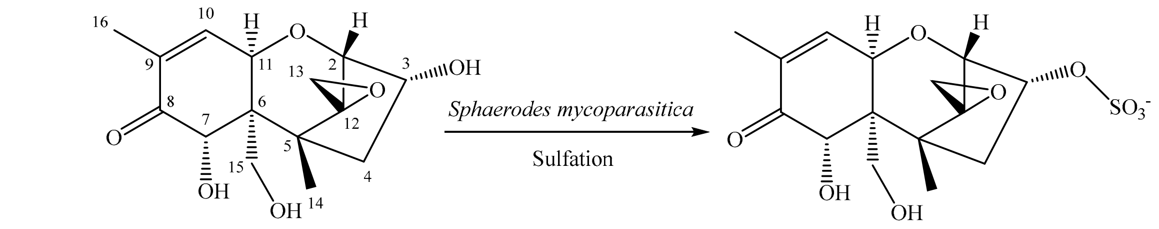

38. Deoxynivalenol **126**. Deoxynivalenol 3-sulfate

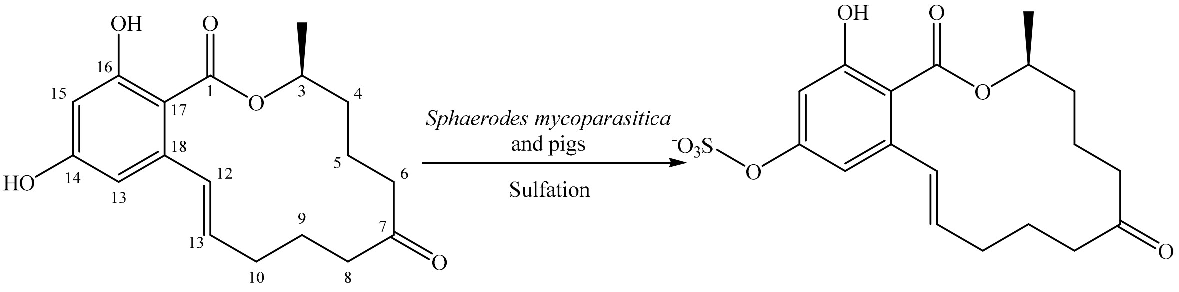

33. Zearalenone **127**. Zearalenone 14-sulfate

Figure S63. Both deoxynivalenol (DON, **38**) and zearalenone (ZEN, **33**) were converted to their corresponding sulfates DON 3-sulfate (**126**) and ZEN 14-sulfate (**127**) by the fungus *Sphaerodes mycoparasitica* [59]. ZEN (**33**) was converted to ZEN 14-sulfate (**127**) by pigs [60].

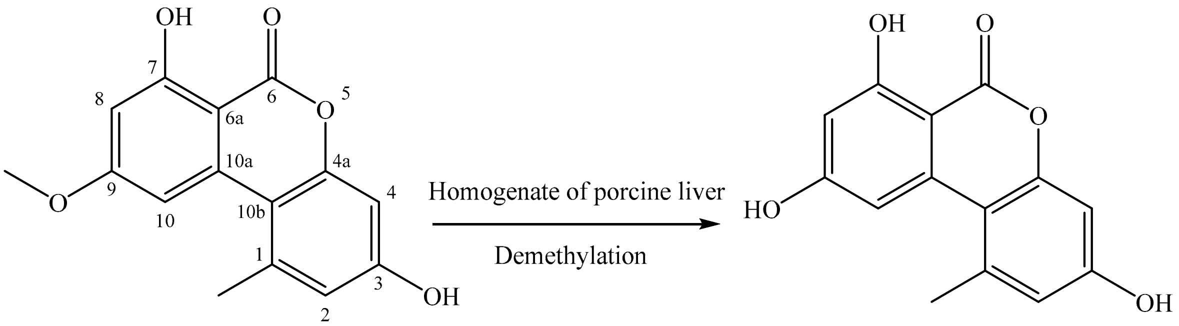

8. AME **7**. AOH

Figure S64. Transformation of alternariol 9-*O*-methylether (AME, **8**) with demethylation by the homogenate of porcine liver in the presence of NADPH [61].

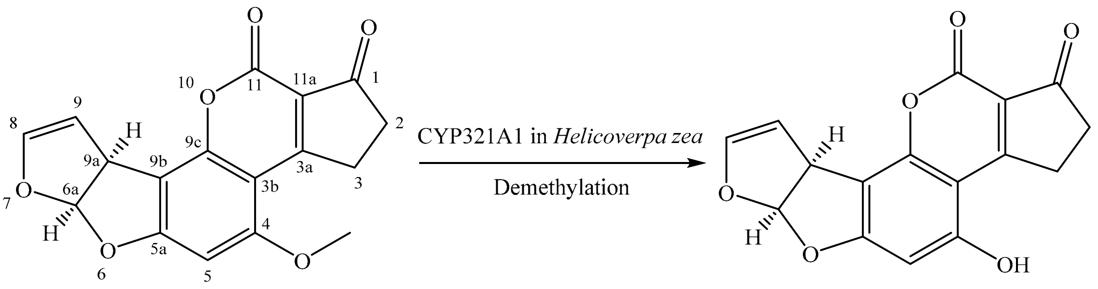

1. Aflatoxin B_1 **128**. Aflatoxin P_1

Figure S65. Transformation of aflatoxin B_1 (**1**) with demethylation by CYP21A1 in *Helicoverpa zea* [62].

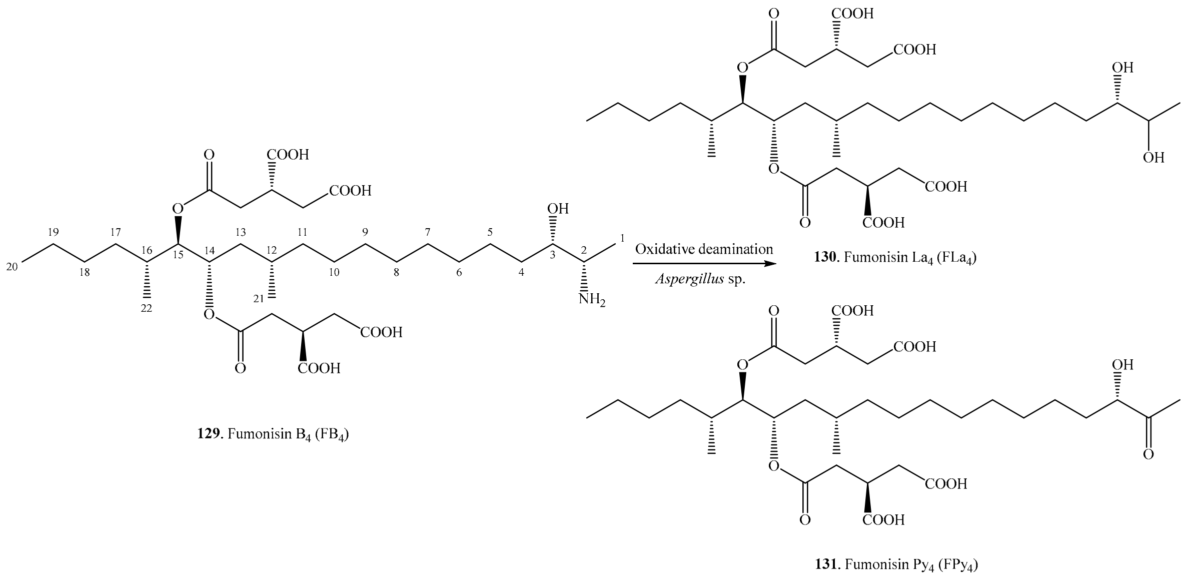

Figure S66. Transformation of fumonisin B4 (**129**) with oxidative deamination by *Aspergillus* sp. [63].

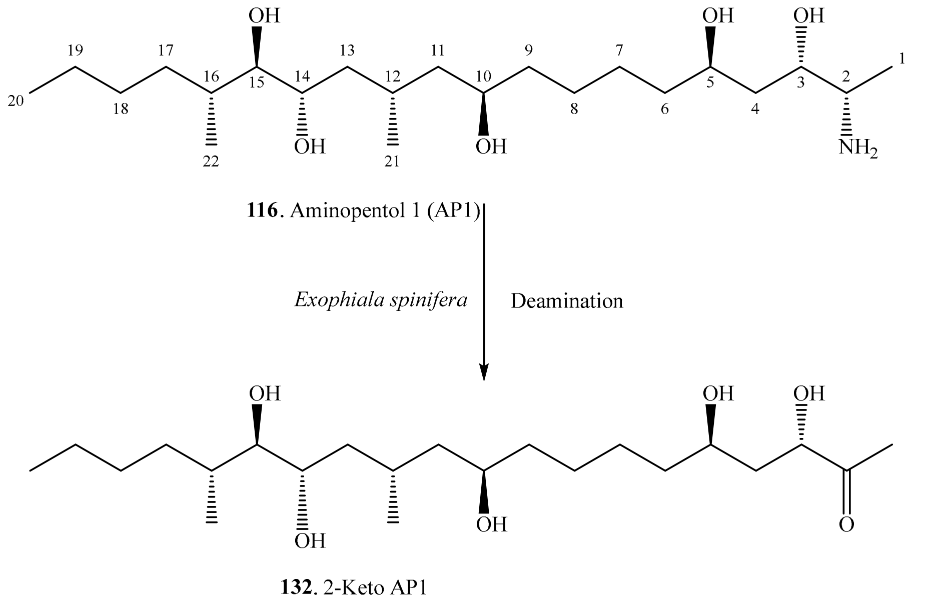

Figure S67. Transformation of aminopentol 1 (AP1, **116**) with oxidative deamination by *Exophiala spinifera* [64].

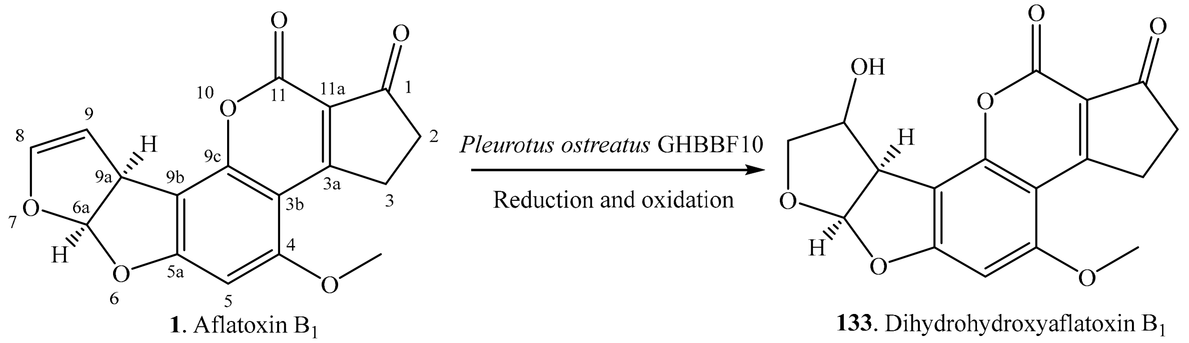

Figure S68. Transformation of aflatoxin B1 (**1**) with reduction and oxidation by *Pleurotus ostreatus* GHBBF10 [65].

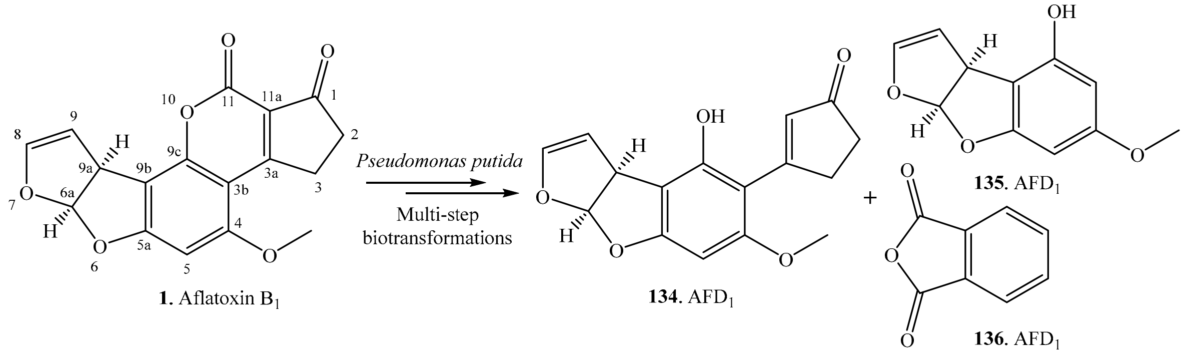

Figure S69. Transformation of aflatoxin B1 (**1**) with hydrolysis, decarboxylation and oxidation-reduction by *Pseudomonas putida* [66].

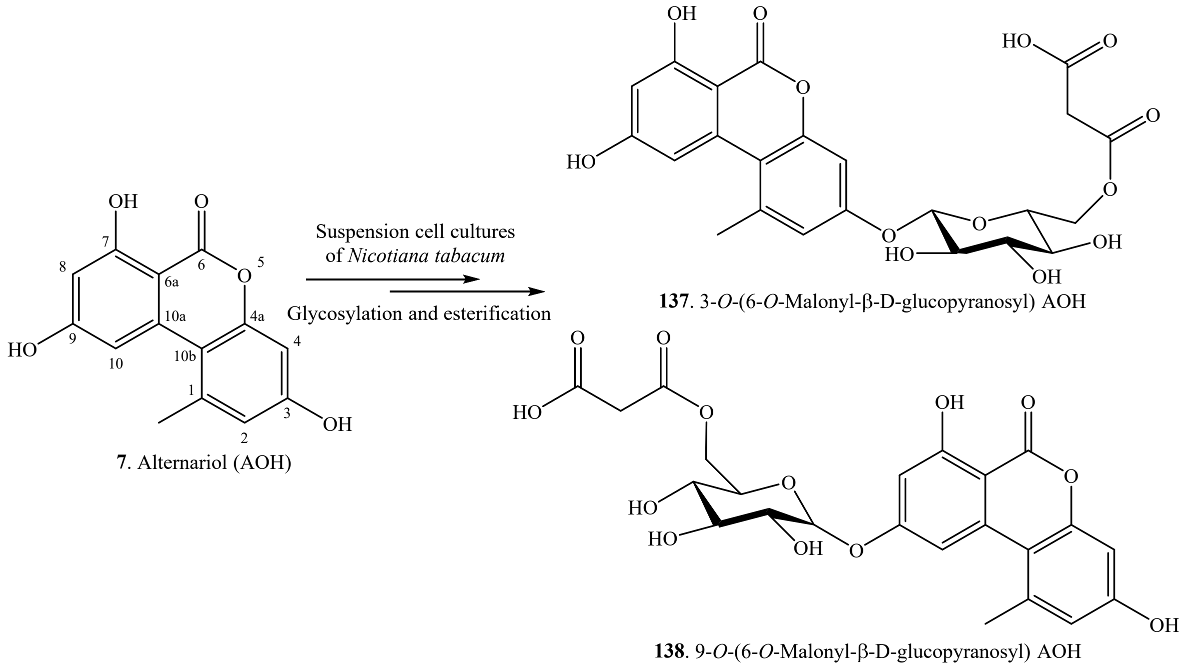

Figure S70. Transformation of alternariol (**7**) through glycosylation and esterification by suspension cell cultures of *Nicotiana batacum* [33].

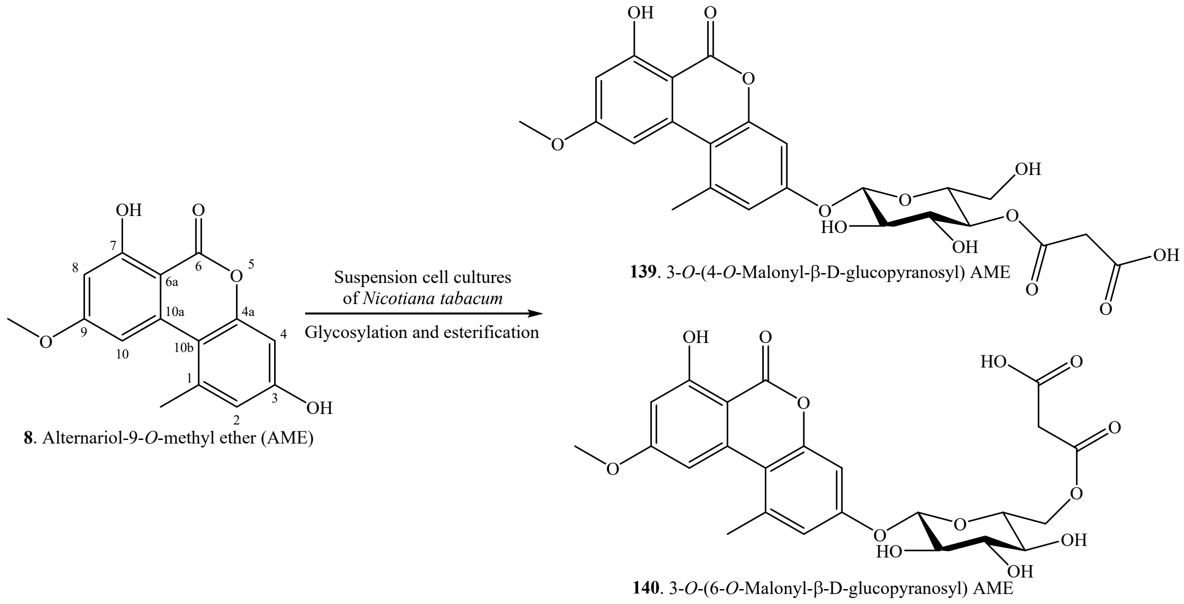

Figure S71. Transformation of alternariol 9-*O*-methyl ether (**8**) through glycosylation and esterification by suspension cell cultures of *Nicotiana batacum* [33].

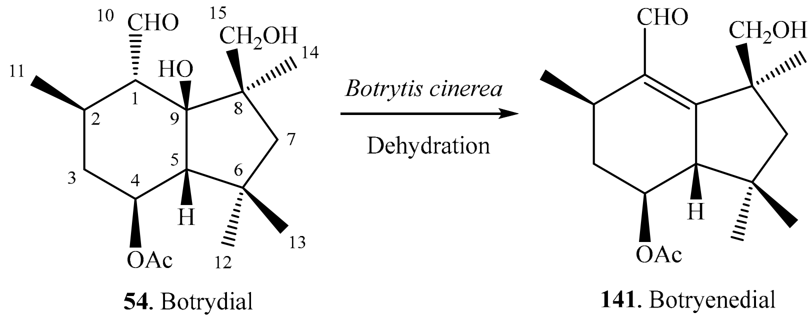

Figure S72. Transformation of botrydial (**54**) with dehydration by *Botrytis cinerea* [25].

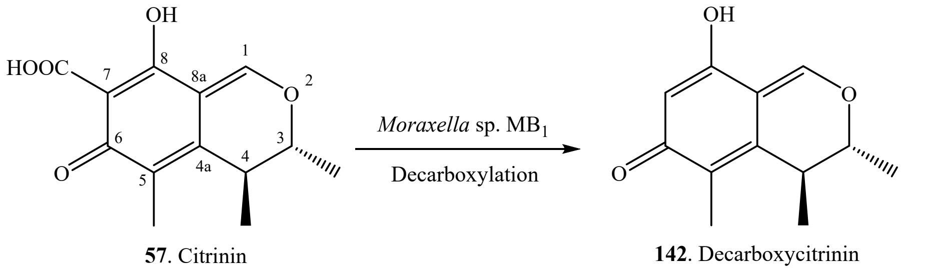

Figure S73. Transformation of citrinin (**57**) with decarboxylation by *Moraxella* sp. MB1 [67].

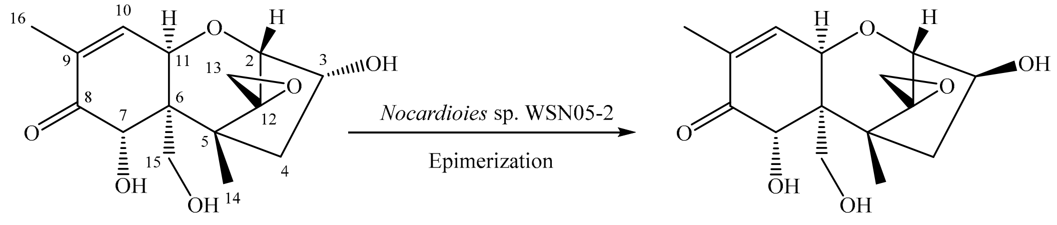

38. Deoxynivalenol

143. 3-*epi*-Deoxynivalenol

Figure S74. Transformation of deoxynivalenol (**38**) with epimerization by *Nocardioies* sp. WSN05-2 [68].

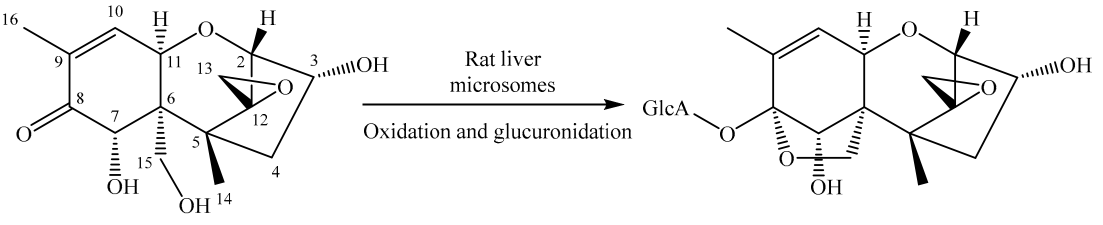

38. Deoxynivalenol (DON)

144. DON-8,15-hemiketal-8-GlcA

Figure S75. Transformation of deoxynivalenol (DON, **38**) with oxidation and glucuronidation by rate liver microsomes [37,38].

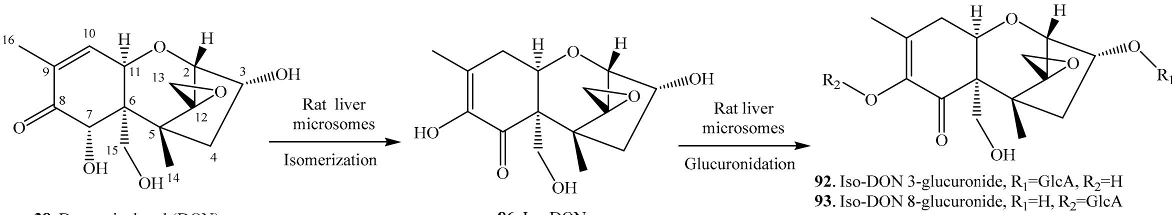

38. Deoxynivalenol (DON)

86. Iso-DON

92. Iso-DON 3-glucuronide, R_1=GlcA, R_2=H
93. Iso-DON 8-glucuronide, R_1=H, R_2=GlcA

Figure S76. Transformation of deoxynivalenol (DON, **38**) with isomerization and glucuronidation by rate liver microsomes [37,38].

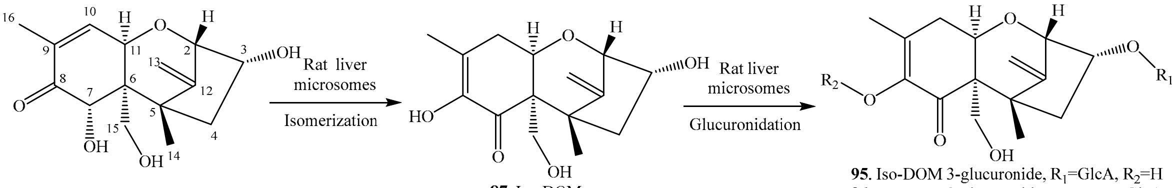

45. De-epoxydeoxynivalenol (DOM)

87. Iso-DOM

95. Iso-DOM 3-glucuronide, R_1=GlcA, R_2=H
96. Iso-DOM 8-glucuronide, R_1=H, R_2=GlcA

Figure S77. Transformation of deepoxy-deoxynivalenol (DOM, **45**) with isomerization and glucuronidation by rate liver microsomes [37,38].

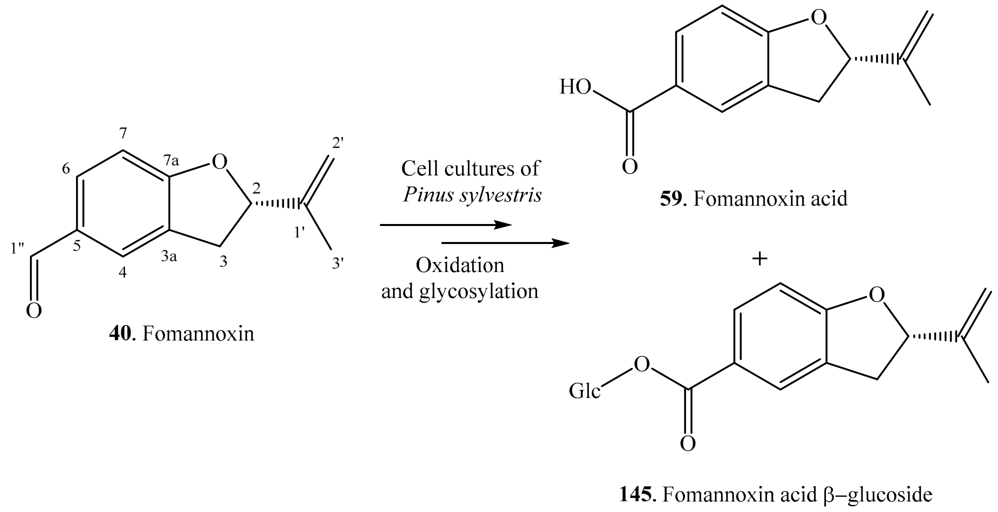

Figure S78. Transformation of fomannoxin (**40**) with oxidation and glycosylation by cell cultures of *Pinus sylvestris* [16].

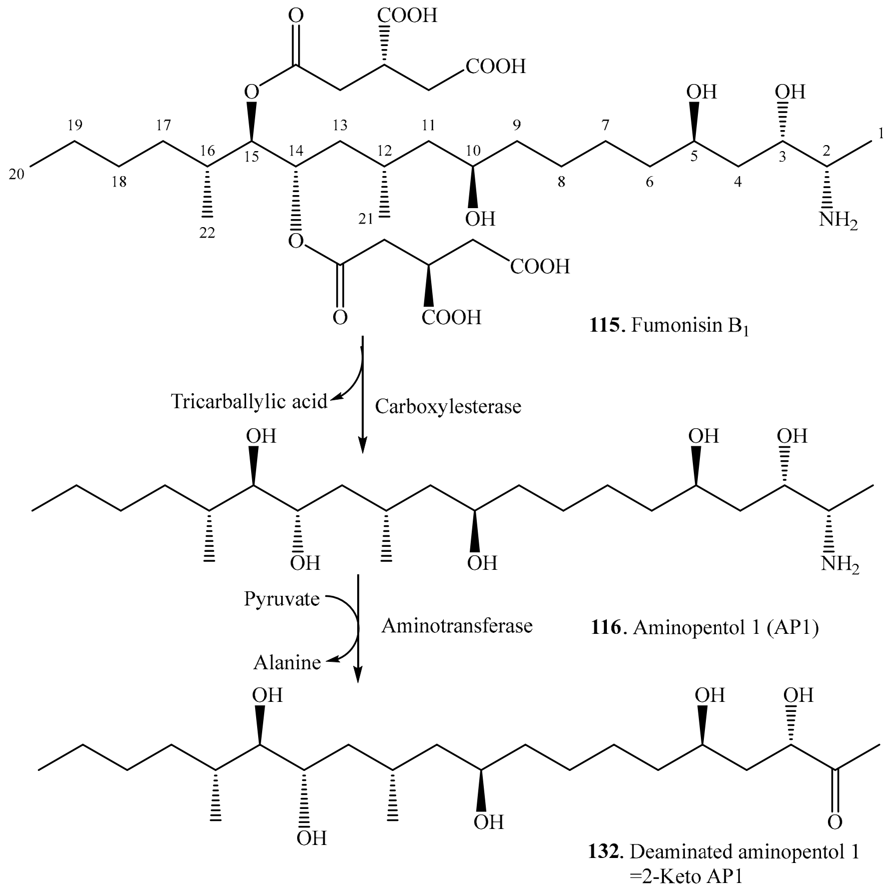

Figure S79. Transformation of fumonisin B1 (**115**) with hydrolysis and deamination by the recombinant enzymes from the bacterium *Sphingopyxis* sp. MTA144 [69].

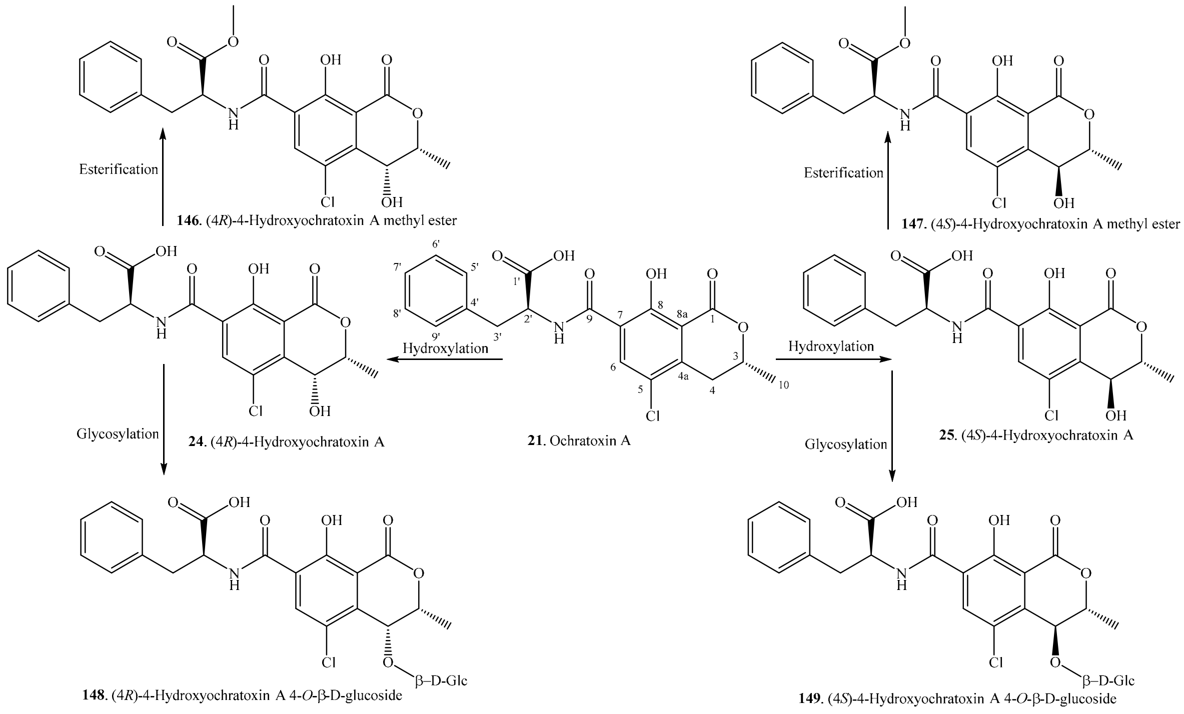

Figure S80. Transformation of ochratoxin A (**21**) with hydroxylation further esterification or glycosylation by the cell cultures of wheat and maize [46].

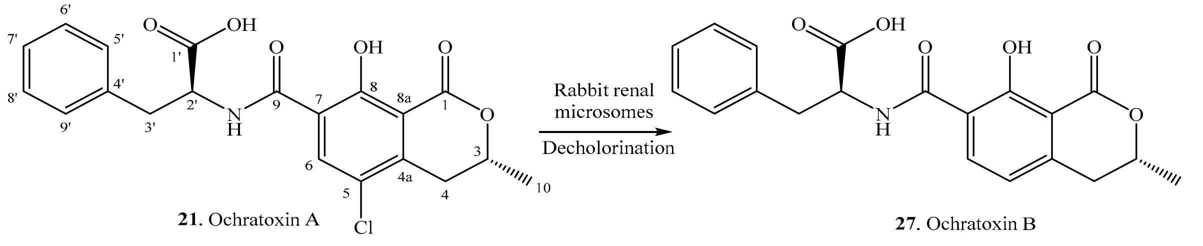

Figure S81. Transformation of ochratoxin A (**21**) with dechlorination in the renal microsomes [70].

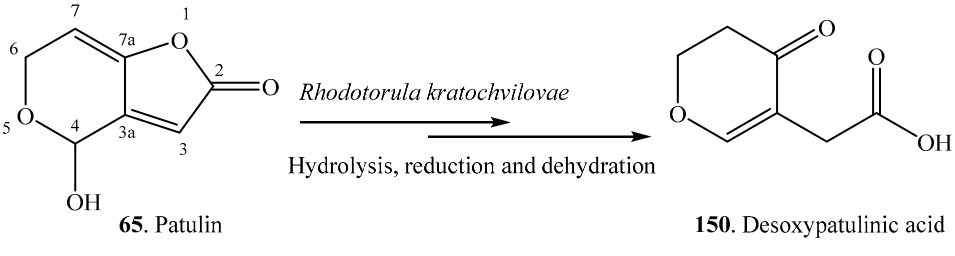

Figure S82. Transformation of patulin (**65**) with hydrolysis, reduction and dehydration by *Rhodotorla kratochvilovae* [71].

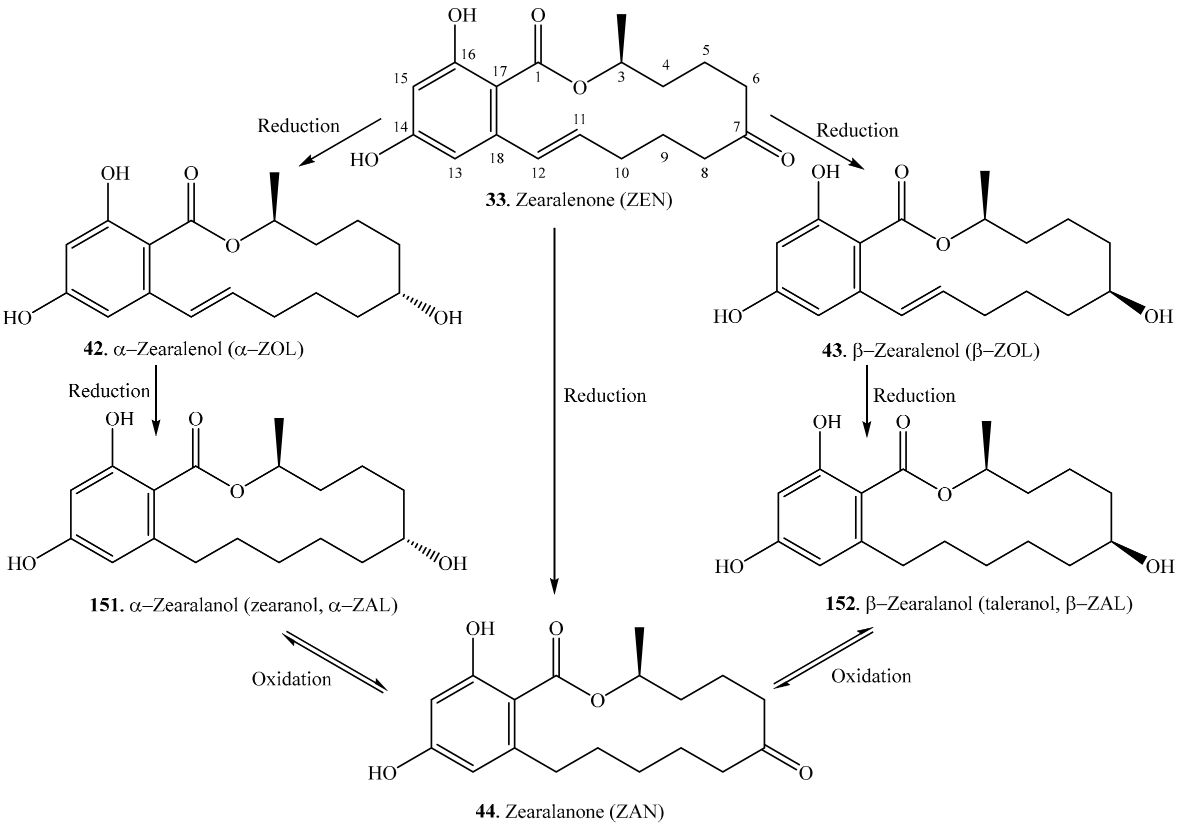

Figure S83. Transformation of zearalenone (**33**) with multi-step oxido-reductions in human body [72].

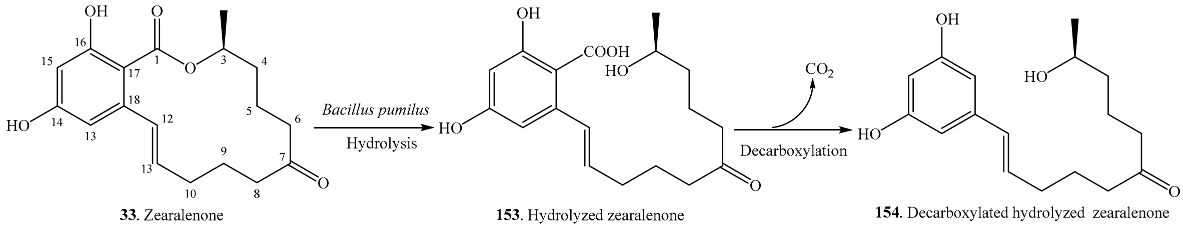

Figure S84. Transformation of zearalenone (**33**) with hydrolysis and decarboxylation by *Bacillus pumilus* [42].

Supplementary References

1. Krieger, R.I.; Salhab, A.S.; Dalezios, J.I.; Hseh, D.P.H. Aflatoxin B_1 hydroxylation by hepatic microsomal preparations from the rhesus money. *Food Cosmet. Toxicol.* **1975**, *13*, 211–219.
2. Wu, Q.; Jezkova, A.; Yuan, Z.; Pavlikova, L.; Dohnal, V.; Kuca, K. Biological degradation of aflatoxins. *Drug Metab. Dev.* **2009**, *41*, 1–7.
3. Pfeiffer, E.; Schebb, N.H.; Podlech, J.; Metzler, M. Novel oxidative *in vitro* metabolites of the mycotoxins alternariol and alternariol methyl ether. *Mol. Nutr. Food Res.* **2007**, *51*, 307–316.
4. Pedras, M.S.C.; Zaharia, I.L.; Gai, Y.; Zhou, Y.; Ward, D.E. *In planta* sequential hydroxylation and glycosylation of a fungal phytotoxin: avoiding cell death and overcoming the fungal invader. *Proc. Nat. Acad. Sci. U. S. A.* **2001**, *98*, 747–752.
5. Crutcher, F.K.; Puckhaber, L.S.; Bell, A.A.; Liu, J.; Duke, S.E.; Stipanovic, R.D.; Nichols, R.L. Detoxification of fusaric acid by the soil microbe *Mucor rouxii*. *J. Agric. Food Chem.* **2017**, *65*, 4989–4992.
6. Wegst, W.; Lingens, F. Bacterial degradation of ochratoxin A. *FEMS Microbiol. Lett.* **1983**, *17*, 341–344.
7. Stormer, F.C.; Pedersen, J.I. Formation of 4-hydroxyochratoxin A from ochratoxin A by rat liver microsomes. *Appl. Environ. Microbiol.* **1980**, *39*, 971–975.

8. Stormer, F.C.; Storen, O.; Hansen, C.E.; Pedersen, J.I.; Aasen, A.J. Formation of (4*R*)- and (4*S*)-4-hydroxyochratoxin A and 10-hydroxyochratoxin A from ochratoxin A by rabbit liver microsomes. *Appl. Environ. Microbiol*. **1983**, *45*, 1183–1187.
9. Mally, A.; Zepnik, H.; Wanek, P.; Eder, E.; Dingley, K.; Ihmels, H. Volkel, W.; Dekant, W. Ochratoxin A: lack of formation of covalent DNA adducts. *Chem. Res. Toxicol.* **2004**, *17*, 234–242.
10. Pfeiffer, E.; Fleck, S.C.; Metzler, M. Catechol formation: a novel pathway in the metabolism of sterigmatocystin and 11-methoxysterigmatocystin. *Chem. Res. Toxicol*. **2014**, *27*, 2093–2099.
11. Yuan, Y.; Zhou, X.; Yang, J.; Li, M. Qiu, X. T-2 toxin is hydroxylated by chicken CYP3A37. *Food Chem. Toxicol*. **2013**, *62*, 622–627.
12. El-Sharkawy, S.H.; Abul-Hajj, Y.J. Microbial transformation of zearalenone. 2. Reduction, hydroxylation, and methylation products. *J. Org. Chem*. **1988**, *53*, 515–519.
13. Pfeiffer, E.; Hildebrand, A.; Damm, G.; Rapp, A.; Cramer, B.; Uumpf, H.-U.; Metzler, M. Aromatic hydroxylation is a major metabolic pathway of the mycotoxin zearalenone *in vitro*. *Mol. Nutr. Food Res*. **2009**, *53*, 1123–1133.
14. Nakazato, M.; Morozumi, S.; Saito, K.; Fujinuma, K.; Nishima, T.; Kasai, N. Interconversion of aflatoxin B_1 and aflatoxicol by several fungi. *Appl. Environ. Microbiol*. **1990**, *56*, 1465–1470.
15. Carere, J.; Hassan, Y.I.; Lepp, D.; Zhou, T. The enzymatic detoxification of the mycotoxin deoxynivalenol: identification of DepA from the DON epimerization pathway. *Microb. Biotechnol.* **2018**, *11*, 1106–1111.
16. Zweimuller, M.; Antus, S.; Kovacs, T.; Sonnenbichler, J. Biotransformation of the fungal toxin formannoxin by conifer cell cultures. *Biol. Chem*. **1997**, *378*, 915–921.
17. Palyusik, M.; Hagler, W.M.; Horvath, L.; Microcha, C.J. Biotransformationi of zearalenone to zearalenol by *Candida tropicalis*. *Acta Veterinaria Academiae Scientiarum Hugaricae* **1980**, *28*, 159–166.
18. Miles, C.O.; Erasmuson, A.F.; Wilkins, A.L.; Towers, N.R.; Smith, B.L.; Garthwaite, I.; Scahill, B.G.; Hansen, R.P. Ovine metabolism of zearalenone to α-zearalanol (zeranol). *J. Agric. Food Chem.* **1996**, *44*, 3244–3250.
19. Binder, J.; Horvath, E.M.; Schatzmayr, G.; Ellend, N.; Danner, H.; Krska, R.; Braun, R. Screening for deoxynivalenol-detoxifying anaerobic rumen microorganisms. *Cereal Res. Commun*. **1997**, *25*, 343–346.
20. Fuchs, E.; Binder, E.M.; Heidler, D.; Krska, R. Characterisation of metabolites after the microbial degradation of A- and B-trichothecenes by BBSH797. *Mycotoxin Res*. **2000**, *16*, 66–69.
21. Onji, Y.; Dohi, Y.; Aoki, Y.; Moriyama, T.; Nagami, H.; Uno, M.; Tanaka, T.; Yamazoe, Y. Deepoxynivalenol: a new metabolite of nivalenol found in the excreta of orally administered rats. *J. Agric. Food Chem*. **1989**, *37*, 478–481.
22. Gallagher, E.P.; Eaton, D.L. *In vitro* biotransformation of aflatoxin B_1 (AFB_1) in channel catfish liver. *Toxicol. Appl. Pharmacol*. **1995**, *132*, 82–90.
23. Wang, J.; Ogata, M.; Hirai, H.; Kawagishi, H. Detoxification of aflatoxin B_1 by manganese peroxidase from the white-rot fungus *Phanerochaete sordida* YK-624. *FEMS Microbiol. Lett*. **2011**, *314*, 164–169.
24. Fleck, S.C.; Pfeiffer E.; Podlech, J.; Metzler, M. Epoxide reduction to an alcohol: a novel metabolic pathway for perylene quinone-type *Alternaria* mycotoxins in mammalian cells. *Chem. Res. Toxicol*. **2014**, *27*, 247–253.
25. Daoubi, M.; Duran-Patron, R.; Hernandez-Galan, R.; Benharref, A.; Hanson, J.R.; Collado, I.G. The role of botrydienediol in the biodegradation of the sesquiterpenoids phytotoxin botrydial by *Botrytis cinerea*. *Tetrahedron* **2006**, *62*, 8256–8261.
26. Dunn, B.B.; Stack, M.E.; Park, D.L.; Joshi, A.; Friedman, L.; King, R.L. Isolation and identification of dihydrocitrinone, a urinary metabolite of citrinin in rats. *J. Toxicol. Environ. Health* **1983**, *12*, 283–289.
27. Follmann, W.; Behm, C.; Degen, G.H. Toxicity of the mycotoxin citrinin and its metabolite dihydrocitrinoine and of mixtures of citrinin and ochratoxin A *in vitro*. *Arch. Toxicol*. **2014**, *88*, 1097–1107.
28. Horlacher, N.; Nachtigall, J.; Schulz, D.; Sussmuth, R.D.; Hampp, R.; Fiedler, H.-P.; Schrey, S.D. Biotransformation of the fungal phytotoxin fomannoxin by soil streptomycetes. *J. Chem. Ecol*. **2013**, *39*, 931–941.
29. Crutcher, F.K.; Liu, J.; Puckhaber, L.S.; Stipanovic, R.D.; Duke, S.E.; Bell, A.A.; Williams, H.J.; Nichols, R.L. Conversion of fusaric acid to fusarinol by *Aspergillus tubingensis*: a detoxification reaction. *J. Chem. Ecol*. **2014**, *40*, 84–89.
30. Dong, X.; Jiang, W.; Li, C.; Ma, N.; Xu, Y.; Meng, X. Patulin biodegradation by marine yeast *Kodameae ohmeri*. *Food Addit. Contam. A* **2015**, *32*, 352–360.
31. Hawar, S.; Vevers, W.; Karieb, S.; Ali, B.K.; Billington, R.; Beal, J. Biotransformation of patulin to hydroascladiol by *Lactobacillus plantarum*. *Food Control* **2013**, *34*, 502–508.

32. Stinson, E.E.; Moreau, R.A. Partial purification and some properties of an alternariol-*O*-methyltransferase from *Alternaria tenuis*. *Phytochemistry* **1986**, *25*, 2721–2724.
33. Hildebrand, A.A.; Kohn, B.N.; Pfeiffer, E.; Wefers, D.; Metzler, M.; Bunzel M. Conjugation of the mycotoxins alternariol and alternariol monomethyl ether in tobacco suspension cells. *J. Agric. Food Chem.* **2015**, *63*, 4728–4736.
34. Soukup, S.T.; Kohn, B.N.; Pfeiffer, E.; Geisen, R.; Metzler, M.; Bunzel, M.; Kulling, S.E. Sulfoglucosides as novel modified forms of the mycotoxins alternariol and alternariol monomethyl ether. *J. Agric. Food Chem.* **2016**, *64*, 8892–8901.
35. Zhan, J.; Gunatilak, A.A.L. Microbial transformation of curvularin. *J. Nat. Prod*. **2005**, *68*, 1271–1273.
36. Michlmayr, H.; Malachova, A.; Varga, E.; Kleinova, J.; Lemmens, M.; Newmister, S.; Rayment, I.; Berthiller, F.; Adam, G. Biochemical characterization of a recombinant UDP-glucosyltransferase from rice and enzymatic production of deoxynivalenol-3-*O*-β-D-glucoside. *Toxins* **2015**, *7*, 2685–2700.
37. Schwartz-Zimmermann, H.E.; Hametner, C.; Nagl, V; Fiby, I.; Macheiner, L.; Winkler, J.; Danicke, S.; Clark, E.; Pestka, J.J.; Berthiller, F. Glucuronidation of deoxynivalenol (DON) by different animal species: identification of iso-DON glucuronides and iso-deepoxy-DON glucuronides as novel DON metabolites in pigs, rats, mice, and cows. *Arch. Toxcol*. **2017**, *91*, 3857–3872.
38. Schwartz-Zimmermann, H.E.; Hametner, C.; Nagl, V; Fiby, I.; Macheiner, L.; Winkler, J.; Danicke, S.; Clark, E.; Pestka, J.J.; Berthiller, F. Correction to: Glucuronidation of deoxynivalenol (DON) by different animal species: identification of iso-DON glucuronides and iso-deepoxy-DON glucuronides as novel DON metabolites in pigs, rats, mice, and cows. *Arch. Toxcol*. **2018**, *92*, 3245–3246.
39. Nakagawa, H.; Sakamoto, S.; Sago, Y.; Kushiro, M.; Nagashima, H. Detection of masked mycotoxins derived from type A trichothecenes in corn by high-resolution LC-Orbitrap mass spectrometer. *Food Addit. Contam. A* **2013**, *30*, 1407–1414.
40. Roush, W.R.; Marletta, M.A.; Russo-Rodriguez, S.; Recchia, J. Trichotecene metabolism studies: isolation and structure determination of 15-acetyl-3α-(1′β-D-glucopyranosiduronyl)-scirpen-3,4β,15-triol. *J. Am. Chem. Soc*. **1985**, *107*, 3354–3355.
41. McCormick, S.P.; Price, N.P.J.; Kurtzman, C.P. Glucosylation and other biotransformations of T-2 toxin by yeasts of the *Trichomonascus* Clade. *Appl. Environ. Microbiol*. **2012**, *78*, 8694–8702.
42. Wang, X.; Wang, Y.; Wang, Y.; Sun, L.; Gooneratne, R. Preparation of T-2-glucoronide with rat hepatic microsomes and its use along with T-2 for activation of the JAK/STAT signaling pathway in RAW264.7 cells. *J. Agric. Food Chem*. **2017**, *65*, 4811–4818.
43. Poppenberger, B.; Berthiller, F.; Bachmann, H.; Lucyshyn, D.; Peterbauer, C.; Mitterbauer, R.; Schuhmacher, R.; Krska, R.; Glossl, J.; Adam, G. Heterologous expression of *Arabidopsis* UDP-glucosyltransferases in *Saccharomyces cerevisiae* for production of zearalenone-4-*O*-glucoside. *Appl. Environ. Microbiol.* **2006**, *72*, 4404–4410.
44. Paris, M.P.K.; Schweiger, W.; Hametner, C.; Stuckler, R.; Muehlbauer, G.J.; Varga, E.; Krska, R.; Berthiller, F.; Adam, G. Zearalenone-16-*O*-glucoside: a new masked mycotoxin. *J. Agric. Food Chem*. **2014**, *62*, 1181–1189.
45. Michlayr, H.; Varga, E.; Lupi, F.; Malachova, A.; Hametner, C.; Berthiller, F.; Adam, G. Synthesis of mono- and di-glucosides of zearalenone and α/β-zearalenol by recombinant barley glucosyltransferase *Hv*UGT14077. *Toxins* **2017**, *9*, 58.
46. Ruhland, M.; Engelhardt, G.; Wallnofer, P.R.; Schafer, W. Transformation of the mycotoxin ochratoxin A in wheat and maize cell suspension cultures. *Naturwissenschaften* **1994**, *81*, 453–454.
47. Munger, C.E.; Ivie, G.W.; Christopher, R.J.; Hammock, B.D.; Phillips, T.D. Acetylation/deacetylation reactions of T-2, acetyl T-2, HT-2, and acetyl HT-2 toxins in bovine rumen fluid *in vitro*. *J. Agric. Food Chem.* **1987**, *35*, 354–358.
48. Udell, M.N.; Dewick, P.M. Metabolic conversions of trichothecene mycotoxins: de-esterification reactions using cell-free extracts of *Fusarium*. *Z. Naturforsch. C* **1989**, *44*, 660–668.
49. Duvick, J.; Rood, T.; Maddox, J.; Gilliam, J. Detoxification of mycotoxins in planta as a strategy for improving grain quality and disease resistance: identification of fumonisin-degrading microbes from maize. *Dev. Plant Pathol*. **1998**, *13*, 369–381.
50. Poapolathep, A.; Singhasem, S.; Noonpugdee, C.; Sugita-Konishi, Y.; Doi, K.; Kumagai, S. The fate and transmission of fusarenon-X (FX), a trichothecene mycotoxin in mice. *Toxicol. Appl. Pharmacol*. **2004**, *197*, 367–367.

51. Phruksawan, W.; Poapolathep, S.; Giorgi, M.; Imsilp, K.; Sakulthaew, C.; Owen, H.; Poapolathep, A. Toxicokinetic profile of fusarenon-X and its metabolite nivalenol in the goat (*Capra hircus*). *Toxicon* **2018**, *153*, 78–84.
52. Stander, MA.; Bornscheuer, U.T.; Henke, E.; Steyn, P.S. Screening of commercial hydrolases for the degradation of ochratoxin A. *J. Agric. Food Chem.* **2000**, *48*, 5736–5739.
53. Abrunhosa, L.; Santos, L.; Verancio, A. Degradation of ochratoxin by proteases and by a crude enzyme of *Aspergillus niger*. *Food Biotechnol.* **2006**, *20*, 231–240.
54. Pitout, M.J. The hydrolysis of ochratoxin A by some proteolytic enzymes. *Biochem. Pharmacol.* **1969**, *18*, 485–491.
55. Li, S.; Marquardt, R.R.; Frohlich, A.A.; Vitti, T.G.; Crow, G. Pharmacokinetics of ochratoxin A and its metabolites in rats. *Toxicol. Appl. Pharmacol.* **1997**, *145*, 82–90.
56. Fuchs, R.; Hult, K.; Peraica, M.; Razica, R.; Plestina, R. Conversion of ochratoxin C into ochratoxin A *in vivo*. *Appl. Environ. Microbiol.* **1984**, *48*, 41–42.
57. Fuchs, E.; Binder, E.M.; Heiler, D.; Krska, R. Structural characterization of metabolites after the microbial degradation of type A trichothecenes by the bacterial strain BBSH 797. *Food Addi. Contam.* **2002**, *19*, 379–386.
58. Yoshizawa, T.; Swanson, S.P.; Mirocha, C.J. *In vitro* metabolism of T-2 toxin in rats. *Appl. Environ. Microbiol.* **1980**, *40*, 901–906.
59. Kim, S.H.; Vujanovic, V. Biodegradation and biodetoxification of *Fusarium* mycotoxins by *Sphaerodes mycoparasitica*. *AMB Expr.* **2017**, *7*, 145.
60. Catteuw, A.; Broekaert, N.; De Baere, S.; Lauwers, M.; Gasthuys, E.; Huybrechts, B.; Callebaut, A.; Ivanova, L.; Uhlig, S.; De Boevre, M.; et al. Insights into *in vivo* absolute oral bioavailability, biotransformation, and toxicokinetics of zearalenone, α-zearalenol, β-zearalenol, zearalenone-14-glucosdie, and zearalenone-14-sulfate in pigs. *J. Agric. Food Chem.* **2019**, *67*, 3448–3458.
61. Olsen, M.; Visconti, A. Metabolism of alternariol monomethylether by porcine liver and intestinal mucosa *in vitro*. *Toxicol. In Vitro* **1988**, *2*, 27-29.
62. Niu, G.; Wen, Z.; Rupasinghe, S.G.; Zeng, R.S.; Berenbaum, M.R.; Schuler, M.A. Aflatoxin B_1 detoxification by CYP321A1 in *Helicomverpa zea*. *Arch. Insect Biochem. Physiol.* **2008**, *69*, 32–45.
63. Burgess, K.M.N.; Renaud, J.B.; McDowell, T.; Sumarah, M.W. Mechanistic insight into the biosynthesis and detoxification of fumonisin mycotoxins. *ACS Chem. Biol.* **2016**, *11*, 2618–2625.
64. Blackwell, B.A.; Gilliam, J.T.; Savard, M.E.; Miller, D.; Duvick, J.P. Oxidative deamination of hydrolyzed fumonisin B_1 (AP_1) by cultures of *Exophiala spinifera*. *Nat. Toxins* **1999**, *7*, 31–38.
65. Das, A.; Bhattacharya, S.; Palaniswamy, M.; Angayarkanni, J. Biodegradation of aflatoxin B_1 incontaminated rice straw by *Pleurotus ostreatus* MTCC142 and *Pleurotus ostreatus* GHBBF10 in the presence of metal salts and surfactants. *World J. Microbiol. Biotechnol.* **2014**; *30*, 2315–2324.
66. Samuel, M.S.; Sivaramakrishna, A.; Mehta, A. Degradation and detoxification of aflatoxin B_1 by *Pseudomonas putida*. *Int. Biodeterior. Biodegr.* **2014**, *86*, 202–209.
67. Devi, P.; Naik, C.G.; Rodrigues, C. Biotransformation of citrinin to decarboxycitrinin using an organic solvent-tolerant marine bacterium, *Moraxella* sp. MB1. *Mar. Biotechnol.* **2006**, *8*, 129–138.
68. Ikunaga, Y.; Sato, I.; Grond, S.; Numaziri, N.; Yoshida, S.; Yamaya, H.; Hiradate, S.; Hasegawa, M.; Toshima, H.; Koitabashi, M. *Nocardioides* sp. strain WSN05-2, isolated from a wheat field, degrades deoxynivalenol, producing the novel intermediate 3-*epi*-deoxynivalenol. *Appl. Microbiol. Biotechnol.* **2011**, *89*, 419–427.
69. Heinl, S.; Hartinger, D.; Thamhesl, M.; Vekiru, E.; Krska, R.; Schatzmayr, G.; Moll, W.-D.; Grabherr, R. Degradation of fumonisin B_1 by the consecutive action of two bacterial enzymes. *J. Biotechnol.* **2010**, *145*, 120–129.
70. Adlouni, C.E.; Pinelli, E.; Azemar, B.; Zaoui, D.; beaune, P.; Pfohl-Leszkowicz, A. Phenobarbital increases DNA adduct and metabolites formed by ochratoxin A: role of CYP 2C9 and microsomal glutathione-*S*-transferase. *Environ. Mol. Mutagen.* **2000**, *35*, 123–131.
71. Pinedo, C.; Wright, S.A.I.; Collado, I.G.; Goss, R.J.M.; Castoria, R.; Hrelia, P.; Maffei, F.; Duran-Patron, R. Isotopic labeling studies reveal the patulin detoxification pathway by the biocontrol yeast *Rhodotorula kratochvilovae* LS11. *J. Nat. Prod.* **2018**, *81*, 2692–2699.

72. Belhassen, H.; Jimenez-Diaz, I.; Ghali, R.; Ghorbel, H.; Molina-Molina, J.M. Validation of a UHPLC-MS/MS method for quantification of zearaenone, α-zearalenol, β-zearalenol, α-zearalanol, β-zearalanol and zearalanone in human urine. *J. Chromatogr. B* **2014**, *962*, 68–74.

© 2020 by the authors. Licensee MDPI, Basel, Switzerland. This article is an open access article distributed under the terms and conditions of the Creative Commons Attribution (CC BY) license (http://creativecommons.org/licenses/by/4.0/).
